# Mechanistic insights into boron-catalysed direct amidation reactions†
†Electronic supplementary information (ESI) available: FAIR data for NMR spectra, computational data and ESI for synthetic procedures. CCDC 1551614–1551624 and 1563948–1563950. For ESI and crystallographic data in CIF or other electronic format see DOI: 10.1039/c7sc03595k and ref. 26.


**DOI:** 10.1039/c7sc03595k

**Published:** 2018-01-02

**Authors:** Sergey Arkhipenko, Marco T. Sabatini, Andrei S. Batsanov, Valerija Karaluka, Tom D. Sheppard, Henry S. Rzepa, Andrew Whiting

**Affiliations:** a Centre for Sustainable Chemical Processes , Department of Chemistry , Durham University , Science Site , South Road , Durham , DH1 3LE , UK . Email: andy.whiting@durham.ac.uk; b Department of Chemistry , University College London , 20 Gordon Street , London , WC1H 0AJ , UK; c Department of Chemistry , Durham University , Science Site , South Road , Durham , DH1 3LE , UK; d Department of Chemistry , Imperial College London , South Kensington Campus , London , SW7 2AZ , UK

## Abstract

The generally accepted monoacyloxyboron mechanism of boron-catalysed direct amidation is brought into question in this study, and new alternatives are proposed. We have carried out a detailed investigation of boron-catalysed amidation reactions, through study of the interaction between amines/carboxylic acids and borinic acids, boronic acids and boric acid, and have isolated and characterised by NMR/X-ray crystallography many of the likely intermediates present in catalytic amidation reactions. Rapid reaction between amines and boron compounds was observed in all cases, and it is proposed that such boron–nitrogen interactions are highly likely to take place in catalytic amidation reactions. These studies also clearly show that borinic acids are not competent catalysts for amidation, as they either form unreactive amino-carboxylate complexes, or undergo protodeboronation to give boronic acids. It therefore seems that at least three free coordination sites on the boron atom are necessary for amidation catalysis to occur. However, these observations are not consistent with the currently accepted ‘mechanism’ for boron-mediated amidation reactions involving nucleophilic attack of an amine onto a monomeric acyloxyboron intermediate, and as a result of our observations and theoretical modelling, alternative proposed mechanisms are presented for boron-mediated amidation reactions. These are likely to proceed *via* the formation of a dimeric B–X–B motif (X = O, NR), which is uniquely able to provide activation of the carboxylic acid, whilst orchestrating the delivery of the amine nucleophile to the carbonyl group. Quantum mechanical calculations of catalytic cycles at the B3LYP+D3/Def2-TZVPP level (solvent = CH_2_Cl_2_) support the proposal of several closely related potential pathways for amidation, all of which are likely to be lower in energy than the currently accepted mechanism.

## Abstract 2

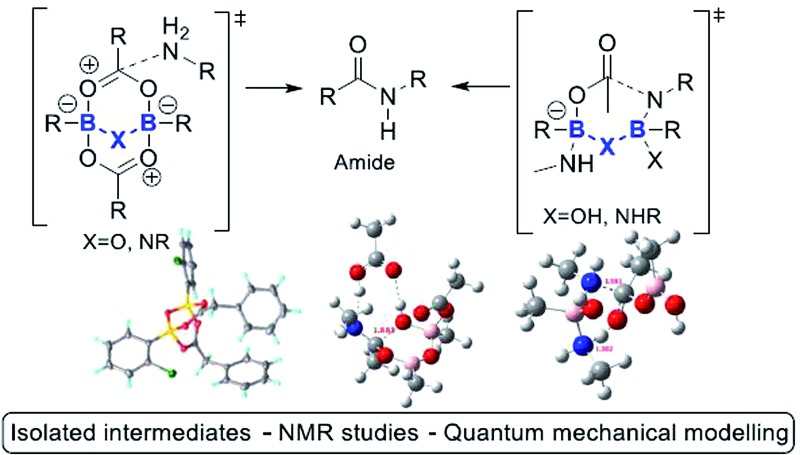
The generally accepted monoacyloxyboron mechanism of boron-catalysed direct amidation is brought into question in this study, and new alternatives are proposed.

## Introduction

Boron compounds have a rich, fascinating and complex structural chemistry, especially related to clusters, cages, complexes and anions associated with B–O^1^ and B–N^2^ containing systems. Indeed, such systems are well known to “have a distinct tendency to disproportionate, apparently more so than those of any other nonmetallic elements”.1*a* This observation means that identifying and controlling the role played by boron in reactions and catalytic processes is particularly challenging and has proved so on many occasions.

**Scheme 1:**
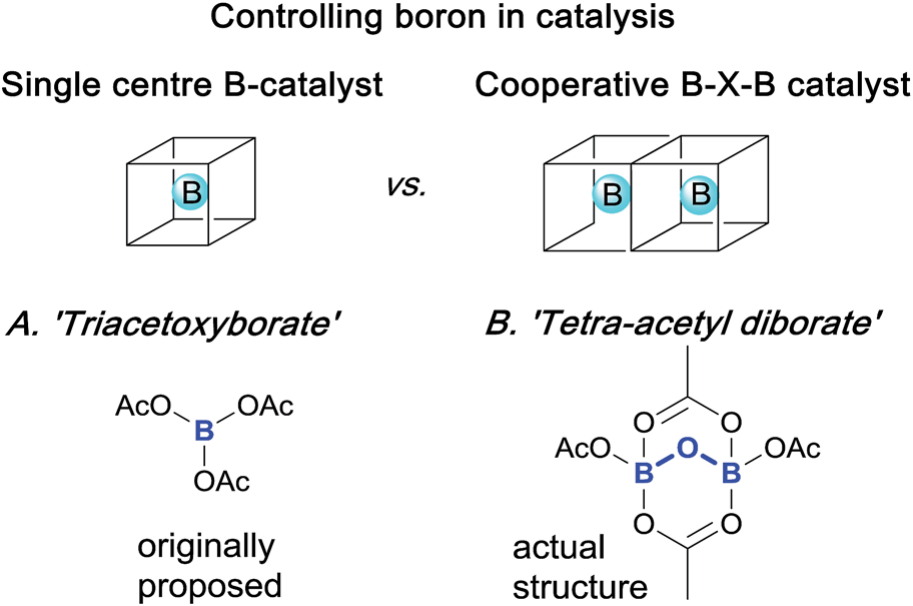
General structural role of boron in different catalytic processes.

Elegant single and cooperative solutions, exemplified in Scheme 1, have enabled the precise reactivity control of boron in different reactions. For example, in the catalytic asymmetric CBS reduction system^3^ both nitrogen and oxygen ligands control boron coordination to tune an external boron to behave cooperatively to control ketone Lewis-acid binding, activation and hence, reduction. Similarly, precise boron centre control can be employed to achieve highly efficient hydroxynaphthaquinone chiral Lewis-acid binding, activation and subsequent asymmetric Diels–Alder cycloaddition;^4^ the boron control and prevention of diborate-disproportionation deriving from use of hindered BINOL ligands, assembled by careful manipulation of unstable acyloxyboron species. Indeed, the model for these boron complexes was derived from ‘triacetoxyborate’ complexes of chiral juglone derivatives. However, triacetoxyborate is not stable at ambient temperatures and it rearranges to give tetra-acetyl diborate, as confirmed by X-ray crystallography.^5^ These seminal works led us to conclude that reaction mechanisms involving boron are non-trivial, because the structural chemistry of boron is itself non-trivial.

It is generally the case that in the absence of a catalyst, amines and carboxylic acids do not usually react to form amides at ambient temperatures, and forcing conditions are often required to promote this condensation reaction to access amides.^6^ Although a direct thermally accessible reaction is possible, direct amidation is better facilitated at lower temperatures by using catalysts,^7^ with boron-based systems being particularly effective.^8^ Useful catalysts for direct amidation include boric acid,^9^ borate esters^10^ and boronic acids.^11^–^13^ Borinic acids have also been claimed to promote direct amidation reactions under relatively mild conditions.^14^ Clearly, an understanding of the mechanism of these boron-mediated amidations is crucial for designing catalysts with improved activity, but more importantly, a fundamental understanding of the role of the boron in such systems is required. To this end, we have been interested in the structural and mechanistic chemistry associated with boron compounds employed in direct amidation for several years.12*a* The diversity of the catalytic systems reported to date, from simple boric acid^9^ or boronic acids^11^–^13^ to carefully designed cooperative catalysts,^15^ including poly-boron-based systems,^16^ poses a challenge in terms of identifying key structural motifs which are necessary for effective catalysis. Even considering boronic acid catalysts alone, bifunctional systems containing nitrogen and boron,^12^ or iodine/bromine and boron^13^ and electron deficient aryl boronic acids^11^ have all been demonstrated to be highly effective. Currently, a comprehensive understanding of how the catalyst structure affects the activity is clearly lacking.

**Scheme 2:**
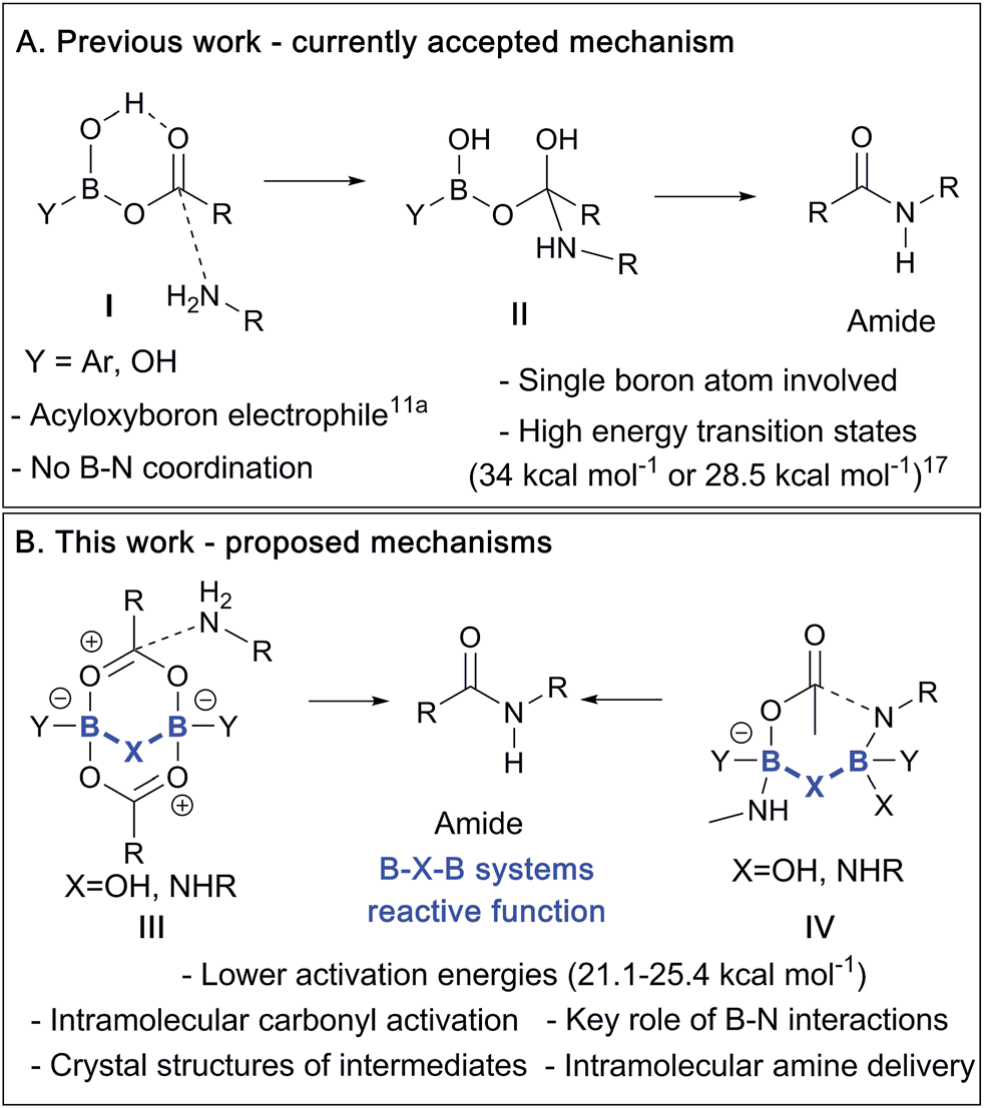
(A) Currently proposed mechanism *via* a monomeric acyloxyboron intermediate. (B) Summary of mechanisms proposed in this paper involving dimeric B–X–B motifs.

Previously, an acyloxyboron-based intermediate was proposed as the reactive catalytic species,11*a* with computational studies suggesting that it was energetically plausible (Scheme 2A).^17^ As shown in Scheme 2, this mechanism involved the formation of the acyloxyboron derivative which then had to undergo nucleophilic attack by an amine to give the amide, after loss of water. However, despite the fact that DFT calculations^17^ suggest that acyloxyboron species are feasible intermediates in direct amidation catalysis, actual definitive physical evidence of the mechanistic role of boron in such reactions is sparse and could be misleading due to the potential for numerous possible boron species to be present in such reactions.12*a* Indeed, it should be noted that acyloxyboron species are particularly prone to dimerization/oligomerization, as mentioned above with regard to B(OAc)_3_ (*vide supra* and Scheme 1).

In this work, we report recent studies in which we have probed the types of species potentially involved in boron-catalysed amidation reactions using a combination of experimental, structural and theoretical approaches. As a result, we postulate that acyloxyboron species like **I** (Scheme 2A) are unlikely to be sufficiently reactive as acylating agents, and we propose new mechanistic pathways for boron-mediated amidation which proceed through B–X–B systems, as shown in Scheme 2B. We also demonstrate their feasibility experimentally, supported by computational methods. Indeed, several routes for catalytic amidation are suggested, all of which were calculated to have lower energy barriers than the previously postulated single boron atom mechanisms.^17^ Importantly, they involve intermediates with two boron centres, and take account of the possible role of both boron–nitrogen and boron–oxygen interactions, which can be readily observed between amines and boronic or borinic acids, and between these boron compounds and carboxylic acids. These observations point to a generic structural motif which can potentially explain the success of cooperative,^15^ bifunctional12*a* and polyboron amidation catalysts,^16^ and they may even explain catalysis in other, related reactions.^18^

## Results and discussion

### Observations on the acyloxy mechanism

**Scheme 3:**
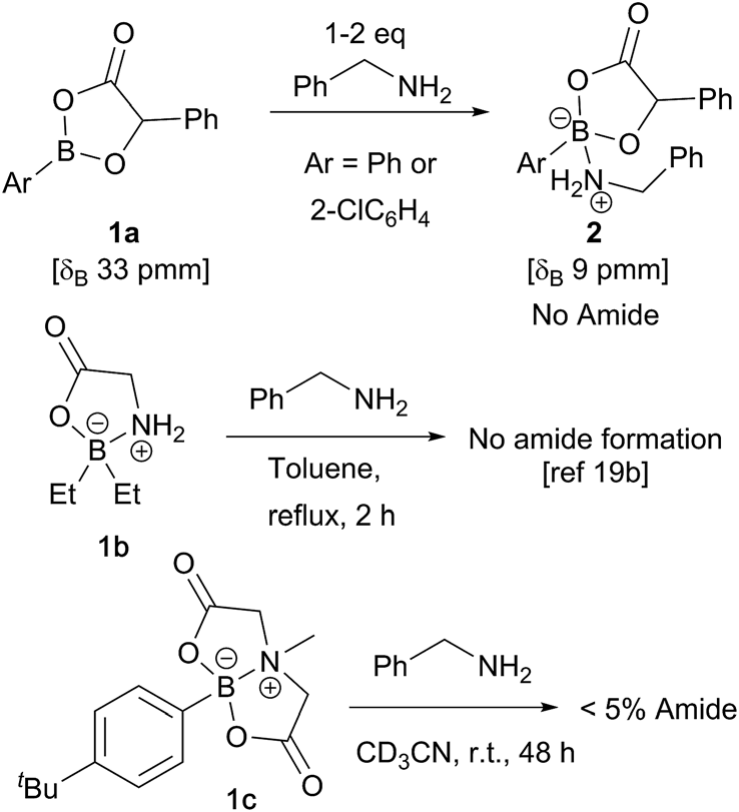
Monomeric acyloxyboron species typically show low reactivity towards amines.

In recent computational studies,^17^ both tetrahedral17*a* and trigonal17*b* acyloxyboron species were considered as possible active acylating agents, with the latter leading to a lower energy barrier for the reaction with an amine. There is little experimental evidence for these proposed intermediates,11*a* and only a few classes of well-characterised acyloxyboron compounds have been reported.^4^,^5^,^18^ It is also not clear whether acyloxyboron compounds are effective acylating agents for amines; certainly, trigonal acyloxyboron compounds are likely to be Lewis acidic, and therefore, reaction of an amine at the boron atom should have a much lower energy barrier than the alternative nucleophilic attack at the carbonyl leading to amide formation. Indeed, readily accessible acyloxyboron compound **1a**19*a* reacted cleanly with benzylamine to give the ‘ate’-complex **2** (Scheme 3). Even in the presence of excess amine, no conversion to amide was observed. An investigation of previously reported tetrahedral acyloxyboron species had suggested that they were also ineffective as acylating agents for amines. For example, borinate **1b**, generated by reaction of triethylborane with glycine, was recovered unchanged after heating at reflux in toluene with benzylamine,19*b* and preliminary work in our laboratories found that MIDA-boronate **1c** also showed no reactivity towards amines under mild conditions (others have reported related observations^20^) which clearly demonstrates that such species are not effective acylating agents. These results contrast with the mechanism examined computationally by Marcelli in which a tetrahedral acyloxyboronate was proposed to undergo attack by an amine in a reaction that typically takes place at room temperature.17*a*

### Borinic acids in direct amide formation

Analysis of carboxylic acid/amine/boronic acid reaction systems is a complicated task because multiple equilibria coexist in such reaction mixtures,12*a* leading to the presence of many different species. Hence, in order to better understand the potential catalytic role of boronic acids in direct amidation, we examined related diarylborinic acids as model systems. Interestingly, in our hands,^14^ these compounds were found to be ineffective, unreactive direct amidation catalysts, and therefore, ideal for probing the structural effects of coordination *versus* reaction with both carboxylic acids and amines.

**Scheme 4:**
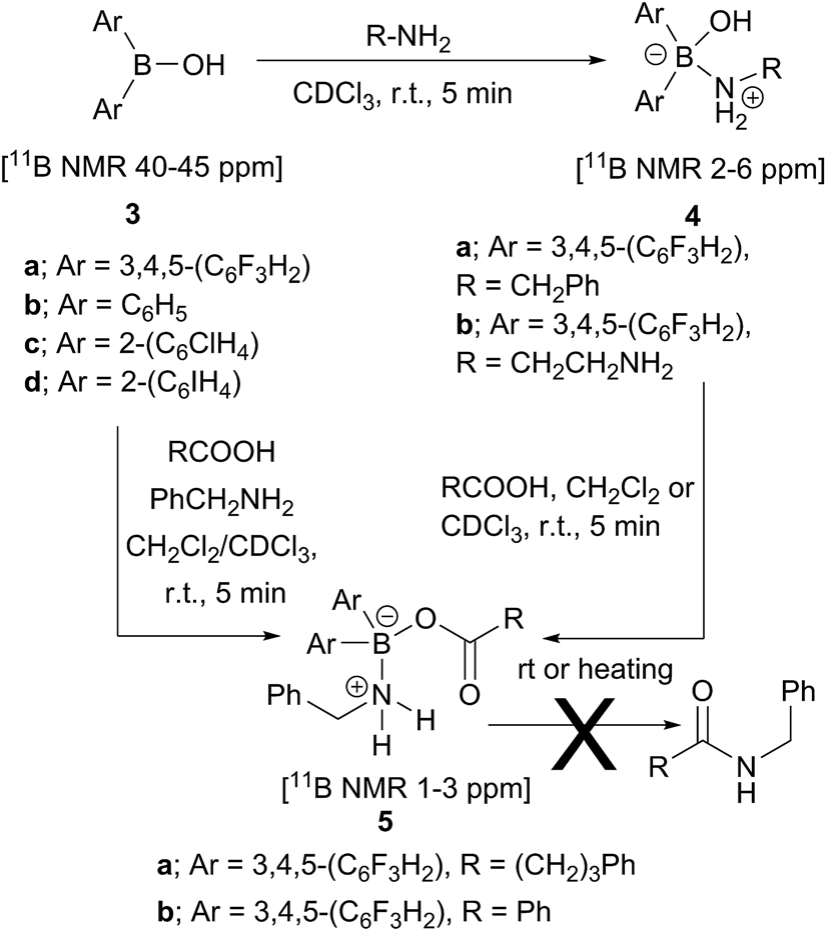
Reactions between borinic acids **3** and amines to give complexes **4**, and subsequent conversion into aminocarboxylate complexes **5**, which are not readily converted into amides.

**Fig. 1:**
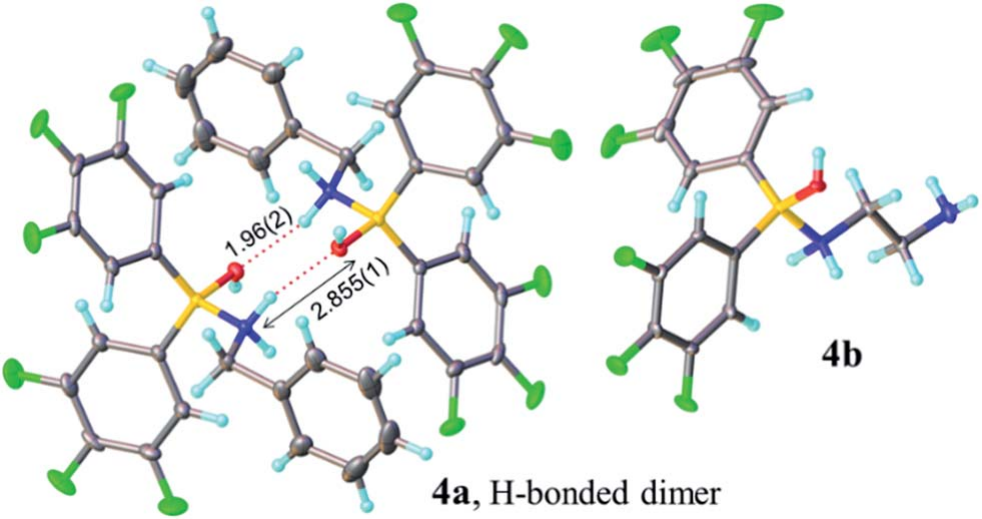
X-ray structures of Lewis adducts **4a** (H-bonded dimer) and **4b**.

Our first finding was that amines (*e.g.* benzylamine and ethylenediamine) readily coordinate to borinic acids **3a–c** (freshly obtained from their corresponding ethanolamine complexes, see ESI†) with formation of the corresponding “ate”-complexes **4** (see Scheme 4). Complexes of type **4** were observed by ^11^B NMR *via* a signal shift from *δ* 40–45 ppm for the borinic acids **3** to a peak in the “tetrahedral” region around 2–6 ppm. Importantly, this diagnostic shift was observed in all cases, and interestingly, when bis-3,4,5-trifluorophenylborinic acid **3a** was employed, the adducts **4a** and **4b** crystallised directly from the reaction mixtures allowing unambiguous structural verification by X-ray crystallography (see Fig. 1).

**Fig. 2:**
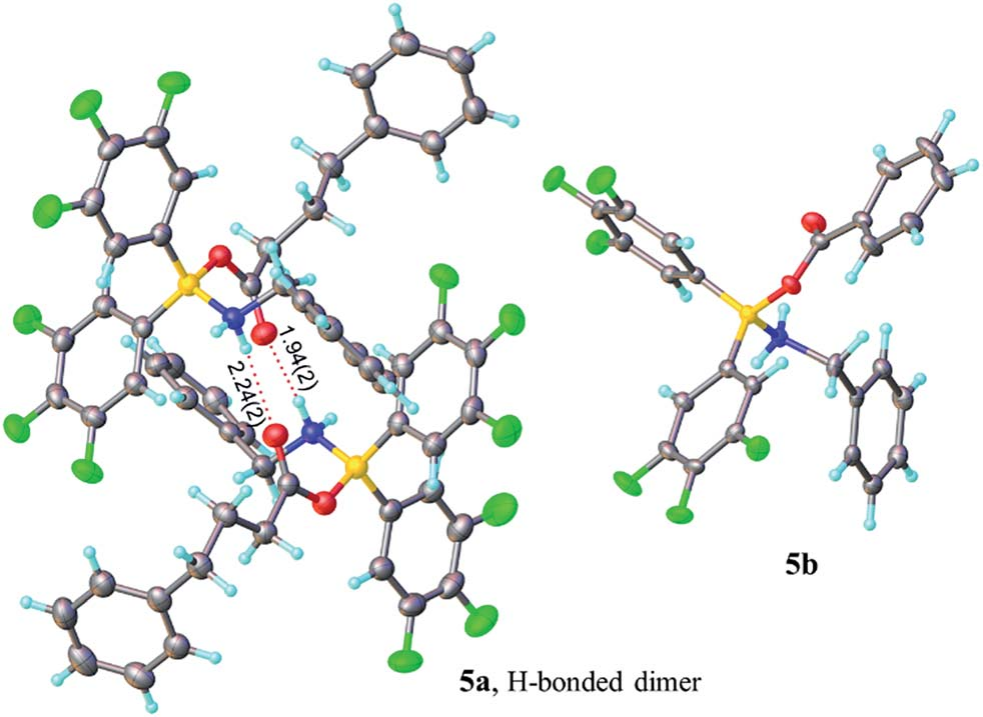
X-ray structures of amino-carboxylate borinic complexes **5a** (showing a hydrogen-bonded dimer of two independent molecules) and **5b** (disorder of phenyl ring is omitted for clarity). For **5c** and **5d** see ESI.†

Borinic acid–amine complexes **4** were found to react with carboxylic acids (*e.g.* benzoic acid and 4-phenylbutyric acid) resulting in amine–carboxylic acid complexes **5** (Scheme 4). The same complexes **5** were also formed if all three components (borinic acid, amine and carboxylic acid) were mixed together simultaneously (see ESI† for related reactions and structures from the reactions of **3b** and **3c**). In many cases, these closely related products were also unambiguously characterised by X-ray crystallography (see Fig. 2). It should be noted that complexes **5** are structurally closely related to the acyloxyboron intermediate proposed for direct amidation reactions (I, Scheme 2A), with the NH_2_ unit potentially able to hydrogen bond to the carbonyl oxygen to provide activation. Most importantly, however, complexes **5** could not be converted to amides either by heating, or by addition of further aliquots of acid, amine or base; a result which strongly corroborates our hypothesis that, in general, single-boron acyloxy compounds are not competent acylating agents for amines.

**Fig. 3:**
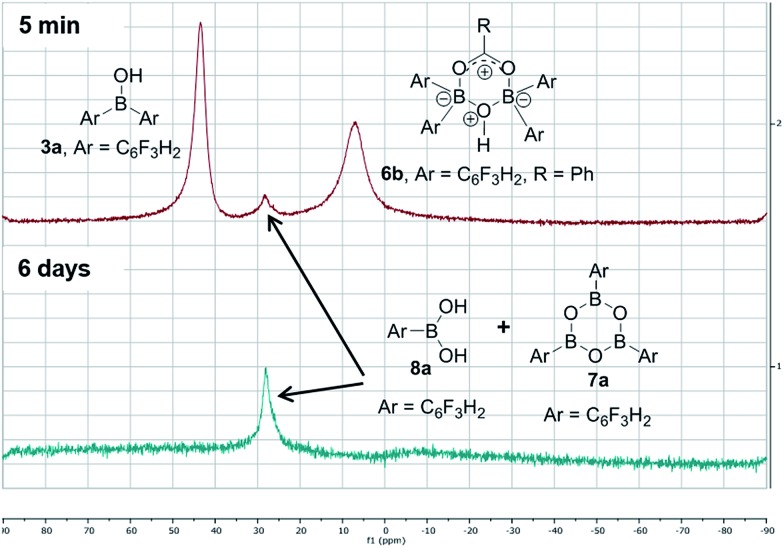
^11^B NMRs of a mixture of bis-3,4,5-trifluorophenylborinic acid **3a** with benzoic acid after 5 min and after 6 days upon mixing (Ar = 3,4,5-trifluorophenyl).

**Scheme 5:**
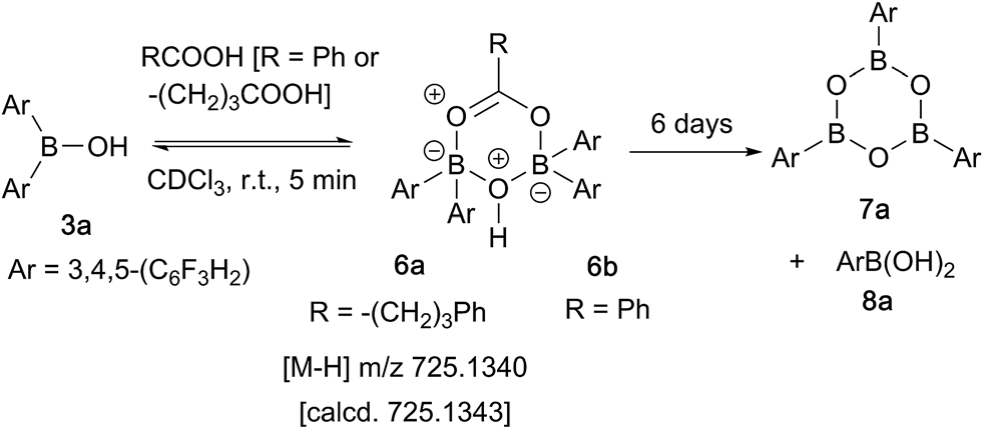
Reaction of 3,4,5-trifluorophenylborinic acid **3a** with 4-phenylbutyric or benzoic acid, resulting in boroxine formation.

**Fig. 4:**
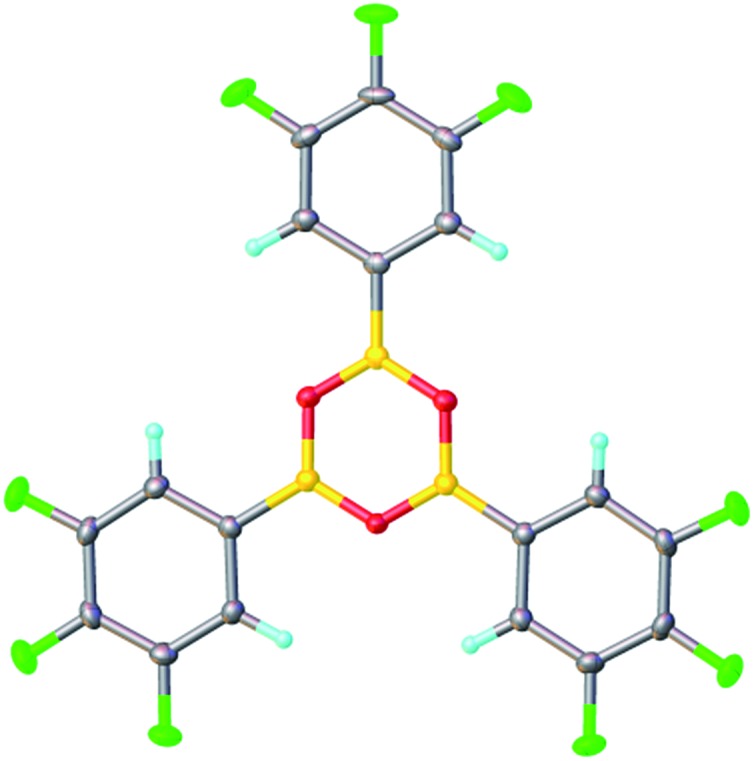
X-ray structure of 3,4,5-trifluorophenylboroxine **7a** in orthorhombic β-form at 120 K.

In contrast, when bis-3,4,5-trifluorophenylborinic acid **3a** was reacted with carboxylic acids alone, immediate formation of a boron “ate”-complex was observed according to ^11^B NMR (Fig. 3), as shown in Scheme 5, and a subsequent slow protodeboronation reaction occurred which generated a boronic species (see Fig. 3). This was followed, in this case, by slow crystallisation of the corresponding boroxine **7a** (Fig. 4). Hence, we propose that the “ate”-complex observed by ^11^B NMR was **6** (Scheme 5 and Fig. 3) based on systems of this type which have been isolated.^21^ In addition, the mass spectrum of the mixture showed an *m*/*z* 725.1340 (calcd *m*/*z* for **6a** [M – H] 725.1343). Complex **6a** underwent protodeboronation–dehydration to give the notably uncomplexed boroxine **7a**, and its corresponding boronic acid **8a**. The fact that such reactions occur readily at room temperature shows that formation of B–O–B species is extremely facile. Both borinic and boronic acids can thus readily form bridged B–O–B systems such as **6** and **7** (and **11**, *vide infra*) which demonstrates that such motifs play an important role, both in boron chemistry in general, and likely also in direct amidation catalysis. It also exemplifies the ease with which a borinic acid undergoes protodeboronation in the presence of a carboxylic acid.

Having observed that borinic acids do not mediate amide bond formation, we were surprised by a recent report describing borinic acid-catalysed amide formation.^14^ However, when we attempted amidations under exactly the same conditions to those recently reported,^14^ amides could indeed be obtained in good yield; an explanation for which was required.

**Scheme 6:**
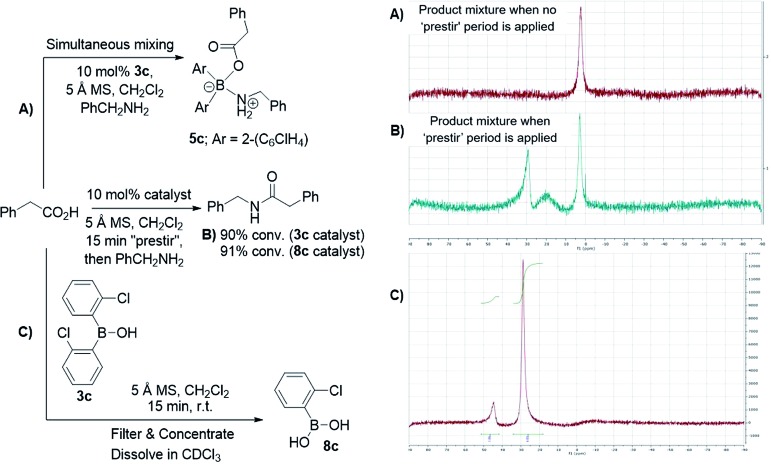
Examining bis-2-chlorophenylborinic acid **3c** as a catalyst for direct amide formation and ^11^B NMR spectra of product mixtures obtained in 3 examined cases.

After further investigations, we discovered that the key to catalytic reactivity was the 15 minute “prestir” period, as explained in Scheme 6. When the claimed catalyst, bis-2-chlorophenylborinic acid **3c**, was exposed to a carboxylic acid in the presence of activated 5 Å molecular sieves (MS), catalytic activity was observed. Without this “prestir” period, no amide formation occurred. The significant finding was that the ^11^B and ^1^H NMR spectra of the crude reaction mixtures were different depending on whether the “prestir” period was applied or not (Scheme 6A and B). This data indicated that if borinic acid **3c**, phenylacetic acid and benzylamine were mixed simultaneously without the “prestir” period, aminocarboxylate complex **5c** was formed and remained stable over at least one week in solution (*vide supra*, Scheme 4 and 6A). When analyzing the product mixtures obtained after the “prestir” period, significant amounts of boronic species were observed (Scheme 6B). We propose, therefore, that borinic acid **3c** acts as a pre-catalyst, and its protodeboronation occurs rapidly during the “prestir” period to give 2-chlorophenylboronic acid **8c**, which is well known to be an effective catalyst for direct amidation, as reported by Hall *et al.*13*a* This finding was further verified by a separate experiment in which bis-2-chlorophenylborinic acid **3c** and phenylacetic acid were stirred for 15 minutes with 5 Å MS, leading to a 1 : 6 ratio of borinic to boronic species (Scheme 6C). Interestingly, the process of protodeboronation occurred much more slowly when activated 4 Å MS were used compared to 5 Å MS, so the particular sieves used play an important role in assisting the deboronation reaction (it is worth noting that generally either 3 or 4 Å MS are used for achieving dehydration in most amidation reactions as an alternative to azeotropic water removal. See ref. 13b for a relevant discussion).

To further support the fact that boronic acid **8c** is actually formed from borinic acid **3c***in situ* under the “prestir” conditions and that **8c** is the active catalyst, we compared **3c** and commercial 2-chlorophenylboronic acid **8c** side-by-side under the same conditions (Scheme 6B). Similar amide conversions were observed in the two reactions with **3c** and **8c** (91% and 90% respectively), which supports our proposal that the boronic acid is the actual catalyst. It can, therefore, be concluded that borinic acids do not catalyse amidations directly, and can only do so through protodeboronation to the boronic acid.

The fact that aminocarboxylate complexes **5** could not be converted into amides seems to be rather inconsistent with the commonly accepted mechanism for boron-mediated amidation (Scheme 2A). Since borinic acids are stronger Lewis acids than boronic acids, it might be expected that acyloxyborinic species **5** should actually be stronger acylating agents than the acyloxyboronic analogues, and these complexes also have the ability to form internal hydrogen bonds between the protonated amine and the carbonyl. Since both boronic acids and boric acid catalyse direct amidation,^8^ but borinic acids do not, it implies that at least three free coordination sites are needed on the boron atom in order for amidation catalysis to be possible. This conclusion led us to consider, therefore, whether B–X–B dimer formation might be essential for the observed catalytic activity, and that a bridging X-group (*i.e.* O or N) could be essential to orchestrate the catalytic process. We therefore looked more closely at the speciation that might be occurring in boronic acid-mediated direct amidation catalysis, using both experimental and theoretical methods.

### Boronic acids in direct amide formation

**Fig. 5:**
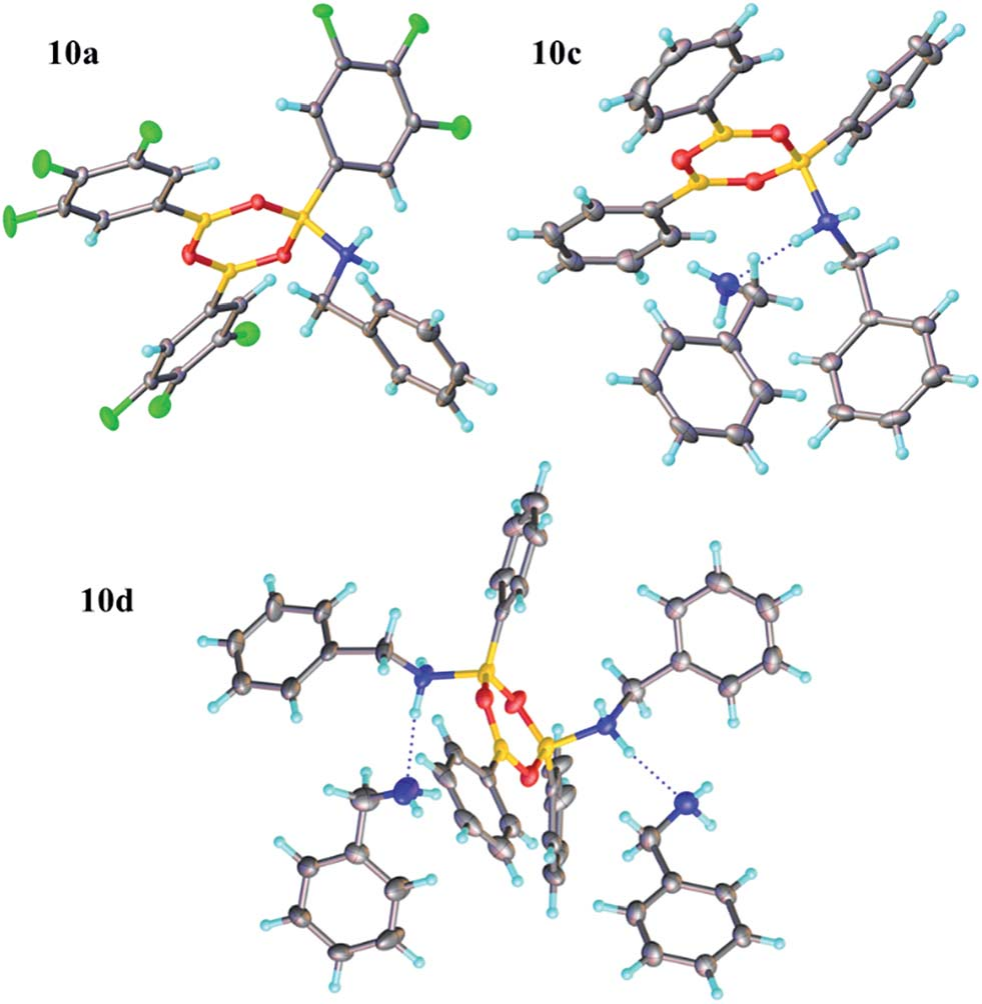
X-ray structures of 3,4,5-trifluorophenylboroxine-benzylamine adduct **10a** and phenylboronic acid derived boroxine–amine adducts **10c** and **10d**.

**Scheme 7:**
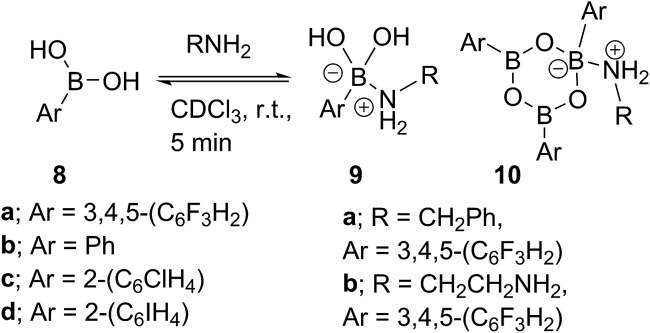
Reactions between boronic acids **8a–d** and amines at r.t.

**Scheme 8:**
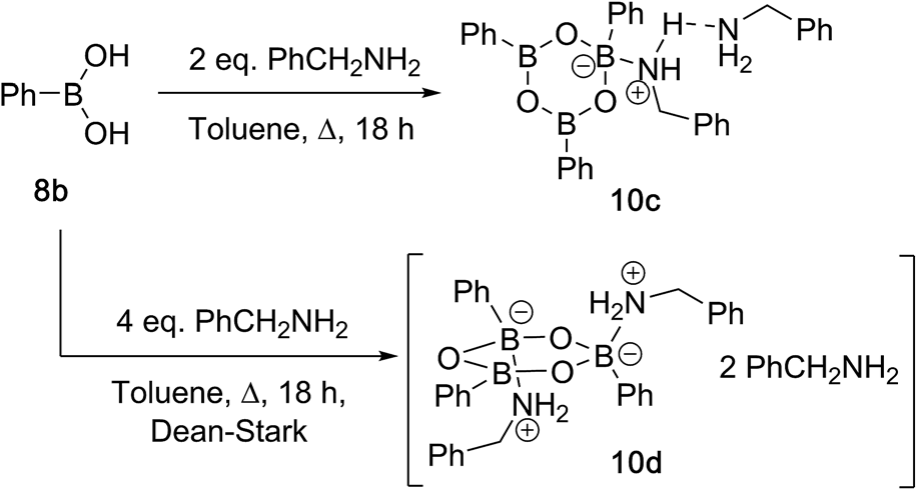
Reactions between phenylboronic acid **8a** and benzylamine at reflux.

Due to the complexity of carboxylic acid/amine/boronic acid systems, the reactivity of boronic acids with amines and carboxylic acids was initially explored separately. When treated with an amine, boronic acids appear to form complexes of general structures **9** and **10**.^20^ 3,4,5-Trifluorophenyl boroxine–benzylamine complex **10a** (Fig. 5) and the adduct with ethylenediamine **10b** (see ESI† for X-ray) were formed at room temperature and crystallised (Scheme 7), while phenylboroxine complexes **10c** and **10d** with two or four benzylamine molecules (Scheme 8 and Fig. 5) formed upon reaction of **8a** with two equivalents of benzylamine at reflux in toluene (either with reflux condenser or with Dean–Stark water removal apparatus). An important observation was that the amine promotes dissolution of the boronic acid in organic solvent by formation of these boroxine complexes (see ESI†).

These examples further underlined (also observed for borinic acids, *vide supra*) the importance of B–N interactions, which should readily occur between the amine and any trigonal boron species present in catalytic amidation systems. This is also consistent with our hypothesis that any trigonal acyloxyboron species is likely to react at the boron atom with an amine and indeed, such interactions may facilitate interconversion to and from boroxine species.

**Fig. 6:**
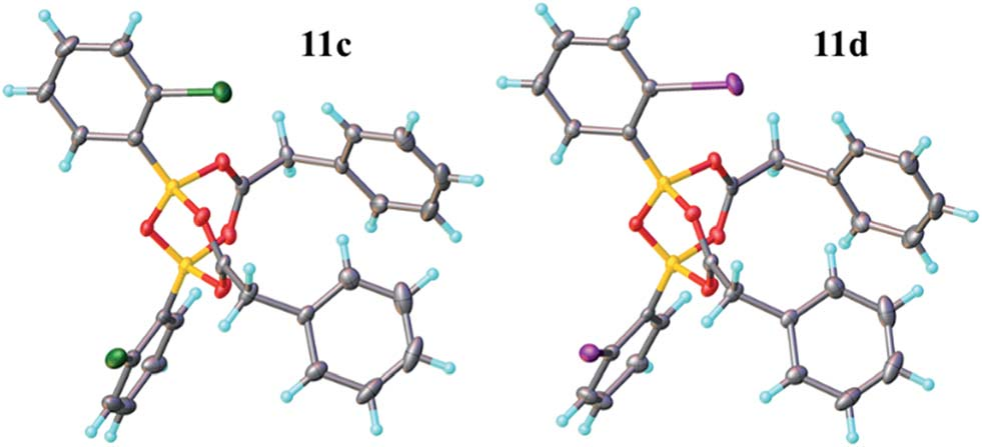
X-ray structures of B–O–B “ate”-complexes **11c** and **d**.

**Fig. 7:**
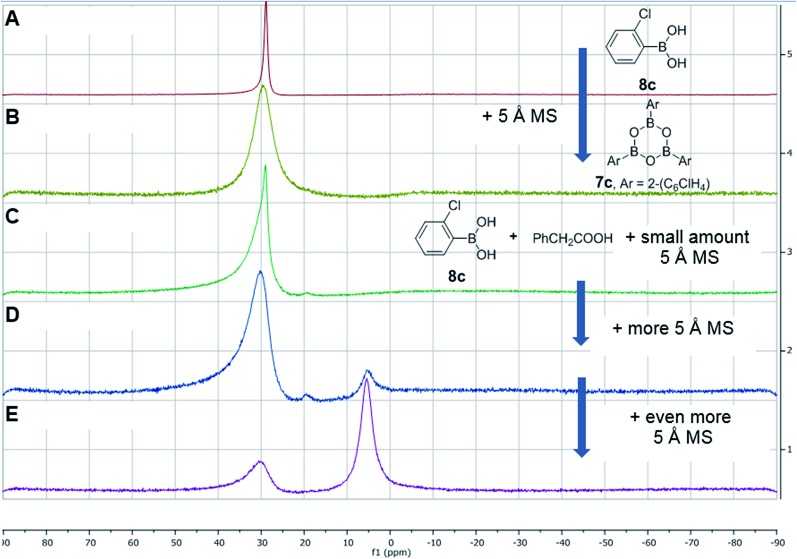
^11^B NMR spectra showing interactions between 2-chlorophenylboronic acid **8c**, phenylacetic acid and 5 Å MS. (A) 2-Chlorophenylboronic acid; (B) addition of 5 Å MS to (A), leading to boroxine formation; (C) addition of a tiny amount of 5 Å MS to a mixture of boronic and carboxylic acid; (D) addition of more 5 Å MS; (E) addition of even more 5 Å MS.

Having investigated the various interactions between amines and boronic acids, we next examined reactions between carboxylic acids and boronic acids. It was observed that boronic acid **8c** did not react with phenylacetic acid unless 5 Å MS were introduced into the reaction mixture. In the presence of 5 Å MS alone, boronic acid **8c** was converted into the corresponding boroxine **7c** (Fig. 7A and B). With carboxylic acid present, initial formation of boroxine **7c** occurred (Fig. 7C), and as more MS were added as well as phenylacetic acid, a new “ate”-complex (*δ* 5 ppm, Fig. 7D and E) was formed, and the equilibrium between it and boroxine **7c** was pushed further towards the new species. Careful crystallisation from this heterogeneous system (containing solid 5 Å MS), allowed crystals to be separated which were suitable for X-ray characterisation. The product was unambiguously identified as the “ate”-complex **11c** (see Fig. 6) which is structurally related to analogous complexes reported elsewhere.^22^ Interestingly, in a separate NMR reaction, addition of an excess of phenylacetic acid to boronic acid **8c**, in the presence of 5 Å MS shifted the equilibrium even further towards the “ate”-complex as seen by ^11^B NMR (see further examples in the ESI†).

The amount of MS used also had a profound effect, and with a large excess of MS it was possible to generate >85% of the “ate”-complex in the mixture (calculated by ^11^B NMR, see Fig. 7E and ESI† for further examples). The resulting species **11c** is analogous to “ate”-complexes **6** in the borinic acid series, which undergo subsequent protodeboronation to give boronic acid derivatives rather than act as amidation catalysts. This again highlights the need for the correct boron oxidation state, so that there are sufficient free coordination sites on the boron centre: borinic acids being unreactive but boronic acids/boric acid being catalytically active.

It should be noted that without 5 Å MS the formation of the diacyloxy B–O–B bridged complexes **11** did not occur to a significant extent; even when 4 Å MS were used, less than 5% of the diacyloxy B–O–B complex **11c** was observed. Interestingly though, a similar diacyloxy B–O–B bridged complex **11d** could be isolated by reaction of 2-iodophenylboronic acid **8d** and phenylacetic acid. In contrast, when boronic acids without an *ortho*-substituent, such as 3,4,5-trifluorophenylboronic acid **8a**, were reacted with phenylacetic acid, the “ate”-complex was formed, as shown by ^11^B NMR, but did not crystallise and only boroxine **7a** crystallised from the reaction mixture. In the case of *ortho*-substituted boronic acids, the boroxines are likely to be destabilized due to steric effects, and thus, the formation of dimeric “ate”-complexes **11** may be more favourable, enabling the formation of catalytically competent species.

**Scheme 9:**
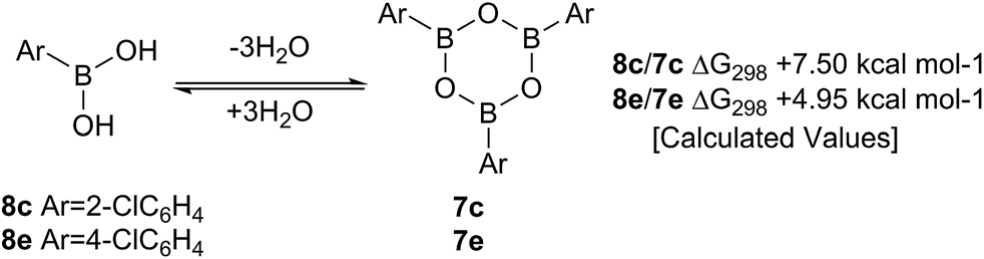
Calculated Δ*G*_298_ values for the formation of boroxines from *o*-chlorophenylboronic **8c** acid and *p*-chlorophenylboronic acid **8e**.

Such sterically-induced destabilization of boroxines was indeed confirmed by relative free energy calculations^29^ (B3LYP+D3/Def2-TZVPP/solvent = dichloromethane) in which cyclisation of *o*-chlorophenyl boronic acid **8c** to give **7c** + 3H_2_O was found to be 2.55 kcal mol^–1^ more endoenergic than formation of the boroxine **7e** from *p*-chlorophenyl boronic acid **8e** (Scheme 9). For the former, this is due to reduced conjugation between the aryl rings and the boroxine ring as a result of twisting of ∼11°. At 298 K, this translates into a ∼10^5^-fold decrease in the equilibrium concentration of the boroxine **7**. Indeed, the high catalytic activity of such *ortho*-substituted systems may be due to these key reactivity effects which can destabilise the boroxine, a species which is likely to be an off-cycle resting state of the catalyst.

Interestingly, when we attempted to reproduce the reported^10^ generation of an “acyloxy” boron derivative as described by Ishihara *et al.* using 3,4,5-trifluorophenylboronic acid **8a** heated at reflux with phenylacetic acid in toluene (Dean–Stark water removal), there was no obvious reaction by either ^11^B and ^1^H NMR or ReactIR.

**Fig. 8:**
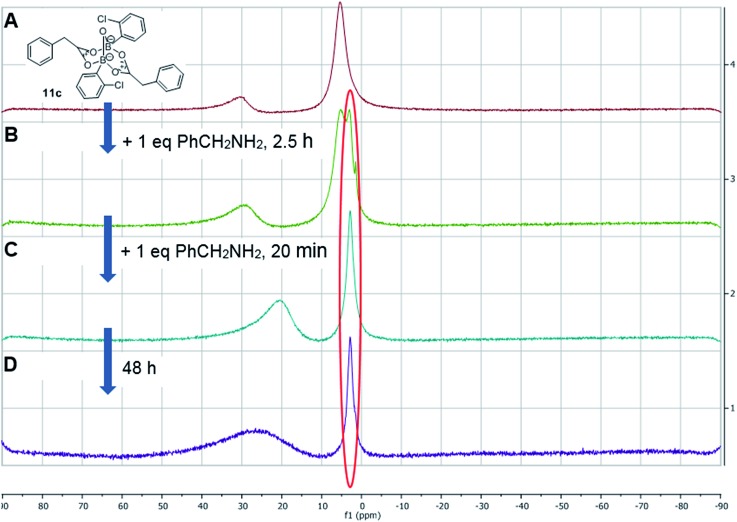
^11^B NMRs of (A) mixture of 2-chlorophenylboronic acid **8c** and phenylacetic acid in the presence of 5 Å MS resulting in equilibrium between B–O–B dicarboxylate **11c** (*δ* 5 ppm) and boronic species **7c** (*δ* 30 ppm); (B) addition of 1 equivalent (per “ate”-complex **11c**) of benzylamine, 2.5 h, showing formation of a new complex **12**; (C) addition of 2^nd^ equivalent of benzylamine, 20 min; (D) same mixture over 48 h.

Having isolated the diacyloxy B–O–B complexes **11**, we attempted to react these systems with benzylamine (Fig. 8). Surprisingly, when 1 equivalent of amine per dicarboxylate complex **11c** was added, the ^11^B NMR showed the appearance of a new species **12** with a signal at 3 ppm, but ^1^H NMR showed no amide formation even after 2.5 h (see ESI†). However, when a second equivalent of amine was added, amide formation was seen within 20 minutes. This reaction of the diacyloxy B–O–B bridged complex **11c** with two equivalents of benzylamine did not reach completion, and the signals we assign to the new species **12** remained in the ^1^H NMR even after 48 h. It is possible that this is due to the fact that there is an excess of boron compound (in total) with respect to amine in the reaction mixture, which indicates that an excess of amine and/or carboxylic acid per catalyst molecule is required for catalytic turnover and efficient reactivity.

**Scheme 10:**
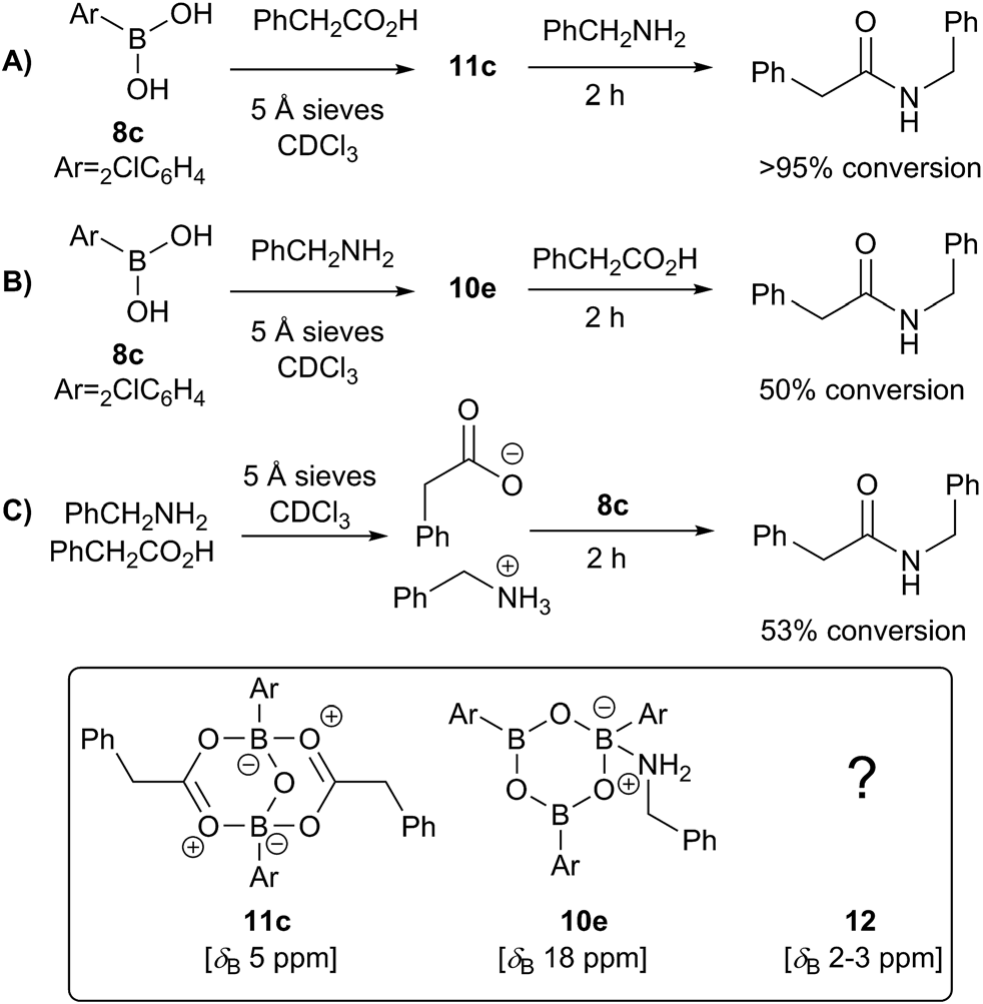
Observed boron-containing species in catalytic amidation reactions containing boronic acid **8c**, carboxylic acid and amine.

We then went on to investigate the species present in catalytic reaction mixtures containing boronic acid, amine and carboxylic acid (Scheme 10). Interestingly, the behavior of these systems was dependent on the order of reagent addition. When the carboxylic acid was premixed with boronic acid **8c** in the presence of 5 Å molecular sieves, rapid formation of the “ate”-complex **11c** was observed (Scheme 10A); subsequent addition of amine led to complete formation of amide within 2 h. During the amidation reaction, small quantities of boroxine–amine complex **10e** were also observed, along with a signal consistent with the unknown species **12** (*δ*_B_ 3 ppm). The amine complex **10e** became the dominant species after the amidation was complete. In contrast, when boronic acid **8c** was mixed with the amine in the presence of 5 Å molecular sieves (Scheme 10B), rapid formation of a boroxine amine complex (*e.g.***10e**) was observed, though a lower ^11^B NMR shift of 15 ppm suggests that **10e** was likely in equilibrium with a compound analogous to **10d**, in which two amine molecules are coordinated to the boroxine. Addition of carboxylic acid led to amide formation, but after 2 h the reaction had only reached 50% conversion. Again, during the amidation reaction both **10d** and **12** were observed, with the latter being the major species. Finally, a reaction was carried out in which amine and carboxylic acid were pre-mixed to give the ammonium carboxylate salt and boronic acid **8c** was then added (Fig. 10C). This exhibited similar behavior to the reaction shown in Fig. 10B, giving 53% conversion to amide after 2 h.

Finally, when either B_2_O_3_ or boric acid were mixed with benzylamine and phenylacetic acid (both insoluble in CDCl_3_), some dissolution was observed and the resulting ^11^B NMR showed the appearance of an “ate”-complex (see ESI†), suggesting that these boron compounds can be pulled into solution *via* complexation.

### Summary of observations

**Scheme 11:**
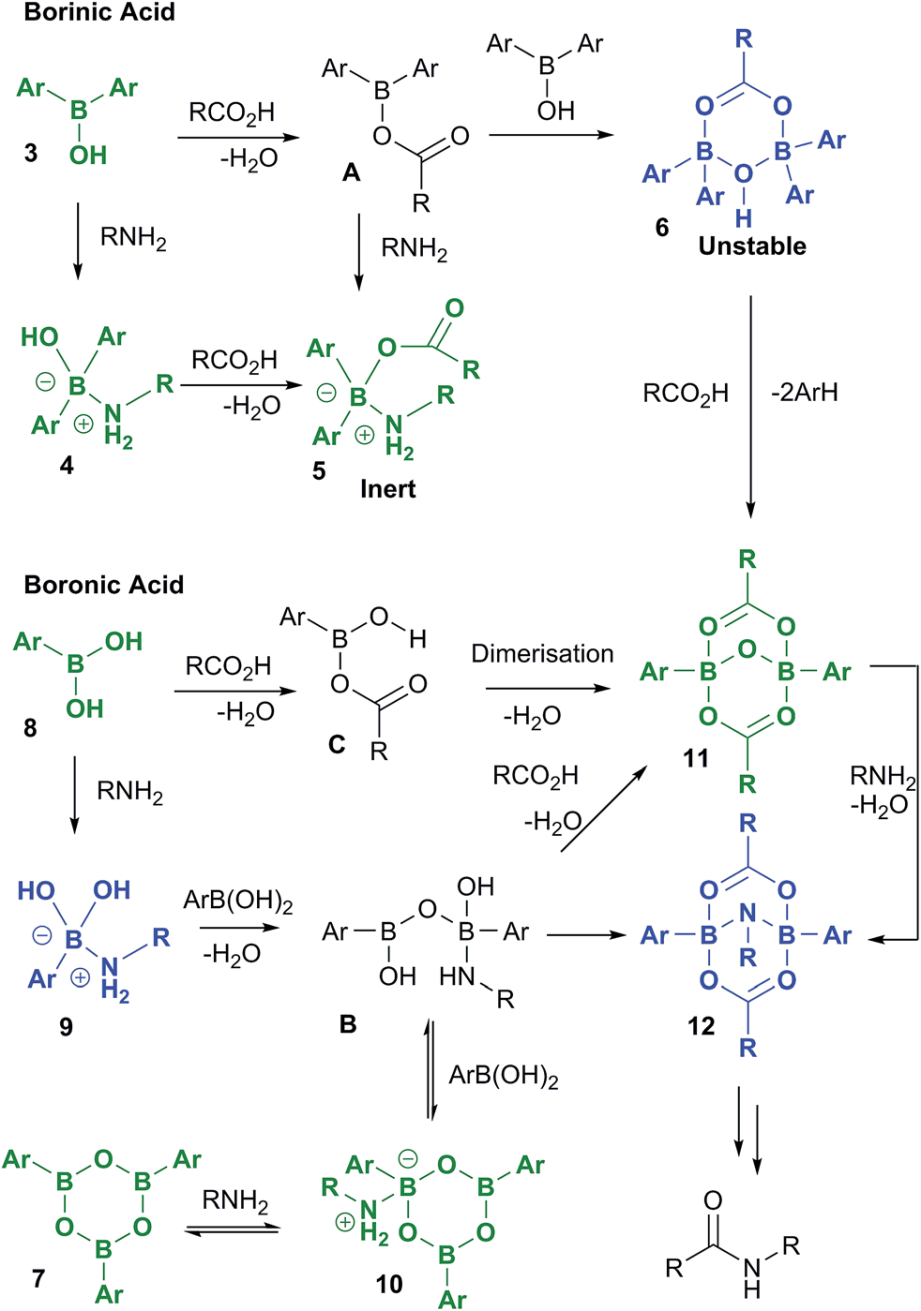
Boron species potentially generated from reaction of both borinic acids and boronic acids with carboxylic acids and amines. Compounds which have been fully characterized in this study by X-ray crystallography are shown in green, and those which have been observed by NMR and/or mass spectrometry are shown in blue. Proposed intermediates during the formation of the observed boron species are shown in black.

A full summary of possible intermediates generated from borinic/boronic acids by reaction with carboxylic acids and amines is shown in Scheme 11, along with likely pathways for their formation. Thus, borinic acids **3** form inert complexes **5** (*via***4** or postulated intermediate **A**) in the presence of amine and carboxylic acid, but in the presence of carboxylic acid alone, they can undergo protodeboronation (probably *via* observed intermediate **6**), to enter the boronic acid cycle. Boronic acids **8** undergo rapid complexation with amines to give intermediates of type **9**, which are rapidly converted through to boroxine–amine complexes **10***via* postulated intermediate **B**, facilitating dissolution of the boronic acid in organic solvents. Direct condensation of a boronic acid **8** with a carboxylic acid should lead initially to **C**, the previously proposed reactive acylating agent.11*a* However, as we have shown above, **C** is likely to undergo self-condensation under the dehydrating conditions to give “ate”-complexes of type **11** in a similar fashion to the dimerization of B(OAc)_3_ (*vide supra*, Scheme 1). This latter complex could also potentially be formed from **B**, through condensation with two molecules of carboxylic acid. We tentatively propose that intermediate **12** may be the amine-bridged dimeric “ate”-complex shown, which is closely related to **11**. This is based upon the fact that **12** is formed by reaction of **11** with amine under dehydrating conditions, and the fact that the NMR signals for **12** are similar in line shape to those for complex **5** (see ESI†). Under catalytic conditions, a similar boron NMR peak is observed that may be **12**, or a related compound derived from it.

Overall, these results lead to the conclusion that formation of complexes from reaction of boron centres with both carboxylic acids and amines (*e.g.***5** and **12**) is not only observed at all three investigated boron oxidation levels (borinic, boronic and boric) but is also indicative that these types of species are likely to be generated in catalytic amidation reactions. It is also clear that B–O–B linkages and B–N interactions are vitally important, and likely to be present in all catalytically active boron-based systems, *i.e.* particularly boronic acids, and likely boric acid by extrapolation. As it is not possible to be certain what other types of B–X–B systems can potentially form, to more deeply understand the role of cooperativity resulting in direct amidation catalytic activity between two boron atoms *via* a B–X–B system, we probed a number of putative mechanistic pathways using quantum mechanical modelling, all of which involve dimeric species potentially derived from **11** and/or **12**.

### Mechanistic insights and theoretical calculations

**Fig. 9:**
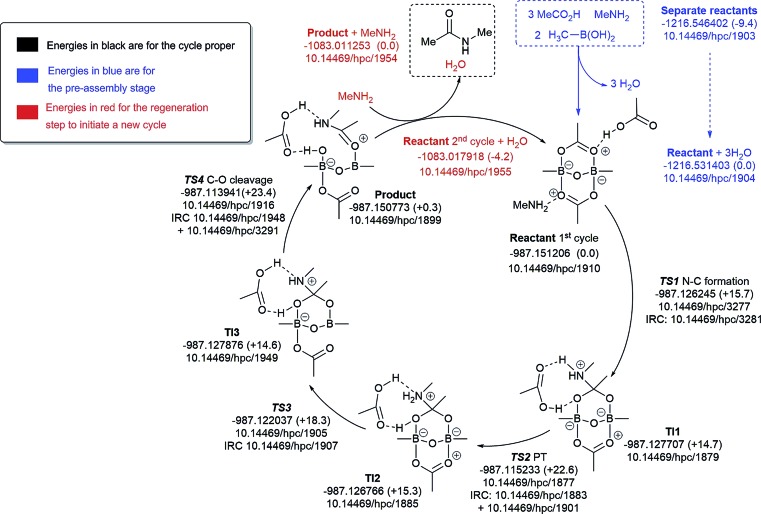
Catalytic cycle involving one amine (data^29^ sub-collection DOI: ; 10.14469/hpc/1854). The total computed free energy for each stationary point is shown in Hartree, with the relative energies shown in parentheses in kcal mol^–1^. The DOI for data repository entries for individual species are shown in the form *e.g.*; 10.14469/hpc/1885. Energies in blue are for the pre-assembly stage, in black for the cycle proper and in red for the regeneration step to initiate a new cycle. All DOIs in this figure are available as clickable links (final paginated PDF only).

**Fig. 10:**
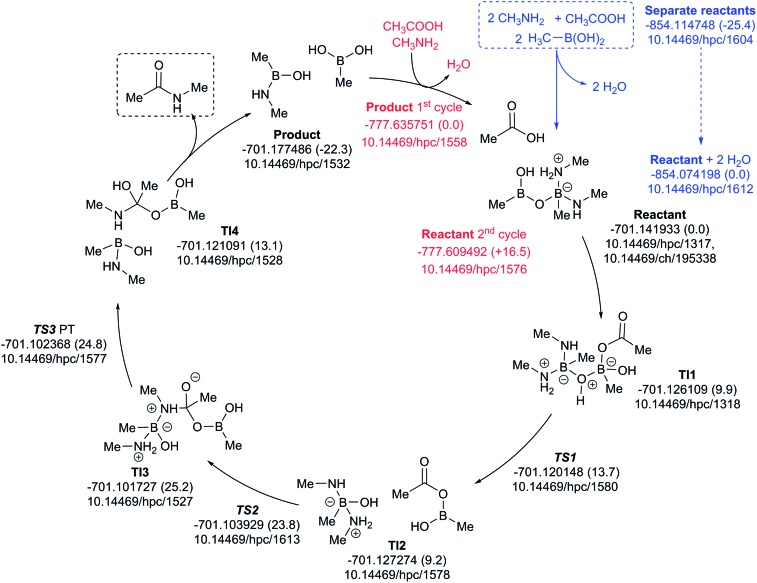
Catalytic cycle involving two amines (data^29^ sub-collection DOI: ; 10.14469/hpc/1622; the labels have the same meaning as in Fig. 9). All DOIs in this figure are available as clickable links (final paginated PDF only).

**Fig. 11:**
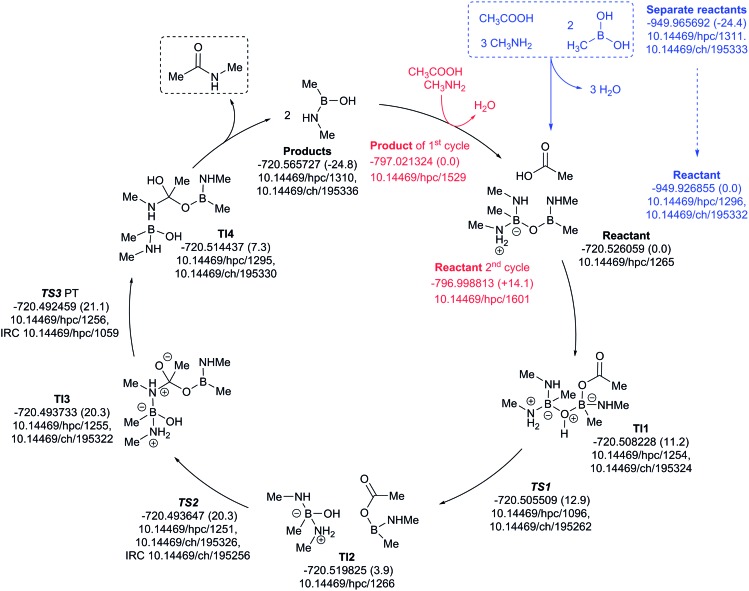
Catalytic cycle involving three amines (data^29^ sub-collection DOI: ; 10.14469/hpc/1621; the labels have the same meaning as in Fig. 9). All DOIs in this figure are available as clickable links (final paginated PDF only).

**Fig. 12:**
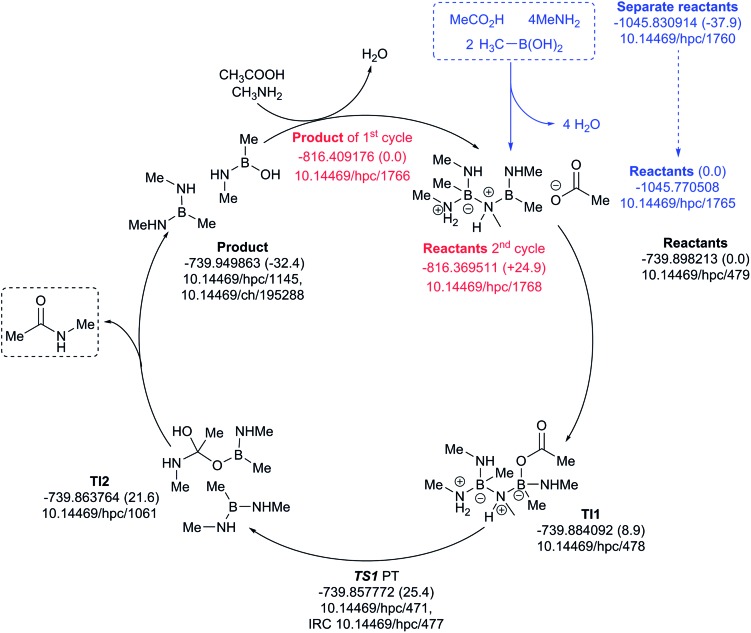
Catalytic cycle involving four amines (data^29^ sub-collection DOI: ; 10.14469/hpc/1728; the labels have the same meaning as in Fig. 9). All DOIs in this figure are available as clickable links (final paginated PDF only).

**Fig. 13:**
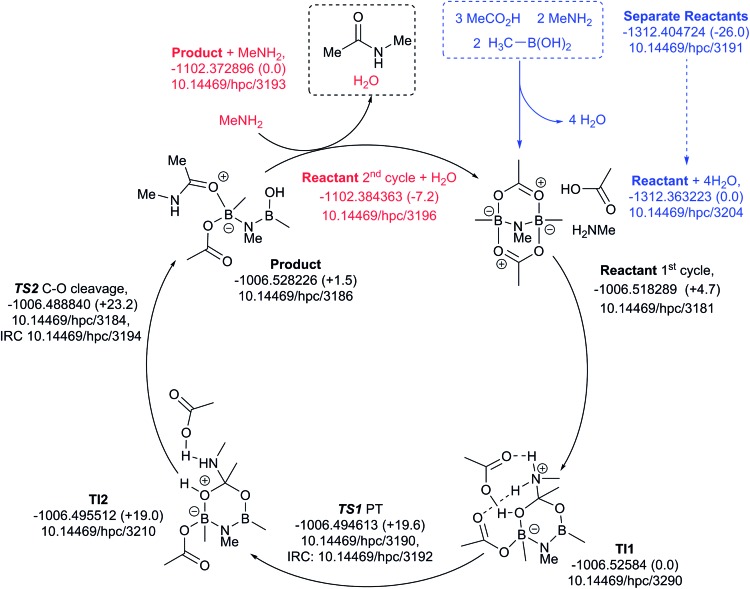
Catalytic cycle involving B–N–N bridging group (data^29^ sub-collection DOI: ; 10.14469/hpc/3188; the labels have the same meaning as in Fig. 9). All DOIs in this figure are available as clickable links (final paginated PDF only).

**Fig. 14:**
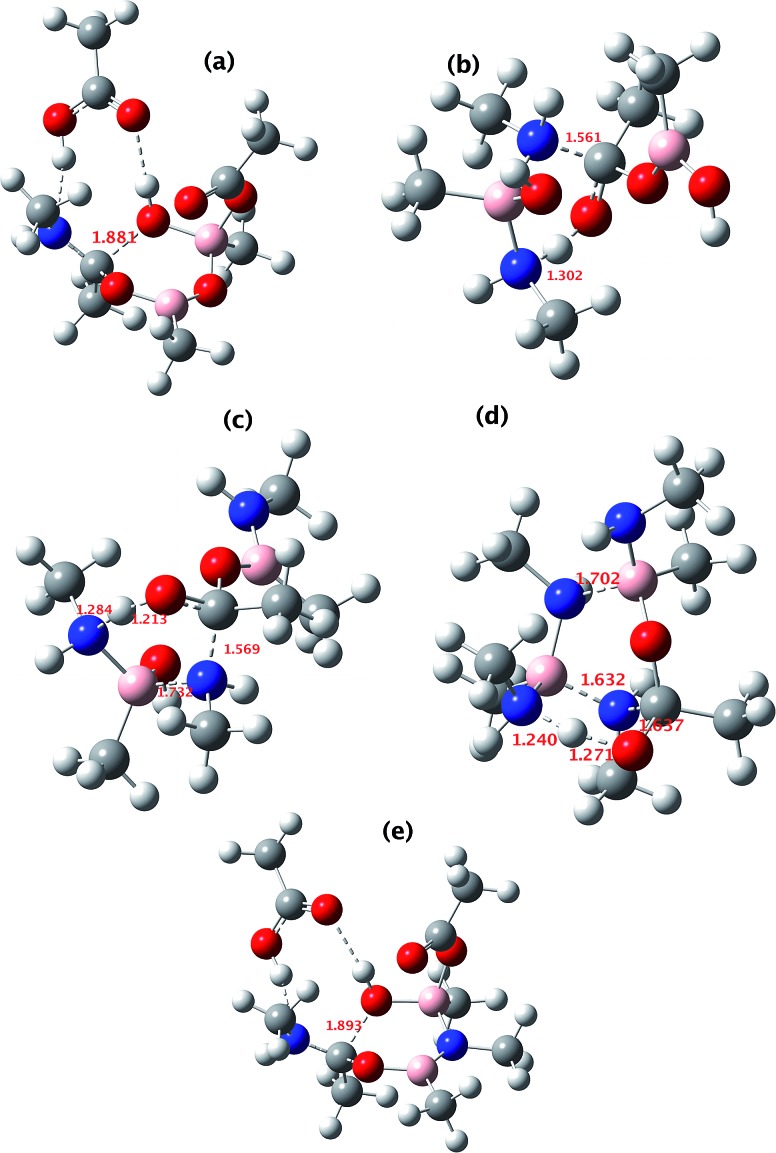
The computed structure for: (a) TS3 shown in Fig. 9; (b) TS3 shown in Fig. 10; (c) TS3 shown in Fig. 11; (d) TS1 shown in Fig. 12 and (e) TS2 shown in Fig. 13. Indicated bond length are in Å. The activation free energies Δ*G*_298_ for the five transition states are respectively 23.4, 25.2, 21.1, 25.4 and 23.2 kcal mol^–1^.

We decided to formulate different diboron systems, linked by oxygens or nitrogens and with different complexes, including amine and carboxylate and their corresponding salts, in order to have a better chance of locating the types of systems that might be responsible for direct amidation catalysis. Preliminary exploration of transition states involving monomeric B–N species resulted in high overall energy barriers (>30.0 kcal mol^–1^). Hence, pathfinder explorations of the potential energy surfaces were then carried out for the different putative B–X–B-mediated catalytic cycles, organised around five possible routes based on the number of amines involved (Fig. 9–13), in order to explore the mechanistic diversity of these systems. Key transition state structures in each cycle are shown together in Fig. 14. To enable a high quality basis set to be employed (Def2-TZVPP), substituents were all modelled at this stage using methyl groups. Each cycle included the reaction free energies of the pre-step involving species used to assemble the reactant used as the start point in the cycle, and the energies of all subsequent steps in the cycle, ending in the final products. A final reaction free energy is included to re-generate the starting reactant ready for a second catalytic cycle.

The cycle involving one amine is illustrated in Fig. 9. The pre-assembly condensation step (blue) indicates a modestly endoenergic step (ΔΔ*G*_298_ +9.4 kcal mol^–1^) producing water, which agrees with the need for water removal using molecular sieves for the reaction to proceed. With the reactant in the cycle set to a relative energy of 0.0, the high point is reached with C–O cleavage (TS3) corresponding to Δ*G*_298_ +23.4 kcal mol^–1^, corresponding to a relatively slow ambient temperature reaction. The product in this cycle is +0.3 kcal mol^–1^ relative to the reactant, but –4.2 kcal mol^–1^ is recovered in the next regeneration step (red). Such a computational exploration can only demonstrate the viability of a single pathway to product; it does not preclude, of course, that lower energy pathways might not be discovered upon a more complete exploration of the potential energy surface. These variants might include lower energy conformations of the various species explored. Rather more certain is that the free energy barrier surmounted during the cycle is probably an upper calculated bound to any true value. It should also be noted that the model used for the substituents (methyl) may introduce further uncertainty, but since the purpose here was a broad exploration of various mechanistic alternatives, we did not undertake the much more computationally resource-intensive recalculation using larger substituents.

A cycle involving two amine species is illustrated in Fig. 10. The pre-assembly step (blue) is rather more endoenergic (Δ*G*_298_ +25.4 kcal mol^–1^) than the previous cycle, but the energetic maximum in the cycle (TS3, Δ*G*_298_ +24.8 kcal mol^–1^) relative to the assembled reactants is very similar. The product of the first cycle is exoenergic by Δ*G*_298_ 22.3 kcal mol^–1^, offset in part by the regeneration step for the second cycle being Δ*G*_298_ +16.5 endoenergic.

The three-amine cycle (Fig. 11) again shows similar overall behavior. The condensation reaction resulting in the initial cycle reactant is endoenergic by Δ*G*_298_ +24.4 kcal mol^–1^, matched by the exoenergic products (Δ*G*_298_ –24.8 kcal mol^–1^), and with a regenerative step for the start of the second cycle being endoenergic by Δ*G*_298_ +14.1 kcal mol^–1^. The rate limiting TS3 has Δ*G*_298_ 21.1 kcal mol^–1^, slightly more favourable than the previous two cycles.

The four-amine cycle (Fig. 12) has a highly endoenergic condensation step (Δ*G*_298_ +37.9 kcal mol^–1^), again offset by the products being exoenergic by 32.4 kcal mol^–1^. The regeneration step for the second cycle is endoenergic by +24.9 kcal mol^–1^ and the overall barrier originating from TS1 is 25.4 kcal mol^–1^. This makes this model cycle less favourable.

Finally, a variation of the one amine cycle involving utilization of a second amine to form a B–N–B catalytic intermediate was examined (Fig. 13), for which the catalyst assembly step is similarly endoenergic (Δ*G*_298_ +26.0 kcal mol^–1^) to the B–O–B bridged two amine route above. The highest free energy point in the catalytic cycle (TS2, Δ*G*_298_ +23.2 kcal mol^–1^) is now relative to the initial tetrahedral intermediate (TI1), which forms exoenergically from the bicyclic reactant related to the previous cycles, but with a transition state of presumed low barrier corresponding to reactant reorganization which we were unable to locate. This suggests that the barriers resulting from species with B–N–B bridges are similar to those with B–O–B bridges.

These five pathfinding explorations have thus identified a model corresponding to a thermal reaction at 298 K, with the caveat that further optimization of these very complex catalytic combinations may always reveal even lower energy routes. In the future, we plan to exploit these core models to study substituent effects on the energies.

All five of the catalytic cycles investigated have reasonable thermal barriers for the catalytic mechanism (Fig. 14). It is difficult, however, at least purely on the basis of the computed energetics of such catalytic processes, to confidently discriminate between these. Importantly, all these systems have lower overall energetics than the monomeric acyloxyboron pathway previously proposed11*a* and computationally examined.^17^ Finally, on the basis of these calculations, the resting state of the catalyst would be expected to be a bridged (B–O–B or B–N–B) system containing tetrahedral boron atoms (*e.g.* TI2 in Fig. 13), consistent with the signals observed from NMR analysis of catalytic reaction mixtures (*δ*_B_ 3 ppm).

### Kinetic analysis of a catalytic amidation reaction

**Scheme 12:**
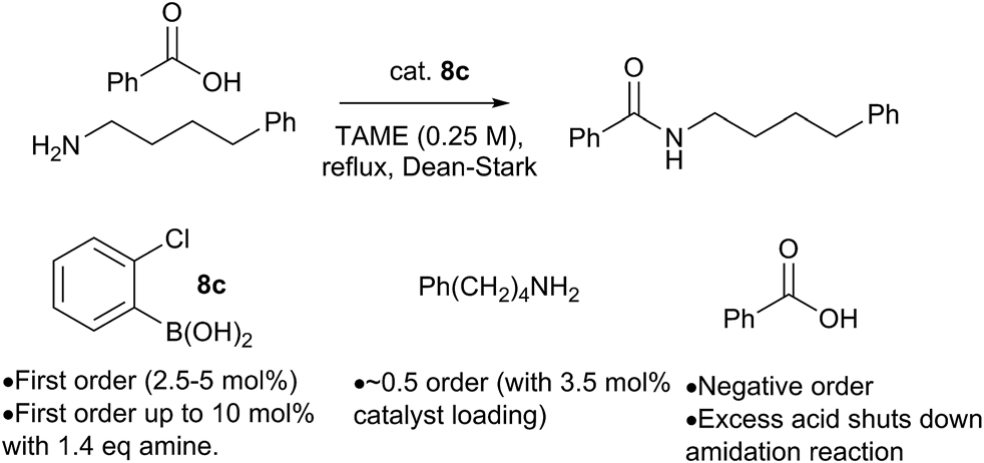
Preliminary kinetics studies on a boronic acid catalyzed amidation reaction.

Preliminary studies of the reaction kinetics for a boronic acid catalyzed amidation reaction revealed some interesting findings (Scheme 12 and ESI†). The selection of reaction conditions and amine/carboxylic acid reactants was guided by the need for a homogenous reaction mixture in order to obtain useful kinetic data. Reaction of 4-phenylbutylamine with benzoic acid12*b* was carried out in *tert*-amyl methyl ether (TAME) under Dean–Stark conditions10*f* using **8c** as the amidation catalyst. Analysis of reaction profiles was carried out using the Burés method (see ESI†).^23^ The reaction was first order in catalyst up to a loading of 5 mol%, but a catalytic loading of 10 mol% did not provide a significant rate acceleration over 5 mol%. Interesting, the first order dependence on catalyst was observed up to 10 mol% loading, when 1.4 equivalents of amine were present. This may suggest that the amine is important for generating the active catalytic species, perhaps related to dimer **12**. A positive order of reaction with respect to the amine concentration was also observed at 3.5 mol% catalyst loading. Unexpectedly, increasing the concentration of carboxylic acid had a negative effect on the reaction rate, and even a slight excess of 1.2 equivalents of carboxylic acid led to almost complete inhibition of the reaction at 3.5 mol% catalyst loading; the effect was less pronounced at higher catalyst loadings.

At present, this observation is difficult to explain, but it appears that excess carboxylic acid prevents formation of the active catalytic species, *e.g. via* formation of off-cycle species such as the ammonium carboxylate salt or dimer **11**. Similar observations regarding catalyst inhibition by carboxylic acid were noted in Zr-catalysed amidation reactions.^24^

All the mechanisms outlined above, as well as those previously proposed in the literature, involve an essentially unimolecular turnover limiting step, so reaction kinetics do not provide a useful tool for distinguishing between them. The fact that the reaction kinetics show a first order dependence on the catalyst concentration is consistent with either a monomeric or dimeric active catalyst, provided it remains as a monomer/dimer throughout the catalytic cycle.23c,^24^ The positive interaction between the concentration of amine and boronic acid catalyst provides some support for mechanisms involving an active species containing both of these components, especially if its formation is dependent on B–N interactions. Crucially, there appears to be a restriction on the quantity of active catalyst that can be generated from free boronic acid (somewhere between 5–10 mol%), which can be lifted at higher amine concentrations.

### Summary and conclusions

We have investigated carboxylic acid/amine/boron catalyst systems experimentally and computationally and have shown the potential importance of both the formation of B–X–B species and of B–N interactions in these systems, leading to the proposal of new catalytic cycles for boron-catalysed amidation reactions involving boronic acids, though by extrapolation, boric acid is highly likely to behave similarly. We have demonstrated that whereas boronic acids can catalyse amidation reactions, borinic acids are ineffective as catalysts themselves, and in fact, undergo protodeboronation instead to derive boronic acids *in situ*. In turn, the boronic acid analogue is catalytically competent and causes downstream amidation catalysis. This work clearly indicates that at least three free coordination sites are needed on the boron atom to enable effective catalysis to take place, and hence, borinic acids are precluded from amidation reactivity. Indeed, our proposed mechanisms can account for this and other observations regarding amidation catalysis. Firstly, the formation of an active dimeric boron catalyst appears to be impossible at the borinic acid oxidation level, as rapid complexation of an acid and amine molecule leads to the formation of a catalytically inactive complex **5**. The success of *ortho*-substituted boronic acids,^13^ including bifunctional systems,^12^ as catalysts can also potentially be rationalized by their preference for the formation of dimeric species over trimeric boroxines. Furthermore, this supports our previous observation that cooperative catalysis using two different boronic acids can lead to enhanced yields of amides in challenging cases.^15^ Our proposed mechanisms are also consistent with a recent report that a complex B/N/O heterocyclic system displays high activity in catalytic amidation reactions.^16^

The findings in this study indicate the clear importance of bringing together multiple techniques to understand the various entities involved in amidation catalysis, *i.e.* the combined use of NMR (^1^H, ^13^C and especially ^11^B), IR, X-ray crystallography, reactivity studies and the importance of examining multiple potential reaction pathways by theoretical methods, as exemplified by the five related key transition states summarised in Fig. 14. In this case, this comprehensive approach has allowed us to move away from a seemingly ‘accepted’ mechanism^17^ which arguably has little supporting experimental evidence,11*a* and to propose instead closely related low energy alternatives. We consider that the mechanism shown in Fig. 13 may be the most likely to be effective under catalytic conditions using boronic acids, due to our experimental observations of the reactivity of dimer **11** with amine, and the direct ^11^B NMR analysis of catalytic reaction mixtures. The key point though is that this is a guide, and individual systems may change between different modes of action depending upon both catalyst and substrate properties (electronics, substituents, *etc.*). However, we anticipate that these mechanistic insights will provide valuable assistance for the design of more active boron amidation catalysts in the future.

## Experimental

### Computational methods

Pathfinder explorations of the potential energy surfaces for putative catalytic cycles all utilised the Def2-TZVPP basis set^25^ and the B3LYP density functional augmented with the Grimme D3 dispersion energy correction,^26^ a combination selected after earlier evaluation for catalytic cycles^27^ and as implemented in the Gaussian 09, versions D01 and E.01 and Gaussian 16 version A.03 programs. A solvent correction using the CPCM method and dichloromethane parameters was employed throughout and all energies are corrected for thermal contributions and entropy by calculation of normal mode frequencies, with transition states exhibiting the required single negative force constant. All free energies correspond to a standard state at 298 K of 0.0445 M (1 atm). Intrinsic reaction coordinates (IRCs) were used to define the start and end species of all transition states. Research data management (RDM) is *via* deposition into a collection-based data repository^28^ where access is *via* the appropriate FAIR data DOI.^29^

### General experimental methods

General experimental details and data files are available *via* a data repository.^29^ Selected experimental procedures and data are included in the ESI.†

### Synthetic methods

All synthetic methods and compound characterization data is available *via* a data repository or in the ESI.† 26

### X-ray crystallography and NMR spectroscopy

Crystallographic and NMR full data files (in Mpublish format) are available *via* a data repository.^29^

## Conflicts of interest

There are no conflicts to declare.

## Supplementary Material

Supplementary information

Crystal structure data
