# Supplementary material for: Mechanistic insights into boron-catalysed direct amidation reactions
Source: Chem Sci. 2018 Jan 2;9(4):1058–72. doi: 10.1039/c7sc03595k (PMC5890798; doi:10.1039/c7sc03595k)
Supplement: Supplementary file 1 [file SC-009-C7SC03595K-s001.pdf]

Electronic Supplementary Material (ESI) for Chemical Science.
This journal is © The Royal Society of Chemistry 2018

Mechanistic insights into boron-catalysed direct amidation reactions

Sergey Arkhipenko,[a] Marco T. Sabatini,[b] Andrei Batsanov,[a] Valerija Karaluka,[b] Tom D. Sheppard,[b] Henry S. Rzepa[c] and Andrew Whiting[a]*

[a]Centre for Sustainable Chemical Processes, Department of Chemistry, Durham University, Science Site, South Road, Durham, DH1 3LE, UK. Email: andy.whiting@durham.ac.uk
[b]Department of Chemistry, University College London, 20 Gordon Street, London, WC1H 0AJ, UK.
[c]Department of Chemistry, Imperial College London, South Kensington Campus, London, SW7 2AZ, UK.

Contents **Page**

1. General experimental 2

2. Synthetic procedures and compound characterisation§

2.A. Reaction of monomeric acyloxyboron species with amines 3

2.B. Synthesis of borinic acids and studies of their interactions with amines and carboxylic acids 6

2.C. Interactions of boronic acids with amines and carboxylic acids 10

2.D. Interaction of B_2O_3 and $B(OH)_3$ with amines and carboxylic acids 21

2.E. Kinetic studies 22

3. Tabulated summary of spectroscopic data 29

4. X-ray crystallographic information# 30

5. References 36

§Note that all NMR data and .cif crystallographic data is available for download from site: DOI: 10.14469/hpc/1620 and sub-collections therein.

GENERAL EXPERIMENTAL

All starting materials and solvents were obtained commercially from standard chemical suppliers and were used as received unless otherwise stated. Dry solvents were prepared using the Innovative Technology Inc. solvent purification system, or dried by known methods, including standing over 4 Å molecular sieves for 24 h in the case of toluene and $CDCl_3$. Reactions were carried out at r.t. unless otherwise stated. Evaporations were carried out at 10-20 mmHg using a rotary evaporator and water bath followed by evaporation to dryness *in vacuo* (<2 mmHg). A magnetic stirrer bar was used for stirring reactions. Crystals suitable for X-ray analysis were obtained by vapour diffusion with EtOAc/pentane, unless otherwise stated.

NMR spectra were recorded using a Bruker Avance-400 MHz spectrometer at frequencies of 400, 101, 128 and 376 MHz for ^{1}H, ^{13}C, ^{11}B and ^{19}F respectively. NMR experiments were run in $CDCl_3$ unless otherwise stated and the data is reported as follows: chemical shift (δ, ppm), multiplicity, spin-spin coupling constants (J, Hz), integration and assignment, where possible. Aromatic carbons next to boron atom are not reported in ^{13}C NMR. Mass spectra were obtained using ASAP (LCT Premier XE), ESI (TQD mass spectrometer with Acquity UPLC photodiode array detector) or EI (Shimadzu QP-2010-Ultra) techniques. Accurate mass values were measured on QtoF Premier mass spectrometer. IR spectra were obtained using FT1600 series or PerkinElmer UATR Two spectrometers. Elemental analysis was performed using an Exeter Analytical E-440 Elemental Analyser. Melting points were determined using an Electrothermal apparatus and were uncorrected. The *in situ* IR spectroscopy monitoring was carried out with ReactIR 4000 instrument equipped with MCT detector; ConcIRT window = 1900 – 900 cm^{-1}. Advance setting: Laser WN = 7901 – 415 cm^{-1}; Apodization = Happ General. Probe: Prob A DiComp (Diamond) connected *via* K6 Conduit (16mm prob); Sampling 4000-6500 at 8 cm^{-1} resolution; Scan option: auto select, gain 2×. 5 Å molecular sieves were dried at 250 °C (<1 mbar) using a Kugelrohr instrument, as detailed by Blanchet *et al.* [3]

2. SYNTHETIC PROCEDURES AND COMPOUND CHARACTERISATION

2.A. Studies of monomeric acyloxyboron species with amines

Reactivity of MIDA-boronate 1c towards amines

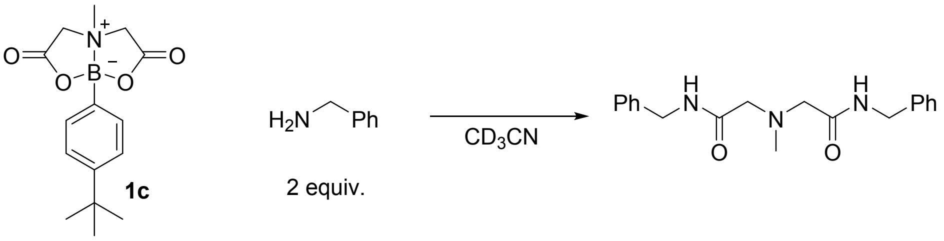

1M Stock solutions of benzylamine (0.2 mL, 0.20 mmol, 2 equiv.) and internal standard (1,4-dimethoxybenzene, 0.1 mL, 0.10 mmol, 1 equiv.) were added to 4-tert-butylphenylboronic acid MIDA ester **2** (29 mg, 0.10 mmol, 1 equiv.), and topped up with CD_3CN (0.3 mL). ^{1}H, ^{11}B and ^{13}C NMR were collected at regular intervals over 7 days at room temperature. The yield of the amide product was determined by integration against the internal standard peaks to be <5% after 2 days.

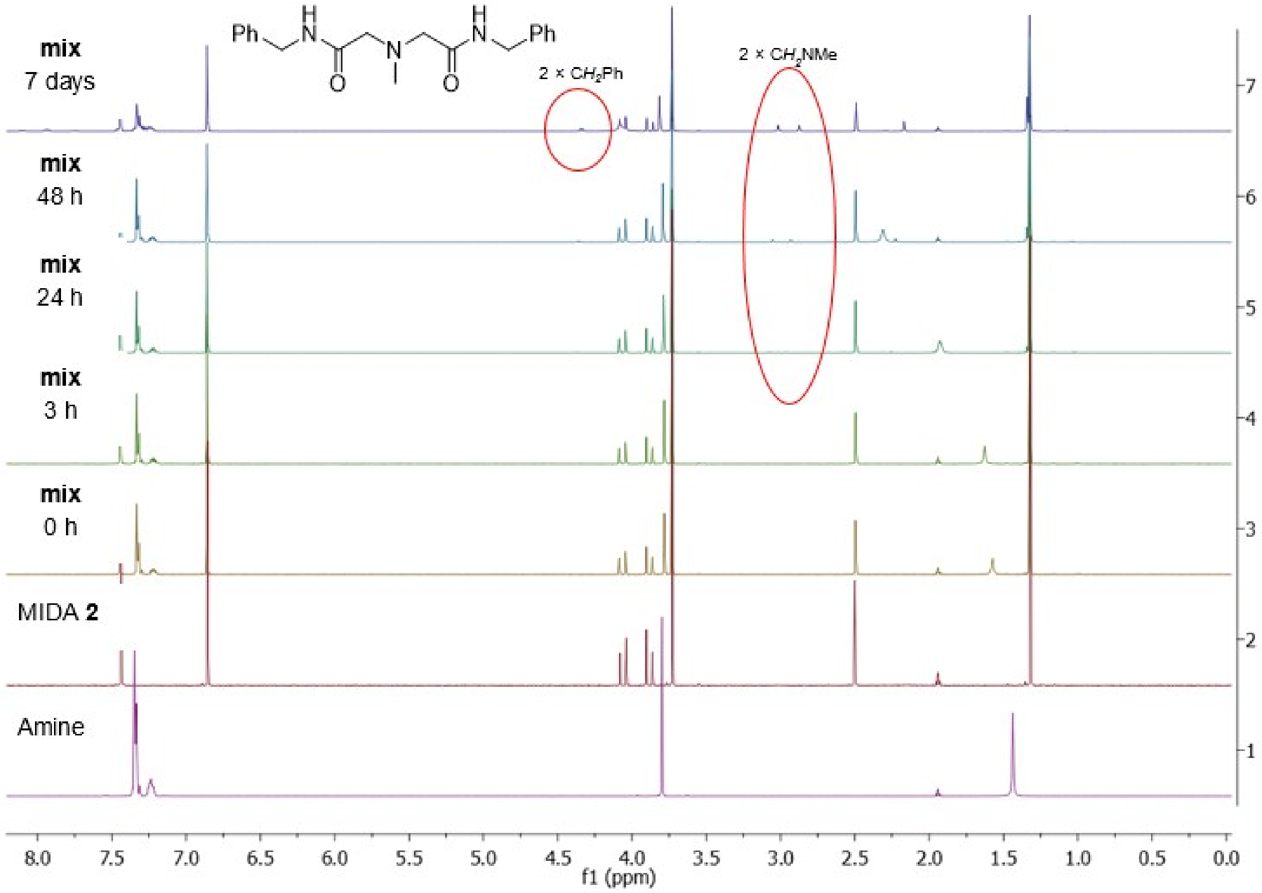

Reactivity of trigonal acyloxyboron species 1a towards amines

Procedure: attempts from dichloro(phenyl)borane

An oven dried NMR tube was filled with mandelic acid (31 mg, 0.2 mmol), 1,4 dimethoxybenzene (0.2 mmol) and 0.3 mL $CDCl_3$. The system was flushed with argon (through the Precision Seal® rubber septa cap). The solution was cooled to 0 °C, and a solution of dichloro(phenyl)borane (30 mg, 0.2 mmol) in 0.3 mL $CDCl_3$ was added. Reaction mixture was brought to RT, and subjected to NMR analysis after 1 h. Caution: reaction is highly exothermic and releases HCl gas: keep argon balloon over the course of the reaction for pressure release.

Once complete conversion to the acyloxyboron species **1a** was observed by ^{11}B NMR (signal 32 ppm), benzylamine (0.2 or 0.4 mmol) was added to the reaction mixture. Reaction mixture was analysed at regular time intervals (0, 2, 4, 24h), but no amide bond formation was observed.

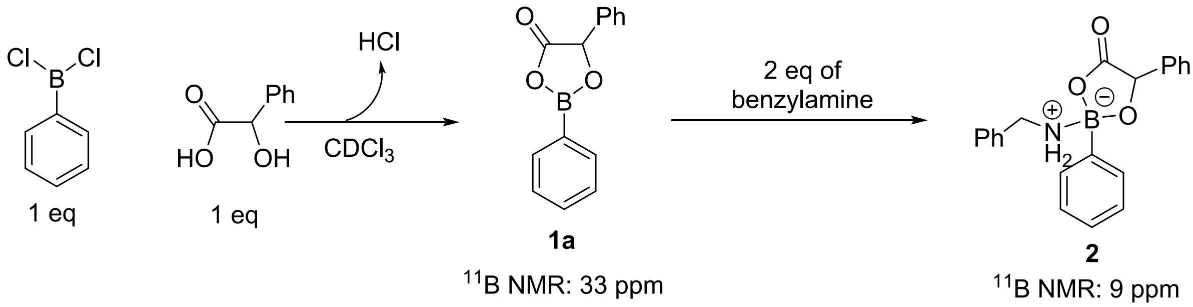

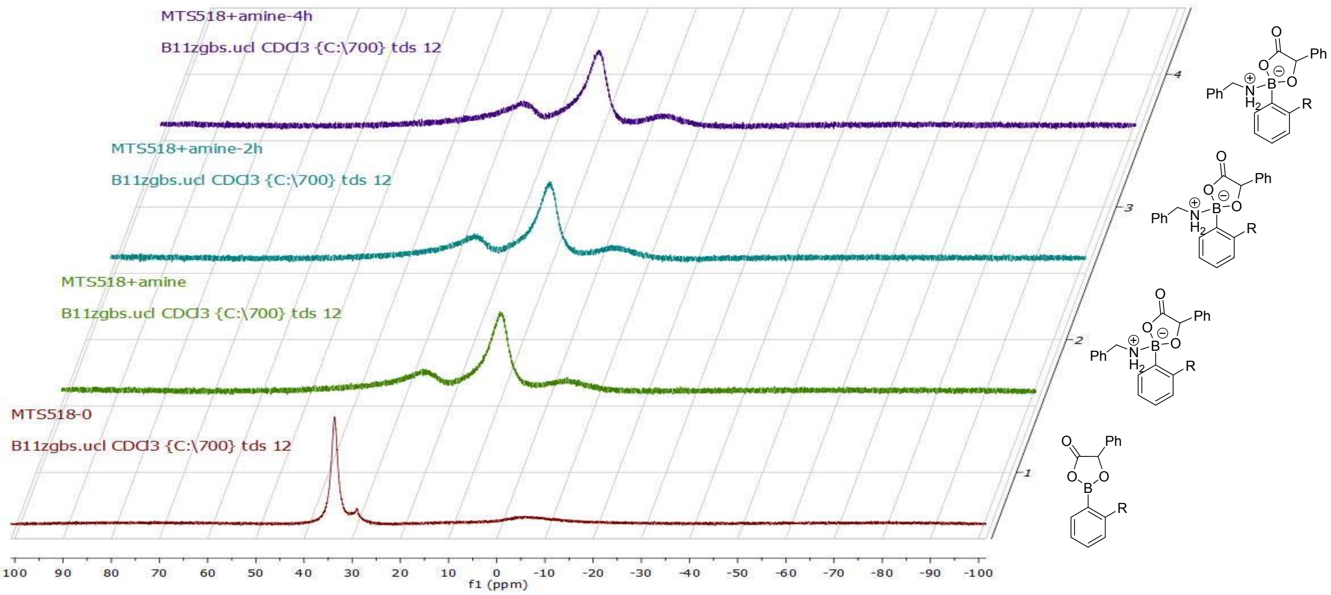

Procedure: attempts from 2-chlorophenylboronic acid

A 6 mL vial equipped with a stirrer was filled with mandelic acid (76 mg, 0.5 mmol), 2-chloro phenylboronic acid (0.1 mmol) and a stock solution of 1,4 dimethoxybenzene (0.125 mmol) in 1 mL $CDCl_3$. The system was flushed with Argon, and further 2 mL of $CDCl_3$ were added. Freshly prepared 5 Å mol. sieves (~1 g) were quickly added.

Once complete conversion to the acyloxyboron species **1a** was observed by ^{11}B NMR (typically 15 min), benzylamine (0.2 or 0.4 mmol) was added to the reaction mixture. Reaction mixture was analysed at regular time intervals (0, 4, 24h), but no amide bond formation was observed.

Note: Heterogeneous amide bond forming reactions of this sort require stirring. Reactions conducted in an NMR tube only have very slow conversion to amide in comparison.

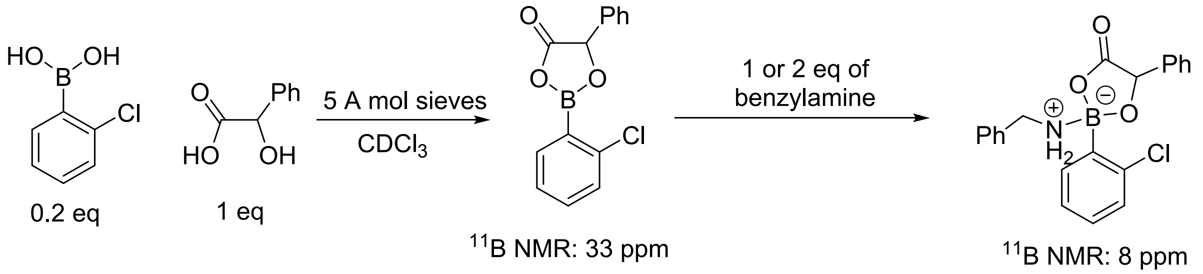

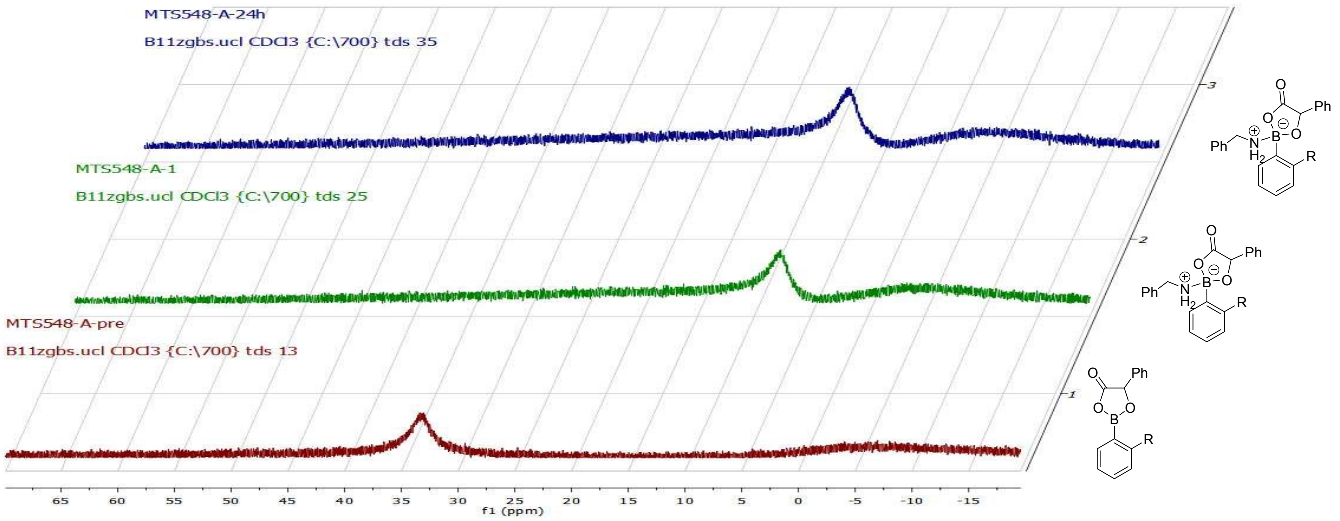

2.B. Synthesis of borinic acids and studies of their interactions with amines and carboxylic acids

Bis-phenylborinic acid – ethanolamine complex ESI1[1]

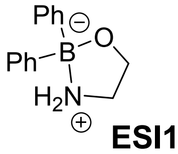

ESI1

To dry THF (20 mL), triisopropylborate (0.5 g, 2.66 mmol) was added at 0 °C, followed by PhMgBr (3 M solution in Et_2O, 5.32 mmol). The reaction mixture was warmed to r.t., after 16 h 5% aq HCl (8 mL) was added, the mixture was stirred for 10 min and extracted with ether. After reducing the volume to ca. 5 mL *in vacuo*, diethanolamine (1 M solution in ^{i}PrOH, 2.66 mmol) was added. After 30 min the mixture was evaporated and recrystallization from EtOH yielded compound **ESI1** (0.363 g, 56%). ^{1}H NMR δ 7.50 – 7.46 (m, 4H, C_{Ar}-H), 7.34 – 7.29 (m, 4H, C_{Ar}-H), 7.27 – 7.21 (m, 2H, C_{Ar}-H), 4.22 – 4.03 (br s, 2H, NH_2), 4.03 (t, J = 6.3 Hz, 2H, CH_2), 3.07 – 3.00 (m, 2H, CH_2). ^{11}B NMR δ 6.
Data in accordance with the literature[1]

Synthesis of other borinic acid – ethanolamine complexes

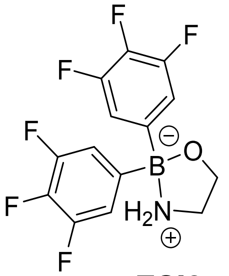

ESI2

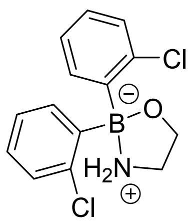

ESI3

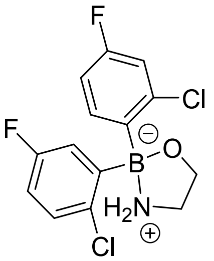

ESI4

Mg turnings (2.1 equiv.) were stirred under Ar for 30 mins, followed by the addition of anhydrous THF (1 mL per 1 mmol of halide) and a crystal of I_2. Part of aryl halide (0.1 equiv.) was added and the mixture was heated to ca. 40 °C until the start of reaction, indicated by disappearance of the iodine colour. Then, a mixture of remaining aryl halide (1.9 equiv.) and trimethylborate (1 equiv.) was added dropwise, and the reaction mixture was left to stir at r.t. overnight. Then 5% aq HCl was added, mixture was washed with Et_2O twice, organic fractions combined, dried and evaporated. The residue was then redissolved in IPA and ethanolamine (1 equiv.) was added. Subsequent addition of Et_2O or hexane led to crystallisation of product as white solid.

Bis-(3,4,5-trifluorophenyl)borinic acid ethanolamine complex **ESI2**: 59%. Mp 210 – 212 °C. ^{1}H NMR δ 7.09 – 6.97 (m, 4H, C_{Ar}-H), 4.15 – 4.04 (m, 2H, CH_2), 3.31 – 3.16 (br s, 2H, CH_2). ^{11}B NMR δ 5. ^{13}C NMR (DMSO-d_6) δ 155.2 (ddd, J = 248.9, 9.3, 2.3 Hz, CH-$\underline{C}$F-CF), 142.4 (dt, J = 246.4, 15.5 Hz, CF-$\underline{C}$F-CF), 119.6 – 119.2 (m, $\underline{C}$H-CF), 67.9, 46.7. ^{19}F NMR δ -135.51 – -135.66 (m, 4F, *m*-C_{Ar}-F), -163.26 – -163.45 (m, 2F, *p*-C_{Ar}-F). IR ν_{max} (neat)/cm^{-1} 3347, 3086, 2872, 1607, 1520, 1405, 1318, 1206, 1131, 1071, 1031, 992, 862, 783, 726, 670. Calc. for $C_{14}H_{10}BF_6NO$: C, 50.5; H, 3.0; N, 4.2. Found: C, 49.9; H, 3.1; N, 4.3.

Bis-(2-chlorophenyl)borinic acid ethanolamine complex **ESI3**:41%. Mp 213 – 215 °C. ^{1}H NMR δ 7.38 – 7.33 (m, 2H, C_{Ar}-H), 7.32 – 7.29 (m, 2H, C_{Ar}-H), 7.23 – 7.15 (m, 4H, C_{Ar}-H), 5.40 – 5.05 (br s, 2H, NH_2), 4.09 (t, J = 6.5 Hz, 2H O-CH_2), 3.24 (p, J = 6.5 Hz, 2H, N-CH_2). ^{11}B NMR δ 6. ^{13}C δ NMR 137.9, 136.0, 129.2, 128.6, 126.3, 63.3, 41.6. IR ν_{max} (neat)/cm^{-1} 3305, 2862, 1580, 1418, 1279, 1250, 1198, 1148, 1076, 1024, 919, 767, 745, 724. Calc. for $C_{14}H_{14}BCl_2NO$: C, 57.1; H, 5.0; N, 4.6. Found: C, 57.2; H, 4.8; N, 4.8.

Bis-(2-chloro-4-fluorophenyl)borinic acid ethanolamine complex **ESI4**: 11%. Mp 188 – 190 °C. ^{1}H NMR δ 7.32 – 7.25 (m, 2H, C_{Ar}-H), 7.10 – 7.05 (m, 2H, C_{Ar}-H), 6.95 – 6.88 (m, 2H, C_{Ar}-H), 5.38 – 4.90 (br s, 2H, NH_2), 4.09 (t, J = 6.5 Hz, 2H O-CH_2), 3.27 (p, J = 6.5 Hz, 2H, N-CH_2). ^{11}B NMR δ 6. ^{13}C δ NMR 162.2 (d, J = 248.4 Hz, $\underline{C}$F), 137.8 (d, J = 9.6 Hz), 136.9 (d, J = 7.8 Hz), 116.5 (d, J = 23.5 Hz), 113.6 (d, J = 18.9 Hz),

63.2, 41.7. ^{19}F NMR δ -113.97 – -114.08 (m). ^{11}B NMR δ 6. IR ν_{max} (neat)/cm^{-1} 3065, 1678, 1588, 1473, 1373, 1251, 1197, 1069, 1033, 932, 888, 849, 823, 798, 746, 701.

Synthesis of borinic acids 3

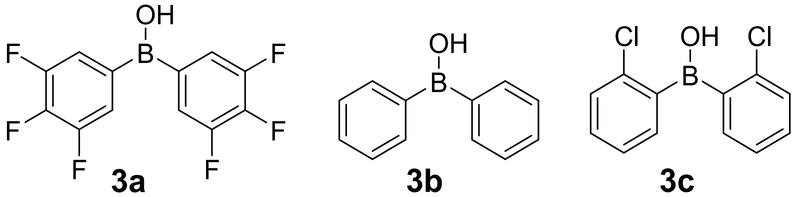

3a **3b** **3c**

Ethanolamine complex **ESI1-3** (0.3 mmol) was dissolved in a 1:1 mixture of acetone and MeOH (2 ml), followed by 5% aq HCl (3 mL). The mixture was stirred for 5 min, extracted with Et_2O (10 mL, x2), the organic layers were combined, dried and evaporated to yield borinic acids **3** as colourless oils.

Bis-(3,4,5-trifluorophenyl)borinic acid **3a**: 82%. Mp 68.5 – 69 °C. ^{1}H NMR δ 7.40 – 7.32 (m, 4H, C_{Ar}-H), 6.11 (br s, 1H, OH). ^{13}C NMR δ 151.4 (ddd, J = 253.4, 9.6, 2.9 Hz, CH-CF), 142.2 (dt, J = 257.4, 15.3 Hz, CH-CF-CF), 118.4 – 118.1 (m, CH-CF). ^{11}B NMR δ 44. ^{19}F NMR δ -133.58 – -133.71 (m, 4F, CH-CF), -155.00 – -155.17 (m, 2F, CH-CF-CF). IR ν_{max} (neat)/cm^{-1} 3611, 1610, 1528, 1419, 1349, 1191, 1036, 972, 864, 713, 622. Calc. for $C_{12}H_5BOF_6$: C, 49.7; H, 1.7. Found: C, 49.9; H, 1.75.

Bis-(phenyl)borinic acid **3b**: 89%. ^{1}H NMR δ 7.90 – 7.82 (m, 4H, C_{Ar}-H), 7.54 – 7.43 (m, 6H, C_{Ar}-H), 6.03 – 5.80 (br s, 1H, OH). ^{11}B NMR δ 46.

Data in accordance with the literature[2]

Bis-(2-chlorophenyl)borinic acid **3c**: 83%. ^{1}H NMR δ 7.50 – 7.45 (m, 2H, C_{Ar}-H), 7.45 – 7.40 (m, 4H, C_{Ar}-H), 7.34 – 7.29 (m, 2H, C_{Ar}-H). ^{11}B NMR δ 45.

Data in accordance with the literature[2]

Synthesis of bis-(3,4,5-trifluorophenyl)borinic acid – amine complexes 4

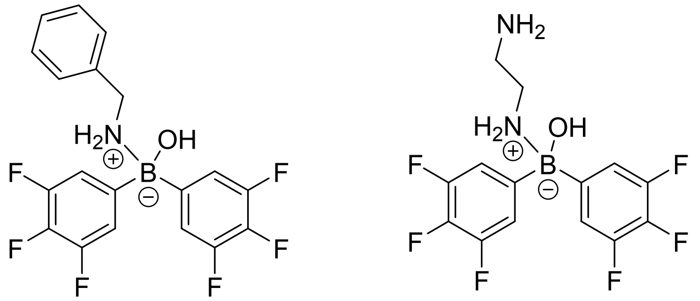

4a **4b**

To DCM (10 mL), bis-(3,4,5-trifluorophenyl)borinic acid **3a** (0.1 g, 0.34 mmol) was added, followed by amine (0.34 mmol). The reaction mixture was stirred at r.t. for 5 min and evaporated to dryness. The crude product was used without further purification. Crystals suitable for X-Ray analysis were obtained by slow recrystallization from DCM.

Bis-3,4,5-trifluorophenylborinic acid benzylamine complex **4a**: Mp 118 – 122 °C. ^{1}H NMR 7.38 – 7.43 (m, C_{Ar}-H, 3H), 7.16 – 7.21 (m, C_{Ar}-H, 2H), 6.93-7.00 (m, 4H, $(C_6F_3H_2)_2$), 3.77 (s, 2H, CH_2), 2.63 – 3.89 (br s, 2H, NH_2). ^{13}C NMR δ 151.2 (dm, J=251.8 Hz, CH-CF-CF), 137.5 – 140.0 (m, CF-CF-CF), 135.6, 129.6, 129.2, 128.1, 114.5 – 114.9 (m, CH-CF), 45.9. ^{11}B NMR δ 3. ^{19}F NMR δ -135.11 – -135.43 (br s, 4F, CH-CF-CF), -162.82 – -163.44 (br s, 2F, CF-CF-CF). IR ν_{max} (neat)/cm^{-1} 3679, 2957, 1610, 1522, 1403, 1319, 1024, 734, 700. Calc. for $C_{19}H_{14}BNOF_6$: C, 57.5; H, 3.55; N, 3.5. Found: C, 57.5; H, 3.5; N, 3.5.

Bis-3,4,5-trifluorophenylborinic acid ethylenediamine complex **4b**: Mp 88 – 93 °C. ^{1}H NMR 7.04 – 6.95 (m, 4H, C_{Ar}-H), 2.80 (s, 4H, 2×CH_2), 2.23 – 1.59 (br s, 4H, NH_2). ^{13}C NMR 150.0 (dd, J = 247.4, 8.8 Hz, CH-CF-CF), 136.9 (dm, J = 244.3 Hz, CH-CF-CF), 115.1 – 114.7 (m, CH-CF), 44.1. ^{11}B NMR δ 1. ^{19}F NMR δ -135.83 – -136.05 (m, 4F, CF-CH), -164.01 – -164.27 (m, 2F, CF-CF-CF). IR ν_{max} (neat)/cm^{-1} 2962, 1607, 1520, 1405, 1316, 1104, 1023, 983, 909, 852, 759, 736, 688.

Synthesis of borinic acid aminocarboxylate complexes 5:

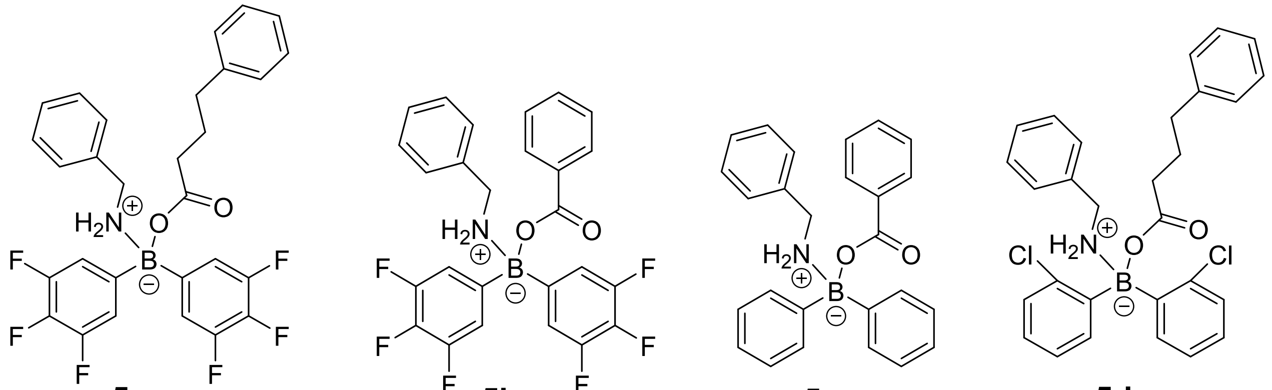

5a **5b** **5c** **5d**

Borinic acid (1 equiv.) was dissolved in DCM at r.t. (10 mL). Amine was added (1 equiv.), followed by carboxylic acid (1 equiv), and after 5 min stirring the mixture was evaporated to dryness. The crude products were used without further purification.

Bis(3,4,5-trifluorophenyl)-borinic acid benzylamine-4'-phenylbutanoate **5a**: Mp 162–163 °C. ^{1}H NMR δ 7.37 – 7.42 (m, 3H, C_{Ar}-H), 7.29 – 7.35 (m, 2H, C_{Ar}-H), 7.19 – 7.25 (m, 3H, C_{Ar}-H), 7.13 – 7.18 (m, 2H, C_{Ar}-H), 6.91-6.98 (m, 4H, CH-CF), 5.65 – 5.75 (br s, NH_2), 3.60-3.64 (m, 2H, NH_2-CH_2), 2.69 (t, J=7.5 Hz, 2H, CO-CH_2), 2.48 (t, J = 7.5 Hz, 2H, CO-CH_2-CH_2-CH_2), 2.03 (p, J = 7.5 Hz, 2H, CO-CH_2-CH_2). ^{13}C NMR δ 178.8, 151.2 (ddd, J = 250.9, 12.5, 2.5 Hz, CH-CF-CF), 141.2, 138.8 (dt, J = 250.9, 12.5 Hz, CF-CF-CF), 134.1, 129.6, 129.5, 128.5, 128.4, 128.4, 126.1, 114.4 – 114.6 (m, CH-CF), 45.8, 36.0, 35.3, 26.7. ^{11}B NMR δ 1. ^{19}F NMR δ -135.41 – -135.53 (m, 4F, CH-CF-CF), -162.99 – -163.13 (m, 2F, CF-CF-CF). IR ν_{max} (neat)/cm^{-1} 3262, 1677, 1524, 1409, 1322, 1273, 1030, 754, 700, 681. ESI-LRMS (negative ion) *m/z* 542.16 [M-H], 453.12 [M-$PhCH_2$], 289.12 [M-$PhCH_2NH_2$-$Ph(CH_2)_3CO$], 163.09 [$Ph(CH_2)_3COO$]. HRMS: Calcd for $C_{29}H_{23}{}^{10}BNO_2F_6$ 541.1762, found 541.1757. Calc. for $C_{29}H_{24}BNO_2F_6$: C, 64.1; H, 4.45; N, 2.6. Found: C, 63.8; H, 4.5; N, 2.5.

Bis(3,4,5-trifluorophenyl)-borinic acid benzylaminobenzoat **5b**: Mp 197 – 200 °C. ^{1}H NMR 8.21 – 8.24 (m, 2H, C_{Ar}-H), 7.63 – 7.69 (m, 1H, C_{Ar}-H), 7.53 – 7.59 (m, 3H, C_{Ar}-H), 7.40 – 7.43 (m, 2H, C_{Ar}-H), 7.22 – 7.26 (m, 2H, C_{Ar}-H), 7.01 – 7.08 (m, 4H, CF-CH), 5.70 (br s, 2H, NH_2), 3.72 – 3.77 (m, 2H, CH_2). ^{13}C NMR δ 170.9, 160.0, 151.0, 134.2, 133.3, 131.8, 129.9, 129.7, 129.6, 128.6, 128.5, 122.9, 114.4 – 114.6 (m, CH-CF), 45.9. ^{11}B NMR δ 2. ^{19}F NMR δ -135.09 – -135.20 (m, 4F, CH-CF-CF), -162.71 – -162.86 (m, 2F, CF-CF-CF). IR ν_{max} (neat)/cm^{-1} 3291, 3201, 1665, 1575, 1521, 1410, 1318, 1266, 1135, 1011, 758, 700, 647. ESI-LRMS (negative ion) *m/z* 500.10 [M-H], 424.09 [M-Ph], 380.11 [M-$PhCO_2$]. HRMS: Calcd for $C_{26}H_{17}{}^{10}BNO_2F_6$ 499.1293, found 499.1296, calcd for $C_{20}H_{13}{}^{10}BNO_2F_6$ 423.0998, found 423.0980, calcd for $C_{19}H_{13}{}^{10}BNF_6$ 379.1100, found 379.1082. Calc. for $C_{26}H_{18}BNO_2F_6$: C, 62.3; H, 3.6; N, 2.8. Found: C, 61.6; H, 3.6; N, 2.8.

Bis-(phenyl)borinic acid benzylaminobenzoate **5c**: Mp 107 – 108 °C. ^{1}H NMR (50% purity; assignment on the basis of comparison with benzylamine and benzoic acid ^{1}H NMR data) δ 8.34 – 8.27 (m, 2H, C_{Ar}-H), 8.11 – 8.00 (m, 8H, C_{Ar}-H), 7.48 – 7.40 (m, 10H, C_{Ar}-H), 5.92 – 5.68 (br m, 2H, NH_2), 3.86 – 3.76 (m, 2H, CH_2). ^{11}B NMR δ 4. IR ν_{max} (neat)/cm^{-1} 3239, 3033, 1693, 1638, 1574, 1514, 1387, 1349, 1312, 1260, 1154, 963, 891, 844, 756, 697, 656.

Bis-(2-chlorophenyl)borinic acid benzylamine-4-phenylbutanoate **5d:** Mp 136 – 137 °C. ^{1}H NMR δ 7.64 – 7.58 (m, 2H, C_{Ar}-H), 7.45 – 7.38 (m, 4H, C_{Ar}-H), 7.34 – 7.28 (m, 4H, C_{Ar}-H), 7.25 – 7.13 (m, 10H, C_{Ar}-H),

6.68 – 6.47 (br m, 2H, NH_2), 3.66 – 3.59 (m, 2H, N-CH_2), 2.65 (t, J = 7.4 Hz, 2H, OC-CH_2), 2.47 (t, J = 7.4 Hz, 2H, -CH_2), 2.04 (p, J = 7.4 Hz, 2H, OC-CH_2-CH_2-C$\underline{H_2}$), ^{13}C NMR δ 178.0, 141.7, 137.1, 135.4, 135.0, 129.4, 129.3, 128.9, 128.5, 128.4, 128.3, 128.2, 125.9, 125.9, 45.8, 36.1, 35.3, 26.8. ^{11}B NMR δ 2. IR ν_{max} (neat)/cm^{-1} 3281, 3198, 1678, 1591, 1388, 1370, 1338, 1279, 1244, 1188, 1006, 922, 851, 745, 700, 629.

Borinic 3c and boronic 8c acids in direct amide formation

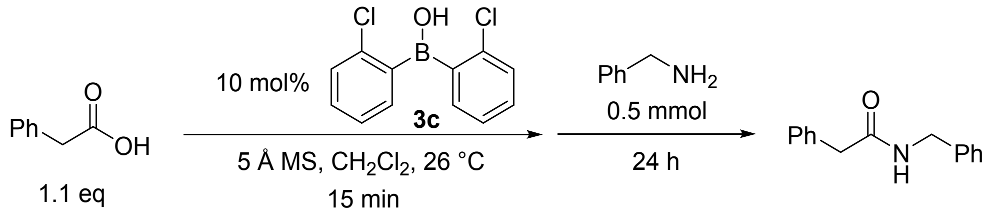

Phenylacetic acid (75 mg, 0.55 mmol), borinic acid **3c** (13 mg, 0.05 mmol) and 1.0 g of powdered 5Å molecular sieves (activated at 250 °C at 4.8^{-1} mbar for 3 h in a Kugelrohr apparatus) were vigourously stirred in CH_2Cl_2 (7 mL) for 15 min at 26 °C under inert atmosphere. Benzylamine (55 µL, 0.50 mmol) was added and the resulting mixture stirred for 24 h. The suspension was filtered through a pad of celite, washed with CH_2Cl_2 (3 × 5 mL) and concentrated *in vacuo*. Conversion was determined to be 90% by NMR.

The identical experiment without 15 min stirring, i.e. when benzylamine was added immediately, yielded only trace (< 0.5%) of amide product after 48 h of stirring at r.t. – see Scheme **6A and 6B** in the article for comparison of reaction mixture by ^{11}B NMR after 15 min.

Following the procedure reported above, but using (2-chlorophenyl)boronic acid **8c** as a catalyst instead of borinic acid **3c**, conversion was determined to be 91% by NMR.

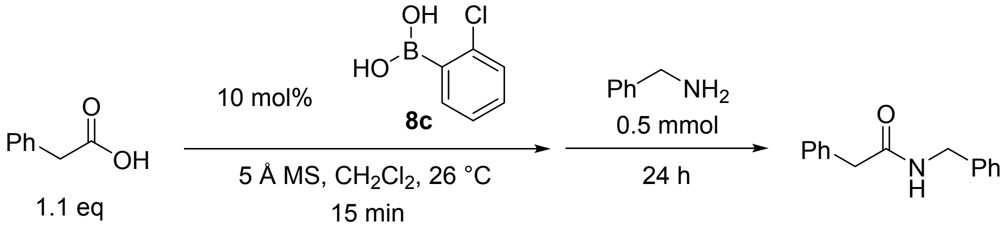

Bis-(2-chlorophenyl)borinic acid protodeboronation
Borinic acid **3c** (0.07 mmol) was added under Ar atmosphere to the NMR tube containing dry $CDCl_3$ (0.7 mL) and powdered activated 5 Å MS (1 g). Phenylacetic acid (0.08 mmol) was added, and the suspension was vigorously stirred for 15 min, followed by filtration through celite and evaporation to yield boronic and borinic products in 6:1 ratio, as seen in Scheme **6C)** in the article.

Bis-(3,4,5-trifluorophenyl)borinic acid protodeboronation
Bis-(3,4,5-trifluorophenyl)borinic acid **3a** (0.07 mmol) was added to the NMR tube, followed by dry $CDCl_3$ (0.7 mL) and carboxylic acid (0.07 mmol; 4-phenylbutyric or benzoic acid). NMR analysis was performed in 5 min and again 6 days upon mixing, see Scheme 5 and Fig. 3 in the article. After 6 days crystals suitable for X-ray analysis were formed in NMR tube, allowing identification of boroxine **7a**.

2.C. Interactions of boronic acids with amines and carboxylic acids

Synthesis of 3,4,5-trifluorophenylboroxine – amine complexes 10a-b

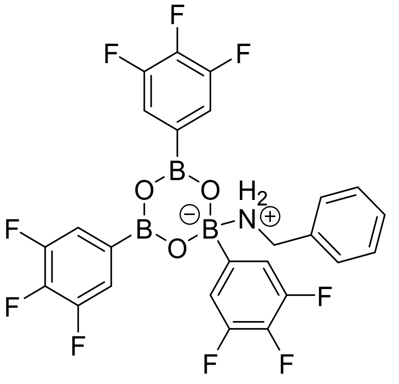

10a

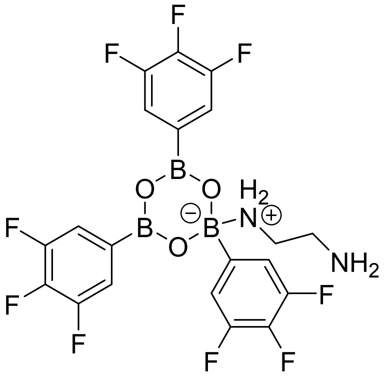

10b

3,4,5-Trifluorophenylboronic acid (0.10 mmol) was added to the NMR tube, followed by dry $CDCl_3$ (0.7 mL) and amine (0.03 mmol benzylamine or ethylenediamine). The tube was reversed 10 times, full dissolution was not achieved. Over time crystals suitable for X-ray analysis were formed in the tube.

3,4,5-Trifluorophenylboroxine – benzylamine complex 10a
Mp 205 – 207 °C. ^{1}H NMR δ 7.58 – 7.48 (m, 6H, CF-CH), 7.43 – 7.32 (m, 3H, NC…C_{Ar}-H), 7.28 – 7.23 (m, 2H, NC…C_{Ar}-H), 3.93 (s, 2H, CH_2), 3.09 – 2.24 (br s, 2H, NH_2). ^{13}C NMR δ 151.2 (dm, J = 251.2 Hz, CH-CF-CF), 141.2 (dm, J = 254.4 Hz, CF-CF-CF), 137.2, 129.3, 128.6, 127.7, 117.0 – 116.7 (m, CH-CF), 45.6. ^{11}B NMR δ 19. ^{19}F NMR δ -135.30 – -135.45 (br m, 4F, CH-CF-CF), -158.11 – -158.46 (br m, 2F, CF-CF-CF). IR ν_{max} (neat)/cm^{-1} 3337, 2981, 1612, 1526, 1430, 1357, 1323, 1268, 1220, 1131, 1030, 1019, 859, 762, 725, 700, 662, 615. Calc. for $C_{25}H_{15}B_3F_9NO_3$: C, 51.7; H, 2.6; N, 2.4. Found: C, 51.6; H, 2.6 N, 2.4.

3,4,5-Trifluorophenylboroxine – ethylenediamine complex 10b
Mp 241 – 242 °C. ^{1}H NMR δ 7.46 – 7.41 (m, 6H, CF-CH), 2.73 (s, 4H, 2×CH_2), 1.78 – 1.15 (br s, 4H, NH_2). ^{13}C NMR (CF-CF-CF carbon not reported) δ 151.1 (dm, J = 250.3 Hz, CH-CF), 116.4 – 116.2 (m, CH-CF), 44.2. ^{11}B NMR δ 19. ^{19}F NMR δ -136.00 – -136.18 (m, 6F, CH-CF), -160.21 – -160.51 (br s, 3F, CH-CF-CF). IR ν_{max} (neat)/cm^{-1} 3349, 2964, 1607, 1583, 1518, 1425, 1408, 1352, 1314, 1273, 1211, 1137, 1018, 901, 874, 782, 720, 659.

Synthesis of phenylboroxine complex with 2 equiv. benzylamine 10c

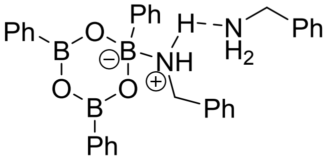

10c

Phenylboronic acid (0.2 g, 1.64 mmol) and benzylamine (0.351 g, 3.28 mmol) were added to toluene (10 mL) and mixture was refluxed for 2 h. Obtained solution was evaporated to yield 0.51 g of pale yellow oil, which crystallised overnight. ^{1}H NMR δ 8.15 – 8.06 (m, 6H, C_{Ar}-H), 7.52 – 7.43 (m, 9H, C_{Ar}-H), 7.40 – 7.30 (m, 6H), 7.25 – 7.17 (m, 4H), 3,90 (s, 4H) ^{13}C NMR δ 138.9, 133.8, 129.8, 129.02, 127.9, 127.7, 127.7, 45.4. ^{11}B NMR δ 20. Calc. for $C_{25}H_{24}B_3NO_3$: C, 73.1; H, 6.3; N, 5.3. Found: C, 72.3; H, 6.3; N, 5.2.

Synthesis of phenylboroxine complex with 4 equiv. benzylamine 10d

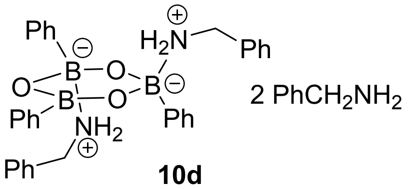

10d

Phenylboronic acid (2.0 g, 16.4 mmol) and benzylamine (3.5 g, 32.8 mmol) were added to toluene (60 mL) and mixture was refluxed for 2 h with Dean-Stark apparatus. Obtained solution was evaporated to yield 5.2 g of colourless oil, which crystallised overnight. Mp 56 – 56.5 °C. ^{1}H NMR δ 8.12 – 8.05 (m, 6H, B-C_{Ar}…$C_{Ar}\underline{H}$) 7.48 – 7.42 (m, 9H, B-C_{Ar}…$C_{Ar}\underline{H}$) 7.39 – 7.32 (m, 8H, N-C_{Ar}…$C_{Ar}\underline{H}$), 7.32 – 7.25 (m, 12H, N-C_{Ar}…$C_{Ar}\underline{H}$), 3.89 (s, 8H, CH_2), 2.27 – 2.01 (br s, 8H, NH_2). ^{13}C NMR δ 141.6, 133.7, 129.7, 128.7, 127.6, 127.3, 127.3, 46.0. ^{11}B NMR δ 20. IR ν_{max} (neat)/cm^{-1} 3368, 3310, 3267, 3025, 1607, 1495, 1440, 1359, 1304, 1257, 1210, 1192, 1125, 1016, 911, 843, 729, 701, 672, 614.

Physical interactions of amines with boronic acids:

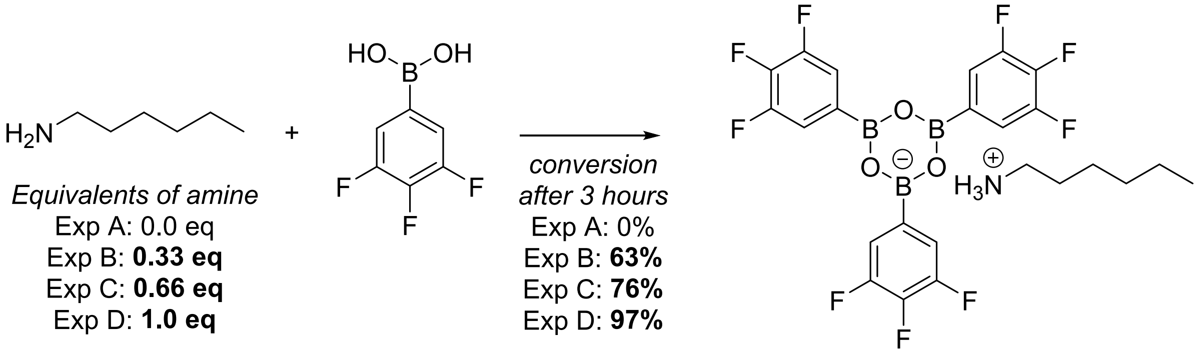

Note: If tubes are shaken, conversion into boroxine is complete after a few minutes only.

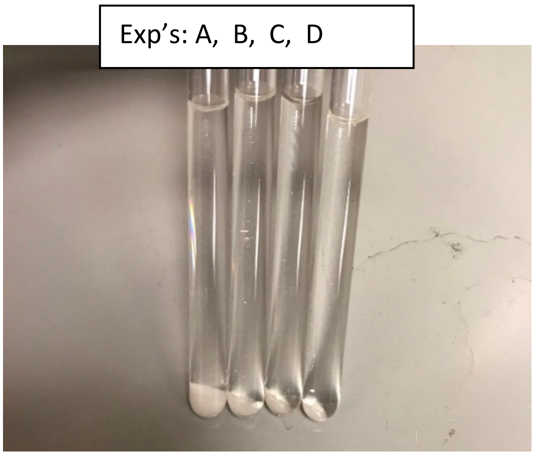

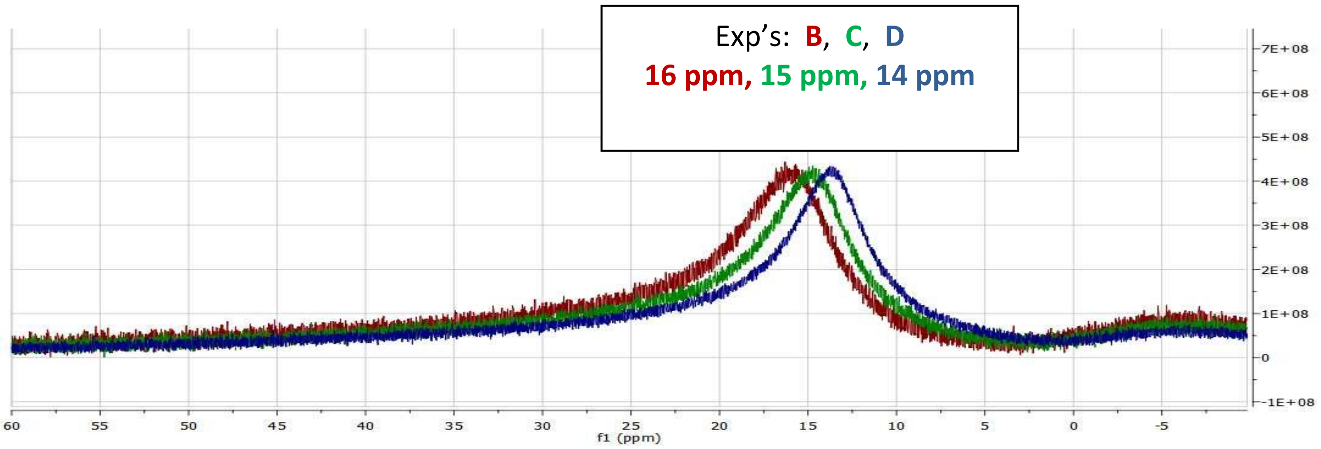

Monitoring of B-O-B species formation by NMR

2-Chlorophenylboronic acid **8c** (0.12 mmol) was added under Ar atmosphere to the NMR tube containing dry $CDCl_3$ (7 mL), followed by phenylacetic acid (0.12 mmol, 16.3 mg). A very small amount of powdered activated 5 Å MS (< 0.5 mm from the bottom of the NMR tube after settling down) was added to the mixture. After 15 min a further amount of powdered activated 5 Å MS (to reach 2 mm from the bottom of the NMR tube) was added. Finally, more 5 Å MS were added to reach 6 mm from the bottom of the NMR tube.

Analysis of crude reaction mixtures

^{11}B NMR spectra showing interactions between 2-chlorophenylboronic acid **8c**, phenylacetic acid and 5 Å MS. **A**: 2-chlorophenylboronic acid; **B**: addition of 5 Å MS to (A), leading to boroxine formation; **C**: addition of a tiny amount of 5 Å MS to a mixture of boronic and carboxylic acid; **D**: addition of more 5 Å MS; **E**: addition of even more 5 Å MS.

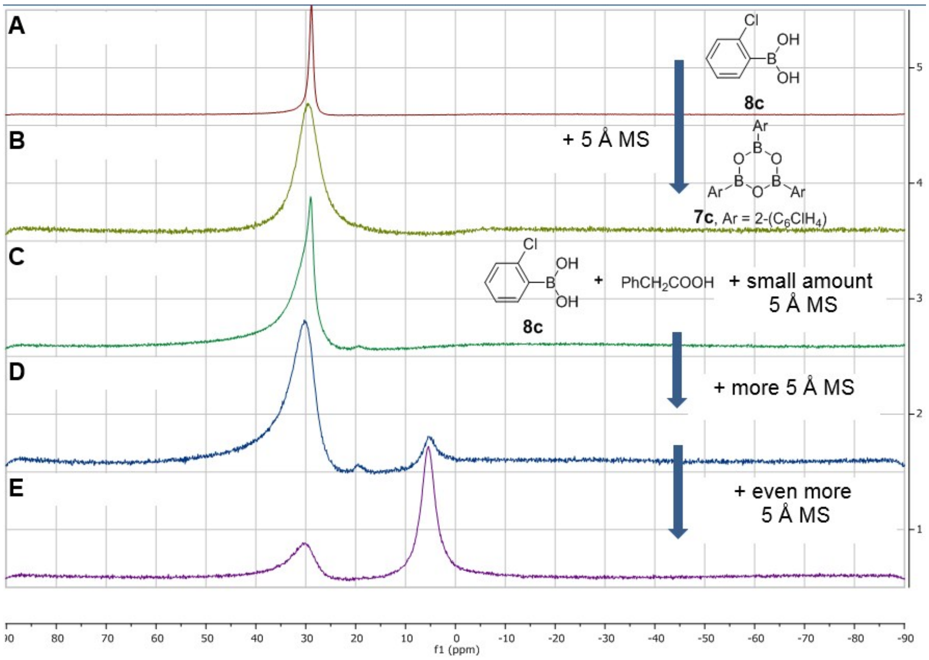

^{1}H NMR spectra showing interactions between 2-chlorophenylboronic acid **8c**, phenylacetic acid and 5 Å MS. **A**: 2-chlorophenylboronic acid; **B**: addition of 5 Å MS to (A), leading to boroxine formation; **C**: addition of a tiny amount of 5 Å MS to a mixture of boronic and carboxylic acid; **D**: addition of more 5 Å MS; **E**: addition of even more 5 Å MS.

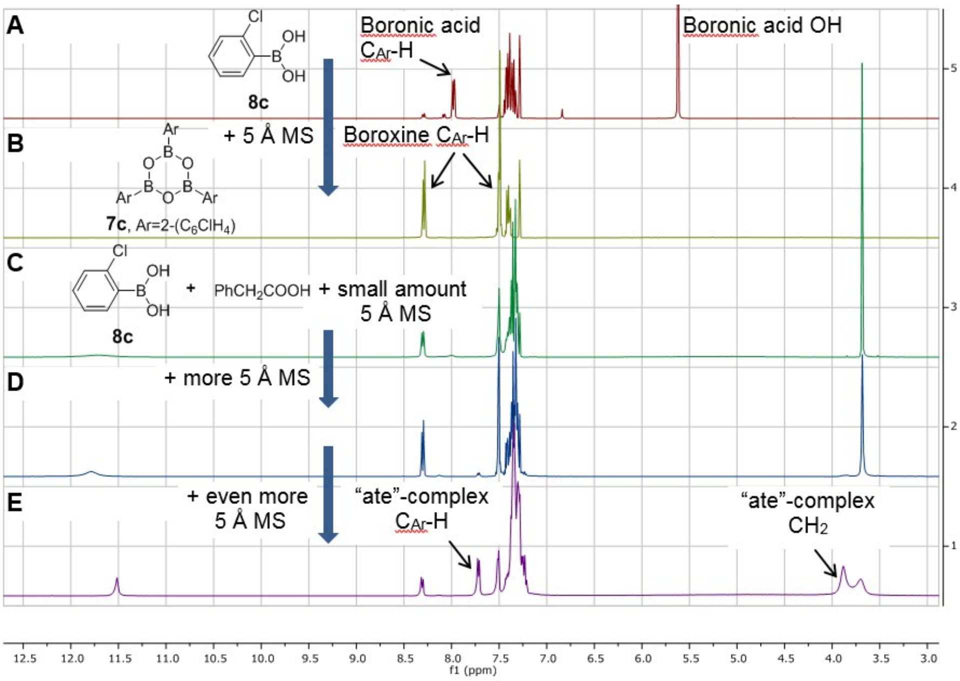

Interaction of 3,4,5-trifluorophenylboronic acid 8a with phenylacetic acid

3,4,5-trifluorophenylboronic acid 8a (0.12 mmol) was added under Ar atmosphere to the NMR tube containing dry $CDCl_3$ (0.7 mL), followed by phenylacetic acid (0.12 mmol, 16.3 mg). Full dissolution was not achieved. Powdered activated 5 Å MS (4 mm from the bottom of the NMR tube after settling down) were added and the mixture was left to stand for 3 min before running NMR analysis.

^{11}B NMR spectra of A): 3,4,5-trifluorophenylboronic acid **8a;** B): addition of phenylacetic acid to A) in the presence of 5 Å MS promotes transformation to "ate"-complex of type **11** and the solubility of boron species in $CDCl_3$ is increased.

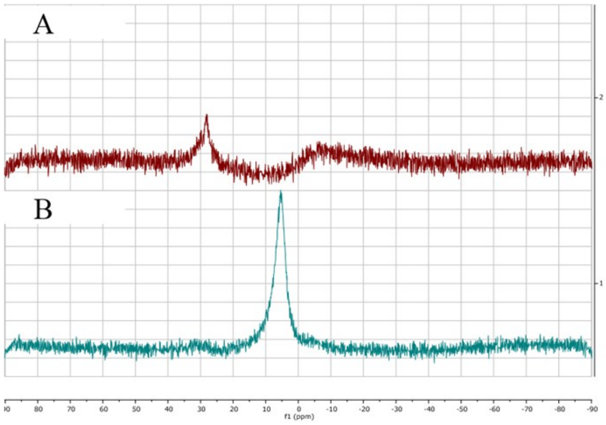

Synthesis of B-O-B dicarboxylate complexes 11 Spectroscopic data of isolated B-O-B complexes

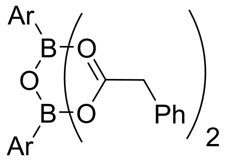

11

c; Ar = 2-(C_6ClH_4)
d; Ar = 2-(C_6IH_4)

Procedure

Boronic acid (0.1 mmol) was added to the NMR tube, followed by dry $CDCl_3$ (0.7 mL). After full dissolution, 5 Å MS (4 mm from the bottom of the NMR tube after settling down) were added. Then, phenylacetic acid (0.1 mmol) was added. The product was observed by NMR and crystals suitable for X-ray analysis were obtained by vapour diffusion ($CDCl_3$/pentane). It was not possible to separate them from molecular sieves, thus the isolated yield is not reported. The maximum obtained NMR yield (^{11}B NMR) was 85%.

X = Cl, **Di-2-chlorophenylboronic B-O-B di-2-phenylacetate 11c**
Mp 152 – 160 °C. ^{1}H NMR (in mixture with boroxine and phenylacetic acid) δ 7.75 – 7.68 (m, 2H, C_{Ar}-H), 7.38 – 7.19 (m, 18H, C_{Ar}-H), 3.88 (s, 4H, CH_2). ^{13}C NMR δ 184.5, 141.4, 134.2, 133.5, 130.4, 129.8, 129.5, 129.4, 128.8, 126.2, 125.6, 42.4. ^{11}B NMR δ 5. IR ν_{max} (neat)/cm^{-1} 3348, 3087, 1607, 1579, 1520, 1405, 1318, 1259, 1206, 1131, 1105, 1071, 1030, 992, 861, 783, 726, 673, 644.
X = I, **Di-2-iodophenylboronic B-O-B di-2-phenylacetate 11d**
Mp 108 – 110 °C. ^{1}H NMR (in mixture with boroxine and phenylacetic acid) δ 7.92 – 7.87 (m, 2H, C_{Ar}-H), 7.75 – 7.69 (m, 2H, C_{Ar}-H), 7.39 – 7.26 (m, 12H, C_{Ar}-H), 7.05 – 6.98 (m, 2H, C_{Ar}-H), 3.91 (s, 4H, CH_2). ^{13}C NMR δ 184.6, 140.8, 139.9, 139.4, 134.5, 133.4, 130.1, 129.7, 128.9, 127.3, 126.9, 100.9, 42.5. ^{11}B NMR δ 5. IR ν_{max} (neat)/cm^{-1} 3283, 1698, 1581, 1460, 1416, 1344, 1241, 1124, 998, 812, 751, 700, 678.

Influence of excess of carboxylic acid and amount of 5 Å MS on the formation of complex 11c

Phenylacetic acid (0.2 mmol) and 2-Chlorophenylboronic acid (0.2 mmol [1 eq], 0.04 mmol [0.2 eq] or 0.02 mmol [0.1 eq]) were dissolved in dry $CDCl_3$ (0.7 mL). After full dissolution, 5 Å mol. sieves (2 mm from the bottom of the NMR tube after settling down) were added. The solutions were analysed by NMR.

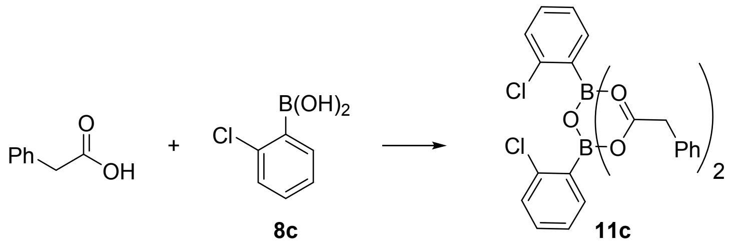

8c [^{11}**B NMR 30 ppm**]   **11c** [^{11}**B NMR 5 ppm**]

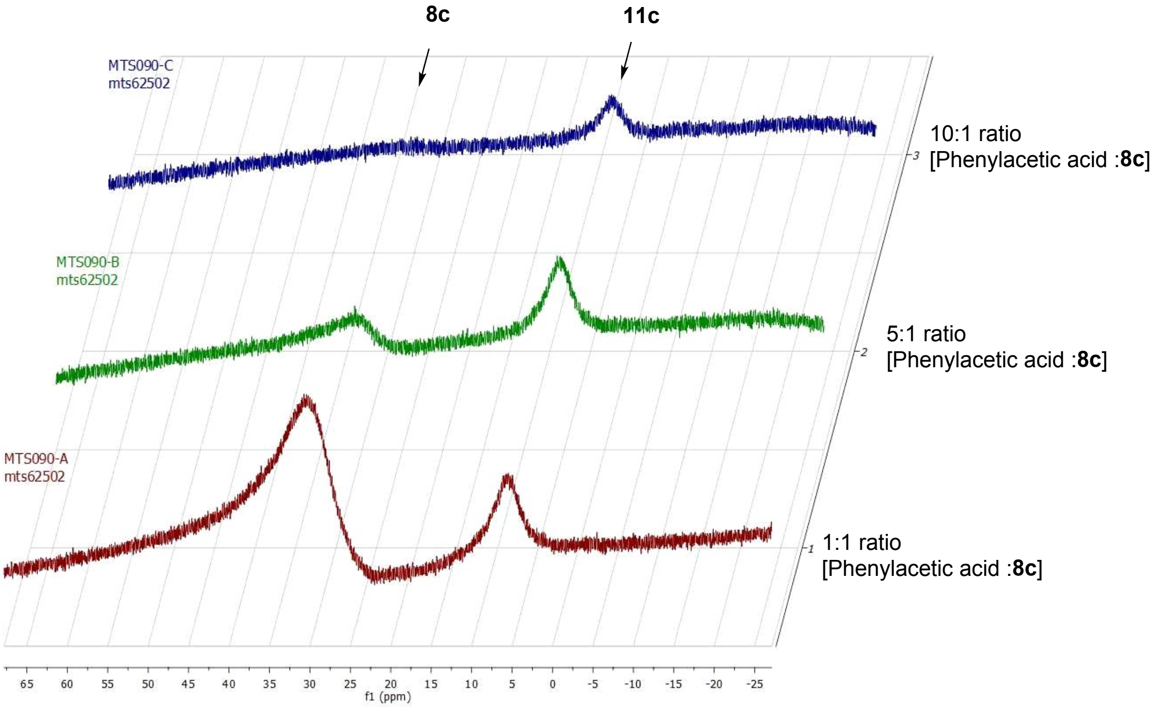

Figure E1. **Influence of excess of carboxylic acid on the formation of complex 11c**

Phenylacetic acid (0.2 mmol) and 2-chlorophenylboronic acid (0.2 mmol [1 eq] or 0.04 mmol [0.2 eq]) were dissolved in dry $CDCl_3$ (0.7 mL). After full dissolution, 5 Å mol. sieves (1, 2 or 3 mm from the bottom of the NMR tube after settling down) were added. The solutions were analysed by ^{11}B NMR.

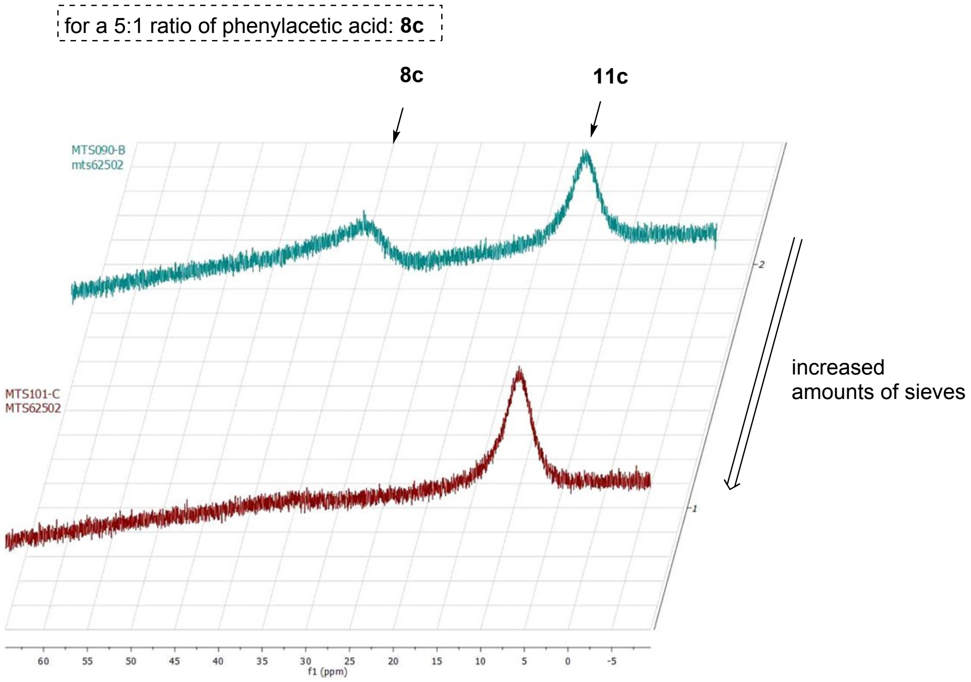

Figure E2. Influence of amount of 5 Å MS on the formation of complex 11c

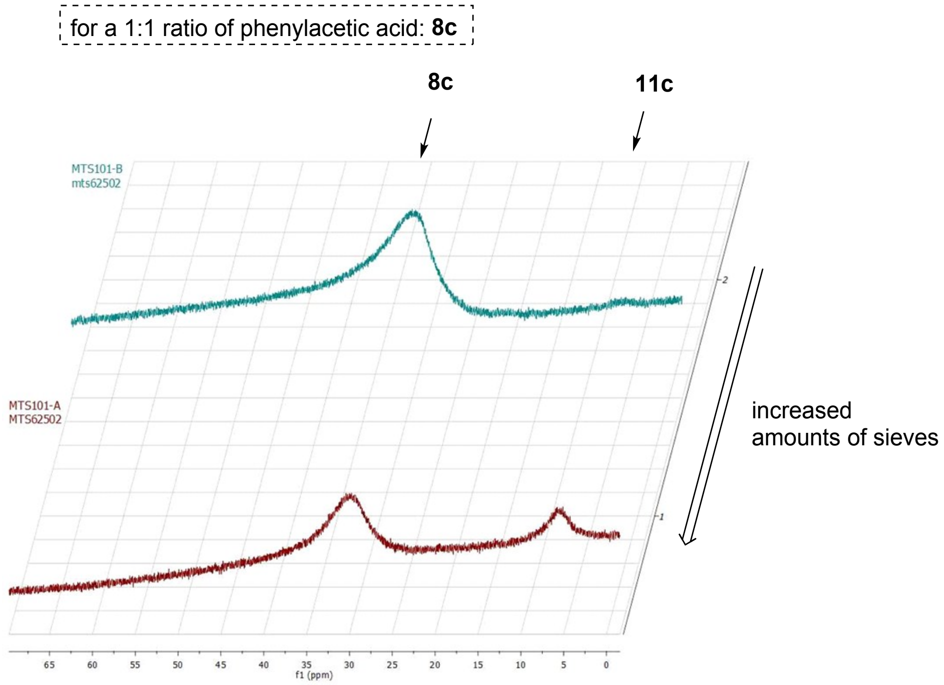

Figure E3. Influence of amount of 5 Å MS on the formation of complex 11c

2-Chlorophenylboroxine 7c generation (Fig. 7A-B and 8A-B).
2-Chlorophenylboronic acid **8c** (0.05 mmol) was added under Ar atmosphere to the NMR tube containing dry $CDCl_3$ (0.7 mL) and powdered activated 5 Å MS were added to the mixture. The product was not isolated and attempts to crystallise the product were not successful but NMR data suggested that the boroxine **7c** was formed:

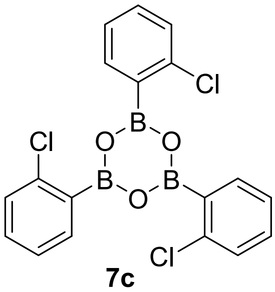

7c

Boroxine **7c**: ^{1}H NMR δ 8.33 – 8.26 (m, 3H, C_{Ar}-H), 7.54 – 7.46 (m, 6H, C_{Ar}-H), 7.44 – 7.36 (m, 3H, CH_2). ^{11}B NMR δ 29.

Interaction of B-O-B dicarboxylate complex 11c with benzylamine
2-Chlorophenylboronic acid **8c** (0.12 mmol) was added to the NMR tube, followed by dry $CDCl_3$ (0.7 mL). After full dissolution, 5 Å MS (6 mm from the bottom of the NMR tube after settling down) were added. The ^{11}B NMR suggested presence of “ate”-complex : boroxine in ~6:1 ratio. Benzylamine (10 mmol, 0.85 equiv.) was added to the mixture and it was left to stand for 2.5 hours, while being controlled by NMR. Then more benzylamine (10 mmol, 0.85 equiv.) was added to the mixture and 20 min after NMR was recorded again, and the mixture was left for 48 hours, after which last NMR analysis was run.

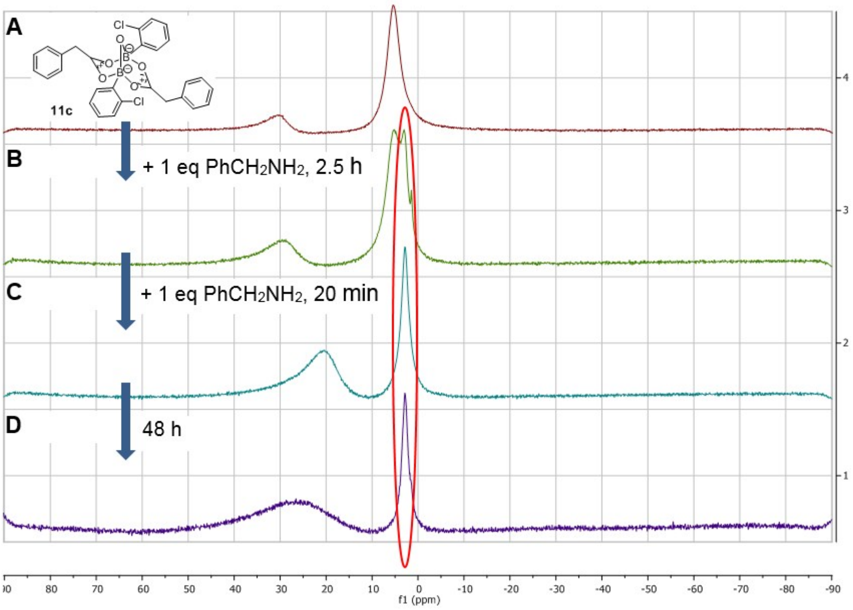

^{11}B NMRs of A): mixture of 2-chlorophenylboronic acid **8c** and phenylacetic acid in the presence of 5 Å MS resulting in equilibrium between B-O-B dicarboxylate **11a** (δ 5 ppm) and boronic species **7c** (δ 30 ppm); B): addition of 1 equivalent (per “ate”-complex **11a**) of benzylamine, 2.5 h; C): Addition of 2nd equivalent of benzylamine, 20 min; D): Same mixture over 48 h.

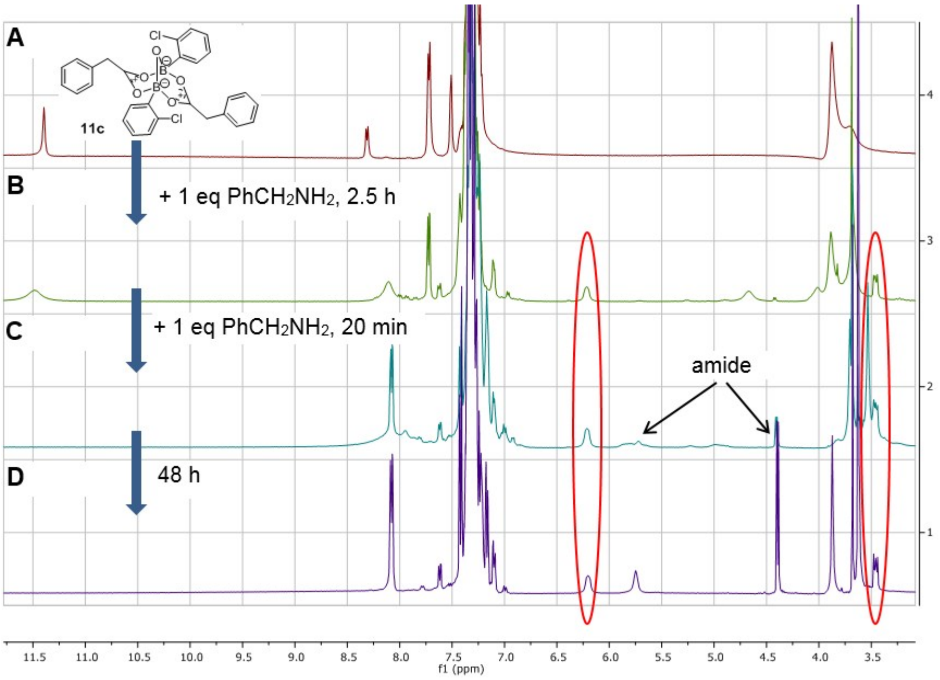

^{1}H NMRs of A): mixture of 2-chlorophenylboronic acid **8c** and phenylacetic acid in the presence of 5 Å MS resulting in equilibrium between B-O-B dicarboxylate **11c** and boronic species; B): addition of 1 equivalent (per "ate"-complex **11c**) of benzylamine, 2.5 h; C): Addition of 2nd equivalent of benzylamine, 20 min; D): Same mixture over 48 h.

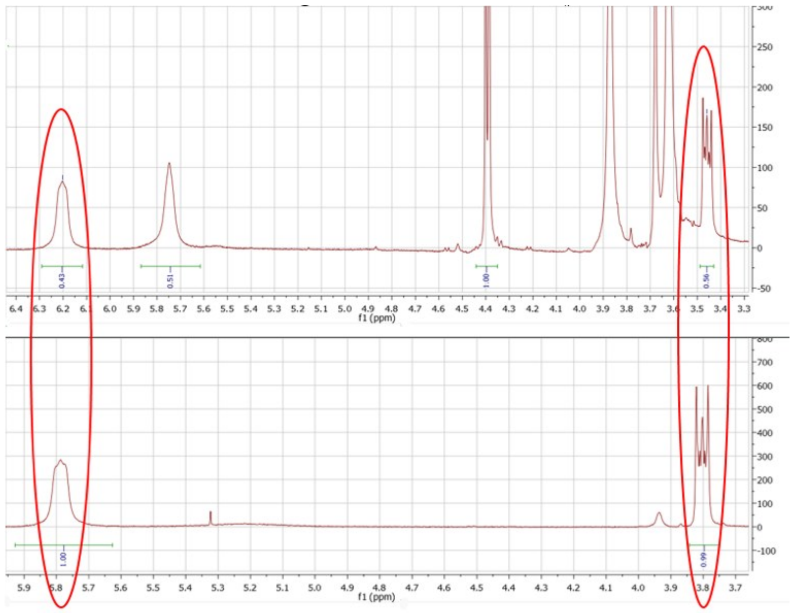

^{1}H NMR spectra of: (top) the reaction of complex **11c** with 1 eq benzylamine after 48 h; (bottom) borinic aminocarboxylate complex **5c** (note that all borinic aminocarboxylates **5a-d** look very similar in terms of 2 signals highlighted). Note the identical multiplicity of 2 signals. The chemical shifts are different, as would be expected due to the difference in Lewis acidity between borinic and boronic acids.

Effects of the order of addition of acid / amine / boronic acids for amidation

Procedure:

A 6 mL vial equipped with a stirrer was filled with acid or amine (0.5 mmol), 2-Cl phenylboronic acid (0.1 mmol) and a stock solution of 1,4 dimethoxybenzene (0.125 mmol) in 1 mL $CDCl_3$. The system was flushed with Argon, and further 2 mL of $CDCl_3$ were added. Freshly prepared 5 Å mol. sieves (~1 g) were quickly added. An aliquot (0.2 mL) of reaction mixture was taken after 15 min of stirring, diluted in 0.4 mL $CDCl_3$ and analysed by NMR.

The corresponding acid or amine (0.5 mmol) reaction component was then added and aliquots taken at regular time intervals (0.3 mL each at 0h, 4h, and 24 h).

Note: Heterogeneous amide bond forming reactions of this sort require stirring. Reactions conducted in NMR tube only have very slow conversion to amide in comparison.

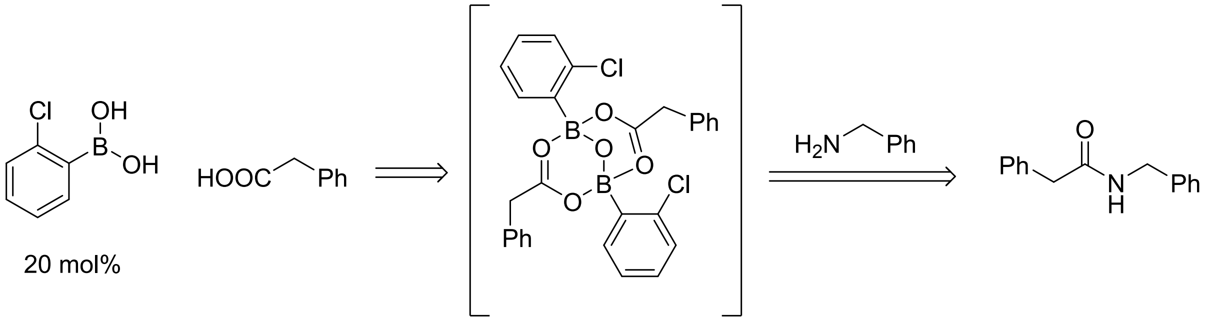

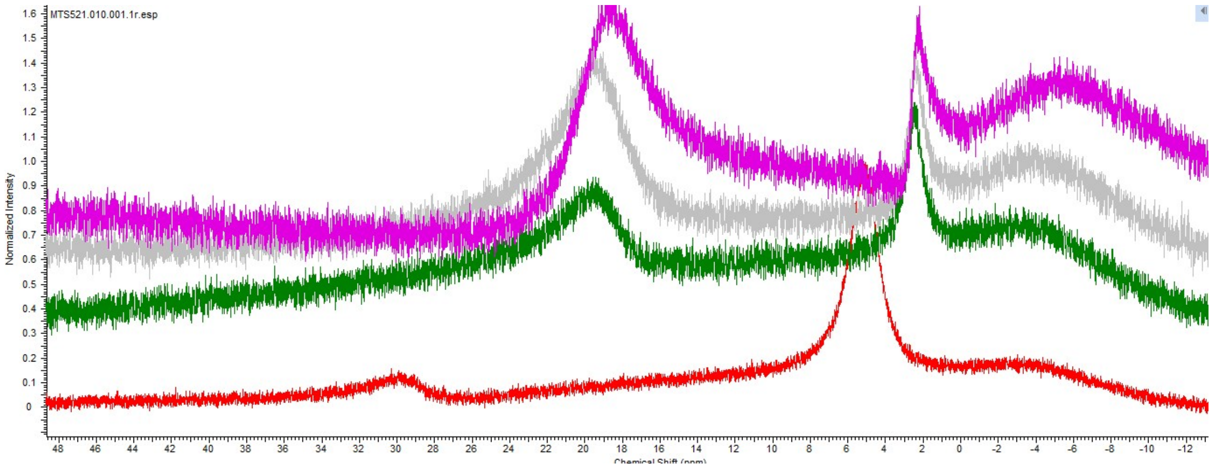

time	Yield %	Spectrum (bottom up)
pre	-	(*BNMR s1*)
2	**>95**	(*BNMR s2*)
4	**>95**	(*BNMR s3*)
8	**-**	(*BNMR s4*)

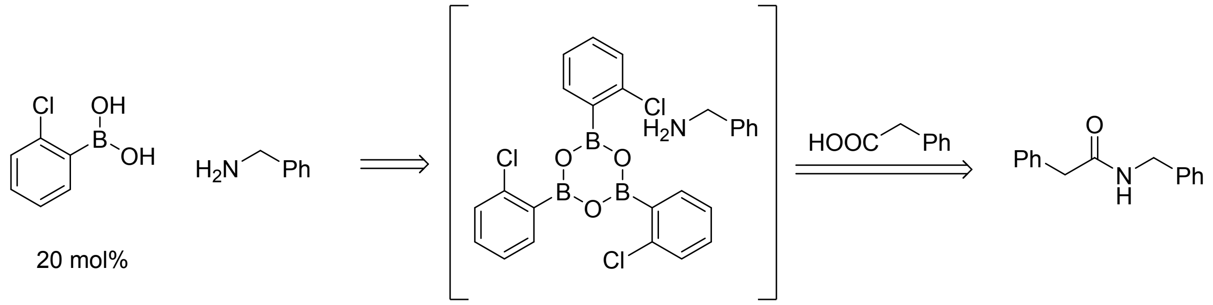

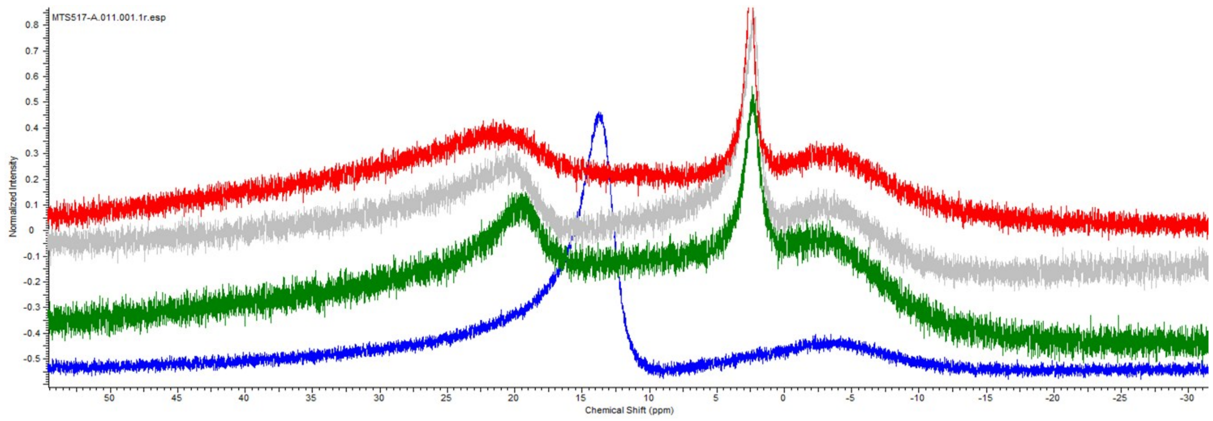

time	Yield %	Spectrum (bottom up)
pre	-	(*BNMR s1*)
2	**50**	(*BNMR s2*)
4	**85**	(*BNMR s3*)
8	-	(*BNMR s4*)

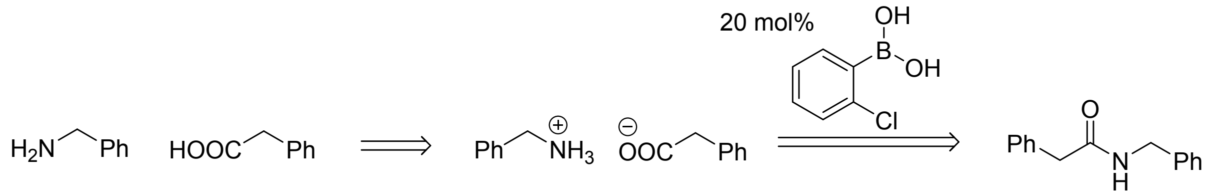

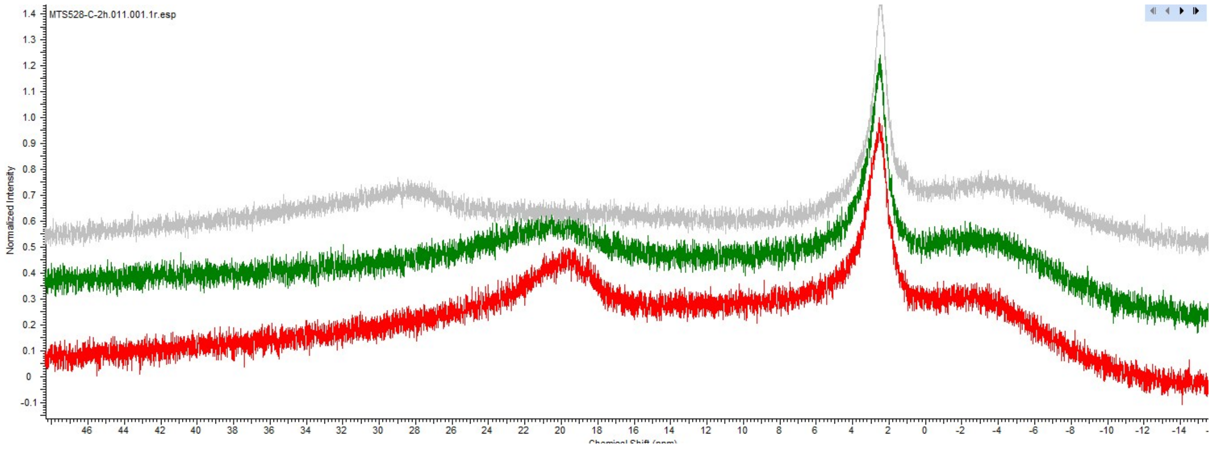

time	Yield %	Spectrum (bottom up)
pre	-	-
2	**53**	(*BNMR s1*)
4	**84**	(*BNMR s2*)
8	-	(*BNMR s3*)

2.D. Interaction of B_2O_3 and $B(OH)_3$ with acids and amines

Interaction of B_2O_3 and $B(OH)_3$ with phenylacetic acid, benzylamine and 5 Å MS
Boric acid or B_2O_3 (0.05 mmol) was added to the NMR tube, followed by dry $CDCl_3$ (0.7 mL) and benzylamine (0.1 mmol). Full dissolution was not achieved. After this ^{11}B NMR was recorded, then phenylacetic acid (0.1 mmol) was added and ^{11}B NMR was run again. Then 5 Å MS (4 mm from the bottom of the NMR tube after settling down) were added and the mixture was left to stand for 3 min before running final ^{11}B NMR analysis.

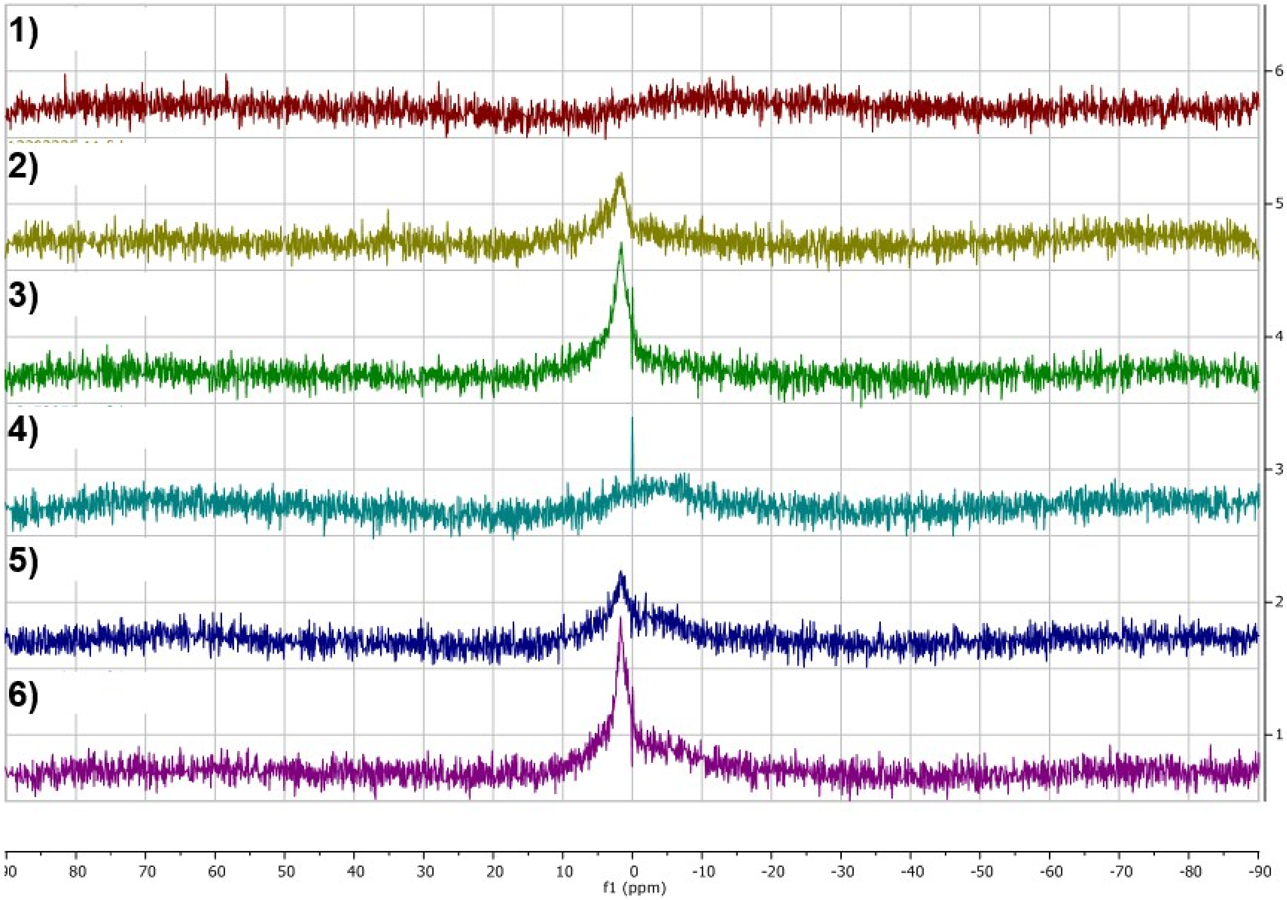

Figure E4. Top to bottom:

1. $CDCl_3$, $B(OH)_3$ + benzylamine 1:1 – no dissolution
2. + 1 eq. $PhCH_2COOH$
3. + 5 Å MS
4. Separate experiment: $CDCl_3$, B_2O_3 + benzylamine 1:1 – no solubilization
5. + 1 eq $PhCH_2COOH$
6. + 5 Å MS

2.E. Kinetic studies

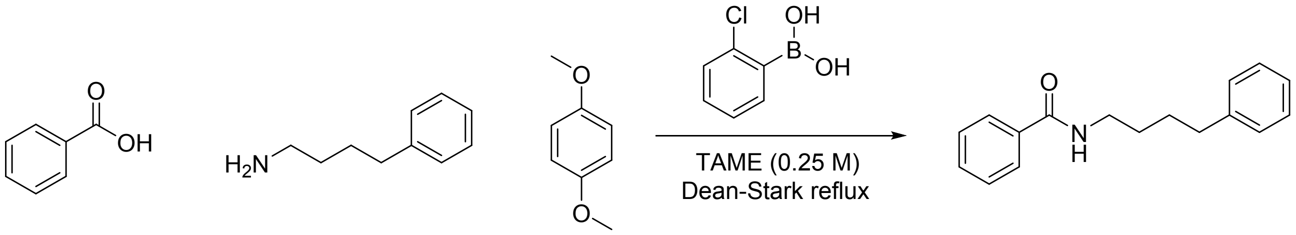

Procedure:
A stirred solution of amine (5.0-8.0 mmol), carboxylic acid (5.0-8.0 mmol), 2-Cl phenylboronic acid (0.125-0.5 mmol) and dimethoxybenzene (1.25 mmol) in TAME (20 mL) with a Dean-Stark (side-arm filled with TAME) was heated to reflux (bp, 86 °C). Aliquots (0.3 mL) were taken at regular time intervals and quenched in 0.6 mL of DMSO-d_6. TAME was removed under high vacuum at RT for 10 min and resulting solution directly analysed by ^{1}H NMR.
For consistency, spectra were phased using the '*auto simple*' function (under '*phase*' tab) and baseline corrected using the '*auto*' function (under '*baseline*' tab) in ACD/NMR Processor. Yield of amide was calculated with CH_2 signal alpha to the amidic NH (3.28 ppm, q, J = 6.6 Hz on 400 mHz) against internal standard, dimethoxybenzene.

<u>Kinetics of catalyst</u>

All data analysed with the Bures method: Assuming the catalyst is not significantly deactivated during the course of the reaction, the order with respect to boronic acid catalyst can be determined by plotting [product] against t[cat]$^\alpha$, where $^\alpha$ is the order of the reaction

<u>For catalyst loadings of 2.5-5.0 mol%, order in catalyst is ~ 1</u>

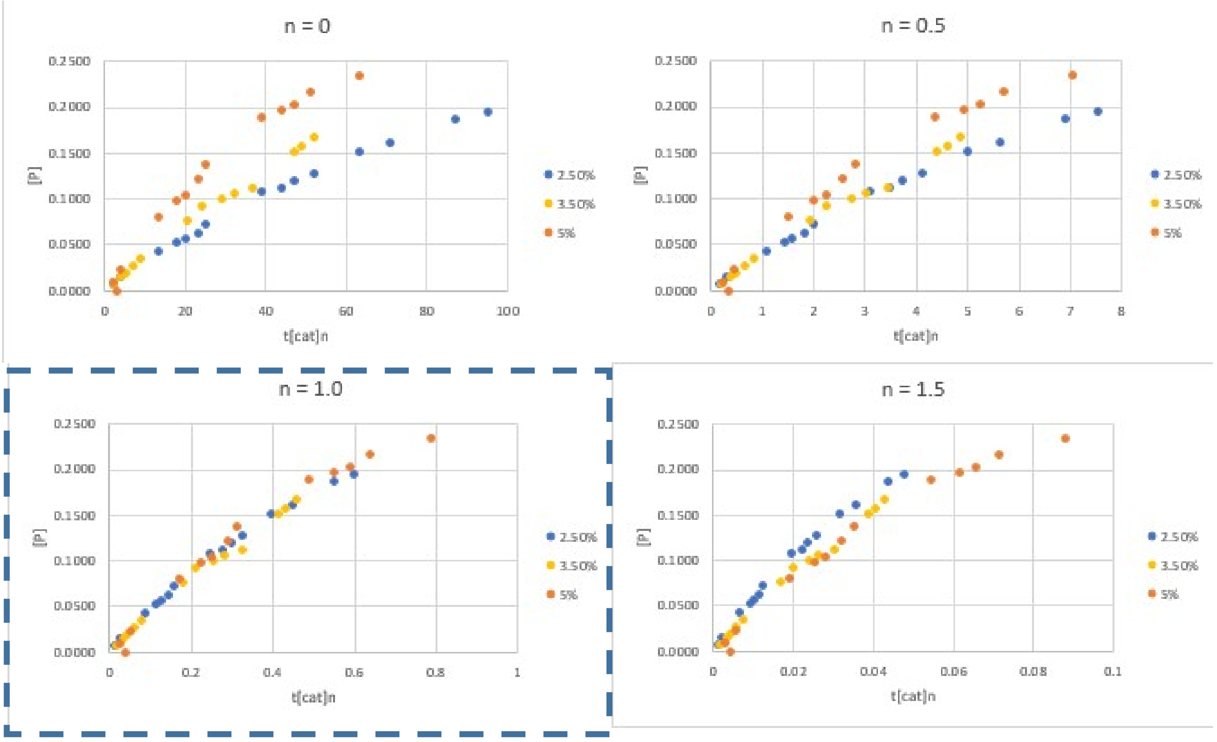

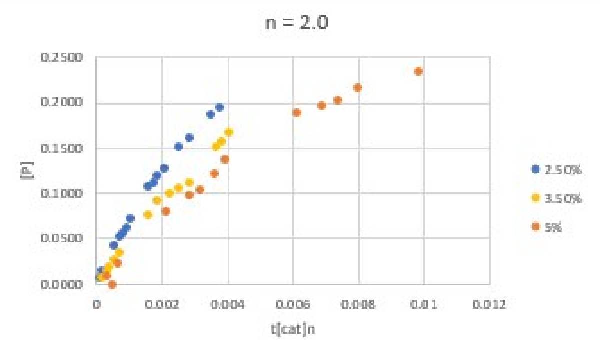

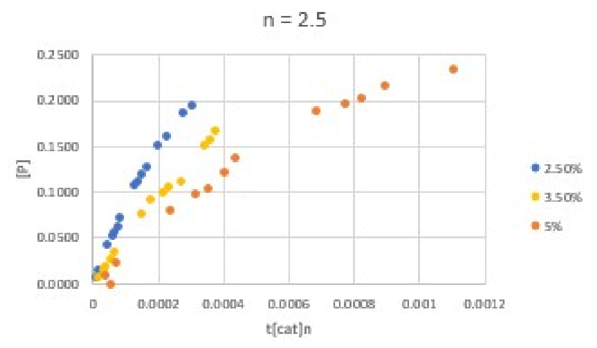

For catalyst loadings from 5.0-10 mol%, order in catalyst is ~ 0.3

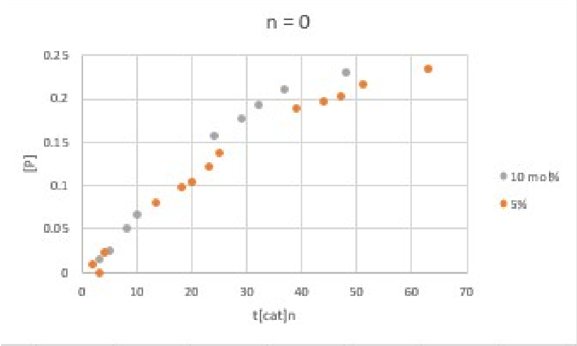

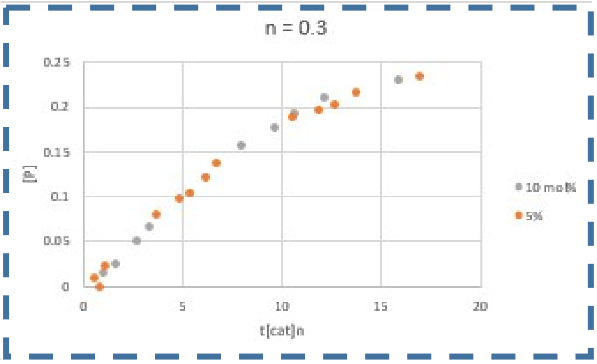

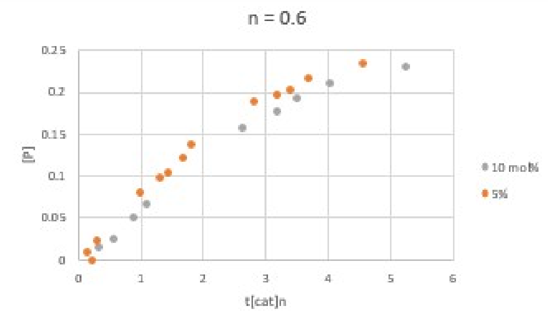

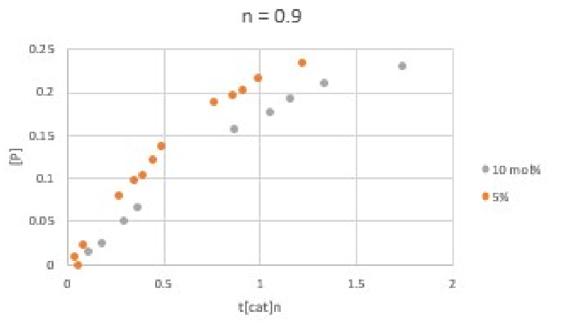

Raw data for catalyst studies:

Reactions run with equimolar acid and amine with varying equivalents of catalyst

Time (h)	*2.5 mol%* ***yield***	*5.0 mol %* ***yield***
2	3%	4%
4	6%	9%
13.5	17%	32%
18	21%	39%
20	23%	42%
23	25%	49%
25	29%	55%
39	43%	77%
44	45%	79%
47	48%	81%
51		87%
51.75	51%	
63	61%	94%
71	65%	95%
87	75%	
95	78%	

Time (h)	*3.5 mol%* ***yield***	*10 mol%* ***yield***
2	3%	
3		6%
4	6%	
5	8%	10%
7	11%	
8		20%
9	14%	
10		27%
20.5	31%	
24	37%	63%
27	40%	
29	42.50%	71%
32	45%	77%
36.75		84%
44	61%	
48		92%
49	63%	
51.75	67%	
54		94%

ACID studies

Raw data for determination of order of Acid

For determination of the order in carboxylic acid, the reaction was run with 3.5 mol%. Reaction were run with 1.0 eq 1.2 eq and 1.4 eq of benzoic acid. A plot of the concentration of product vs. time is shown below:

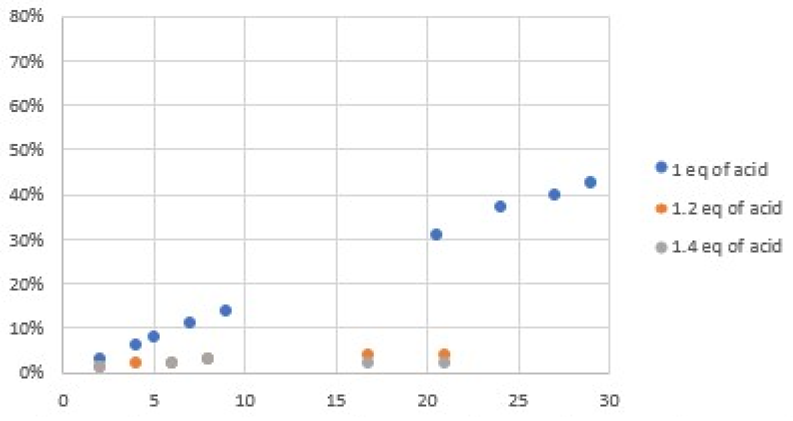

Excess acid shuts down reaction at 3.5 mol% catalyst

The reaction with 1.4 eq of acid was run with 3.5 mol%, 5 mol% and 10 mol% catalyst. The results are shown on the graph below:

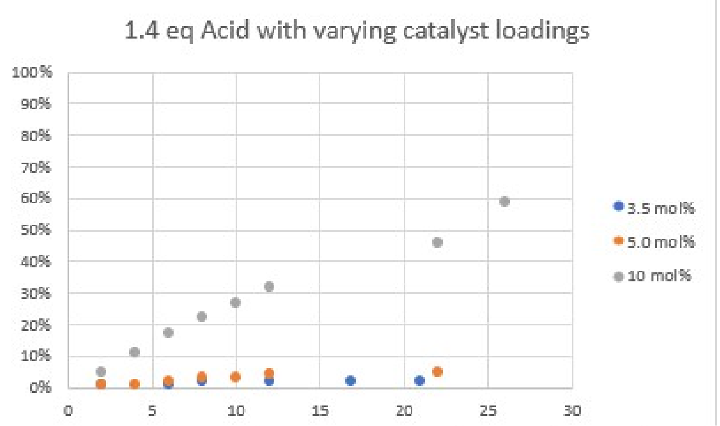

<u>Excess acid shuts down reaction at 3.5 mol% and 5 mol% but not at 10 mol%</u>

Next we looked at the effects of varying amounts of excess acid at 10 mol% catalyst loading. Reactions were run with 1.0 eq 1.2 eq,1.4 eq and 1.6 eq of benzoic acid. A plot of the concentration of product vs. time is shown below:

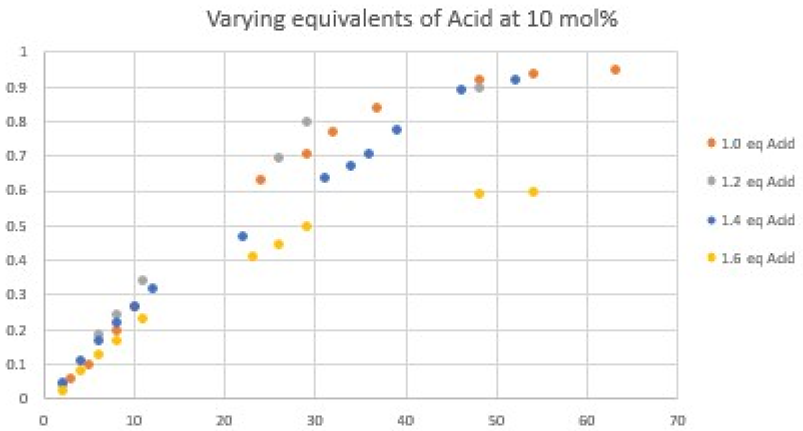

<u>1.2 eq of acid has a very similar rate of reaction to 1.0 eq of acid</u>

<u>1.4 and 1.6 eq of acid slow down the reaction relative to 1.0 eq of acid</u>

Kinetic analysis for order determination

The graphical method developed by Burés was used to plot [Product] against the variable time scale normalized in carboxylic acid ($\sum$[Acid]$^{\alpha}$□t). When α is the correct order in [Acid] the traces will overlay.

$$\int_{t=0}^{t=n} [\mathrm{A}]^{\alpha} \mathrm{d}t = \sum_{i=1}^{n} \left(\frac{[\mathrm{A}]_i + [\mathrm{A}]_{i-1}}{2} \right)^{\alpha} (t_i - t_{i-1})$$

<u>The order for 1.4 and 1.6 eq of acid at 10 mol% follows a negative order of reaction ~ -0.5.</u>

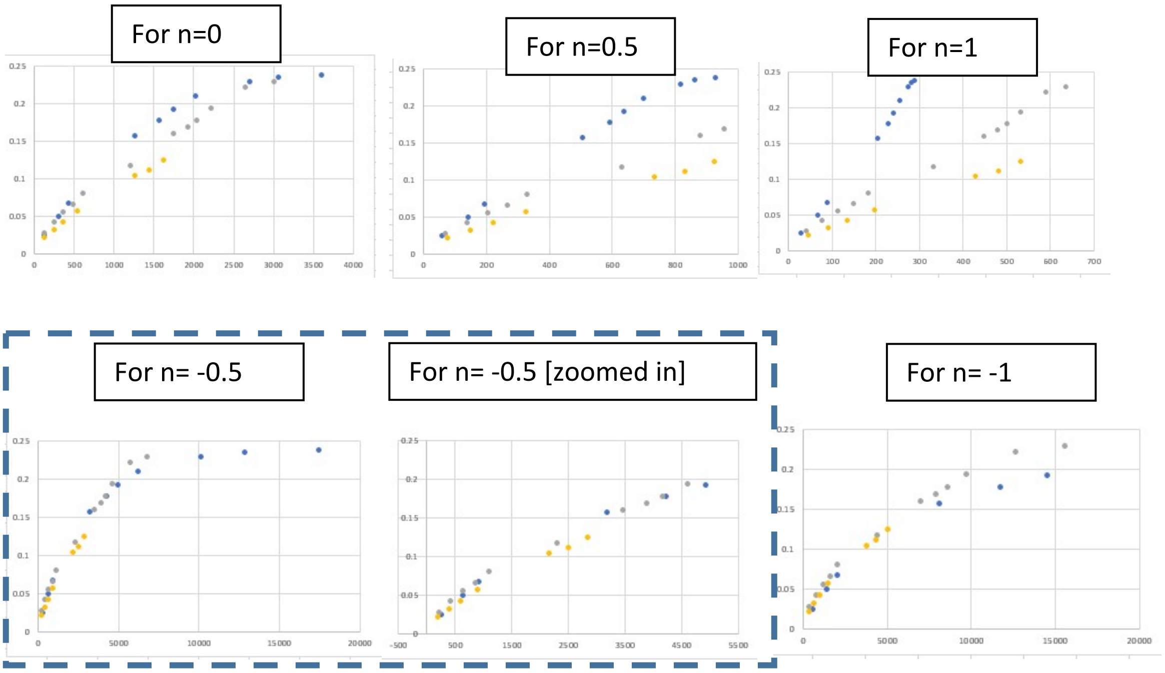

Raw data for acid studies:

Reactions run with 10 mol%, with varying equivalents of acid

Time	1.0 eq acid yield	*Time*	1.2 eq acid yield	*Time*	1.4 eq acid yield	*Time*	1.6 eq acid yield
3	6%	*2*	4%	*2*	5%	*2*	2.50%
5	10%	*6*	18.50%	*4*	11%	*4*	8.50%
8	20%	*8*	24.50%	*6*	17%	*6*	13%
10	27%	*11*	34%	*8*	22%	*8*	17%
24	63%	*26*	69.50%	*10*	26.50%	*11*	23%
29	71%	*29*	80%	*12*	32%	*23*	41.50%
32	77%	*48*	90%	*22*	47%	*26*	44.50%
36.75	84%	*54*	92%	*31*	64%	*29*	50%
48	92%			*34*	67.50%	*48*	59%
54	94%			*36*	71%	*54*	60%
63	95%			*39*	78%		
				46	89%		
				52	92%		

<u>AMINE studies</u>

For determination of the order in amine, the reaction was run with 3.5 mol%. Reaction were run with 1.0 eq 1.2 eq and 1.4 eq of phenylbutylamine.

The graphical method developed by Burés was used to plot [Product] against the variable time scale normalized in amine ($\sum[\text{Amine}]^{\alpha}$□t). When α is the correct order in [Amine] the traces will overlay.

For a catalyst loading of 3.5 mol%, order in amine is somewhere between ~0.3-0.6

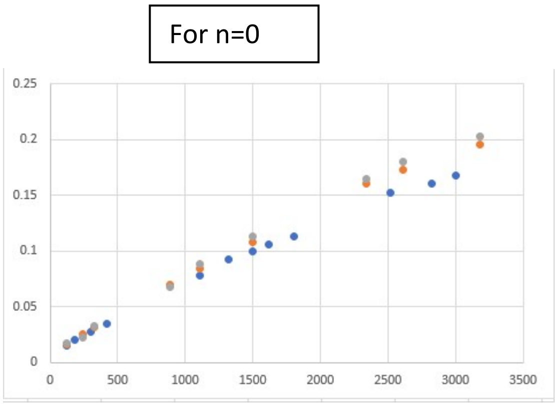

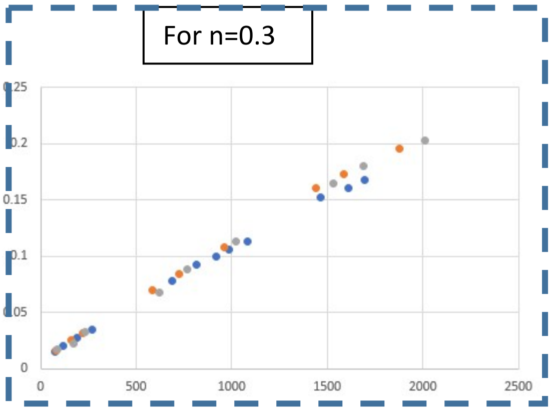

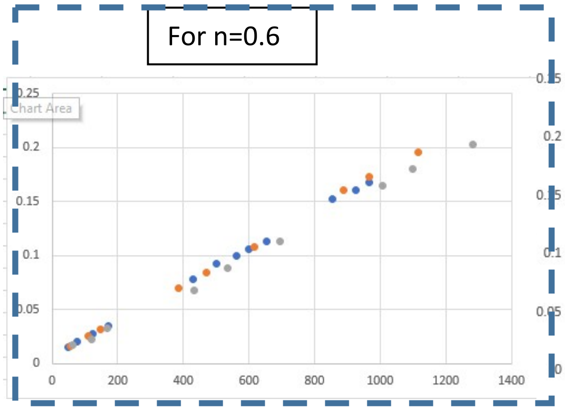

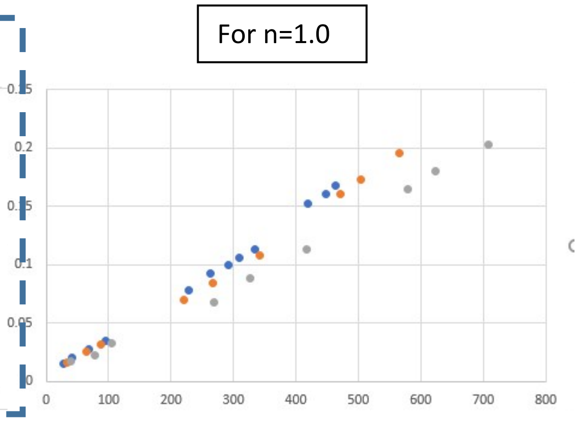

Raw data for acid studies:

Reactions run with 3.5 mol%, with varying equivalents of amine

Time (h)	*1.0 eq amine yield*
2	3%
4	6%
5	8%
7	11%
9	14%
20.5	31%
24	37%
27	40%
29	42.50%
32	45%
44	61%
49	63%
51.75	67%

Time (h)	*1.2 eq amine yield*	*1.4 eq amine yield*
2	3%	3%
4	6.50%	7%
6	10%	9%
7.5	12.50%	13%
16.8	28%	27%
20.5	33.50%	35.50%
27	43%	45%
41	-	66%
45.5	69%	72%
55	78%	81%

When comparing 1.4 eq of amine at 3.5 mol% and 10 mol% boronic acid we also see a difference.

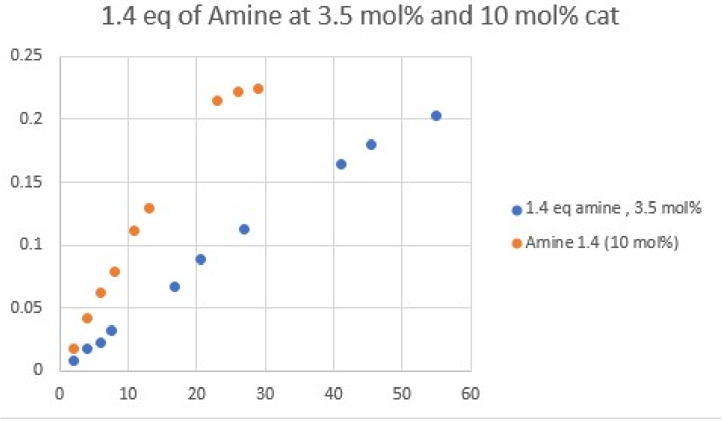

<u>With excess amine (1.4 eq) the order of catalyst at 3.5 -10 mol% is now ~1 (contrasting the 0.3 order with 1.0 eq of amine between 5-10 mol%.</u>

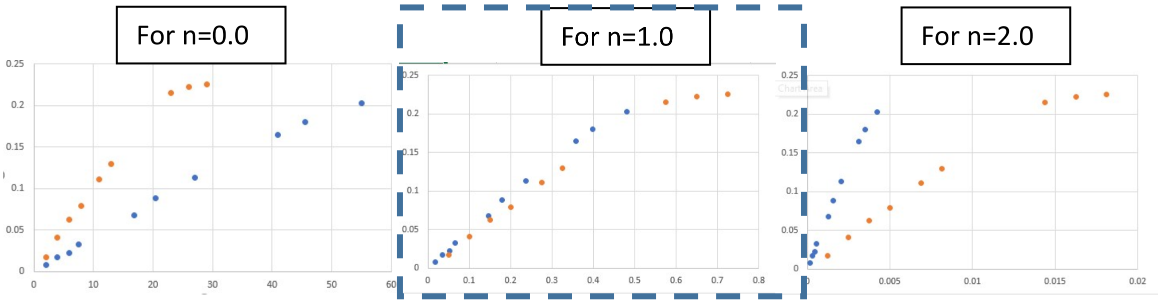

3. Tabulated summary of spectroscopic data

Data on carbonyl C=O IR stretches, ^{13}C NMR of carbonyl carbon, C=O, C-O and B-O bond lengths and ^{11}B NMRs of selected compounds isolated during this work

Note: Full NMR data including all FIDs can be obtained from a data repository using the DOI: 10.14469/hpc/2247

Structure	^{11}B NMR, δ	^{13}C NMR (C=O), δ	IR (C=O), cm^{-1}	B-O, Å	C=O or "C=O", Å	C-O, Å
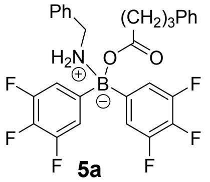 5a	1.4	178.8	1677	1.49	1.214	1.323
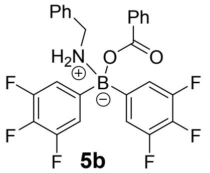 5b	1.9	170.9	1665	1.491	1.219	1.324
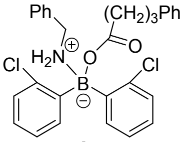 5d	2.5	178.0	1678	1.499	1.216	1.319
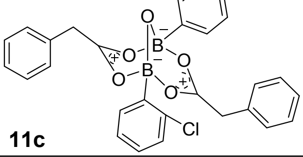 11c	5.5	184.7	1698	1.550; 1.584	1.256	1.276
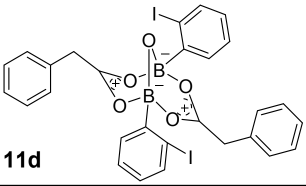 11d	5.3	184.5	1705	1.548; 1.571	1.27	1.276
$PhCH_2COOH$	-	177.2	1715		-	-

4. X-ray crystallographic information

X-ray diffraction experiments for **5d** and **ESI1** were performed on an Agilent Xcalibur κ-diffractometer with a Sapphire-3 CCD detector, using graphite monochromated Mo-*K*α radiation from a Enhance (Mo) fine-focus X-ray source, for all other crystals on a Bruker D8 Venture 3-circle diffractometer with a PHOTON 100 CMOS area detector, using Mo-*K*α or (for **5a** and α-**7a** at 220 K) Cu-*K*α radiation from Incoatec IμS microsources with focussing mirrors. The crystals were cooled using Cryostream (Oxford Cryosystems) open-flow N_2 gas cryostats. Absorption corrections were carried out by semi-empirical method based on Laue equivalents and multiple scans or (for α-**7a**) by numerical integration based on crystal face-indexing, using SADABS program.[3] Structure β-**7a** was solved by iterative method using OLEX2.SOLVE program,[4] **11d** by Superflip method,[5] all other structures by direct methods using SHELXS programs.[6] All structures were refined by full-matrix least squares using SHELXL[7] and OLEX2[8] software. Crystal data and experimental details are listed in Tables S1-S4. Crystal **4b** was an inversion twin with a 0.6(1):0.4(1) component ratio. The residual electron density and anomalous scattering of **ESI2** revealed a *ca.* 5% replacement of Cl(1) atom with Br, presumably coming from Grignard reagent, the absolute structure was determined from the Flack parameter, x=–0.02(3) by classical fit to all intensities[9] and –0.027(9) from 2145 selected quotients (Parsons' method[10]).

The asymmetric unit of **4b** comprises two host molecules (with a flipping disorder of one triflurophenyl ring) and one intensely disordered deuterochloroform molecule. **5b** shows a flipping disorder of the benzoate phenyl ring. **5c** crystallises as a 1:1 dichloromethane solvate. The asymmetric unit of **10c** comprises two molecular pairs. **10d** shows librational disorder in two benzoyl phenyl rings. Crystals of **11c** and **11d** are isomorphous. The asymmetric unit of **S1** comprises two molecules; that of **ESI3** comprises three, two of which are mutually related by an approximate pseudo-translation *a*/3, and the third is linked to them by the same pseudo-translation *plus* an inversion about its own centroid.

Compound **7a** was studied in two polymorphs. Monoclinic α-form (space group *C*2/*c*) crystallised from deuterochloroform, was studied at room temperature and 220 K, which gave practically identical structure. On cooling below 210 K it undergoes a phase transition, turning polycrystalline. The orthorhombic β-form (space group *Pbcn*) was obtained in an independent synthesis and recrystallized from chloroform/pentane; it does not show any phase transitions from room temperature to 120 K. In both polymorphs the molecule lies astride a twofold axis, the central boroxine ring is planar, in α-**7a** arene ring *i* is inclined to it by 12.0° and two (equivalent) rings *ii* by 4.3°, in β-**7a** the corresponding angles are 4.3° and 5.8°.

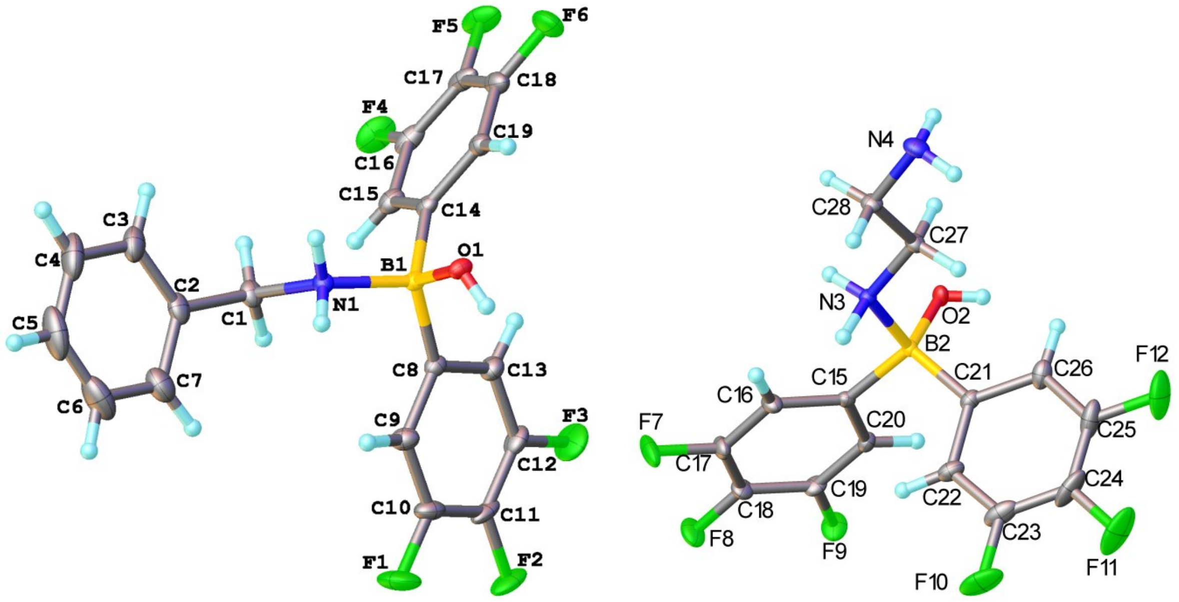

Figure X1. X-ray molecular structure and atomic numbering scheme in **4a** (left) and **4b** (right)

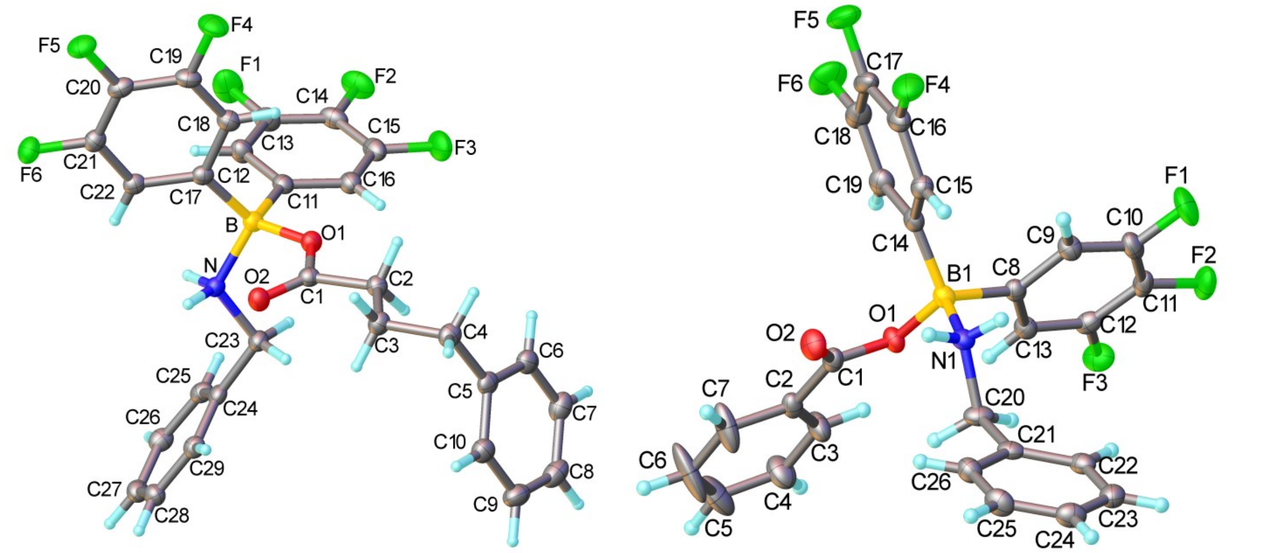

Figure X2. X-ray molecular structure and atomic numbering scheme in **5a** (left) and **5b** (right). Here and elsewhere atomic displacement ellipsoids are drawn at the 50% probability level

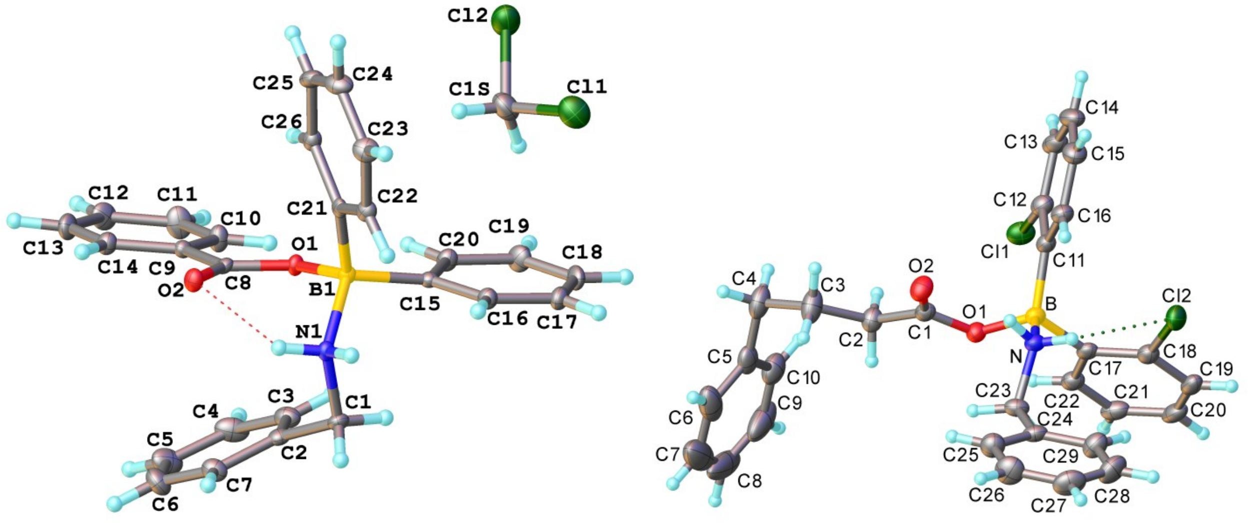

Figure X3. X-ray molecular structure and atomic numbering scheme in **5c** (left) and **5d** (right).

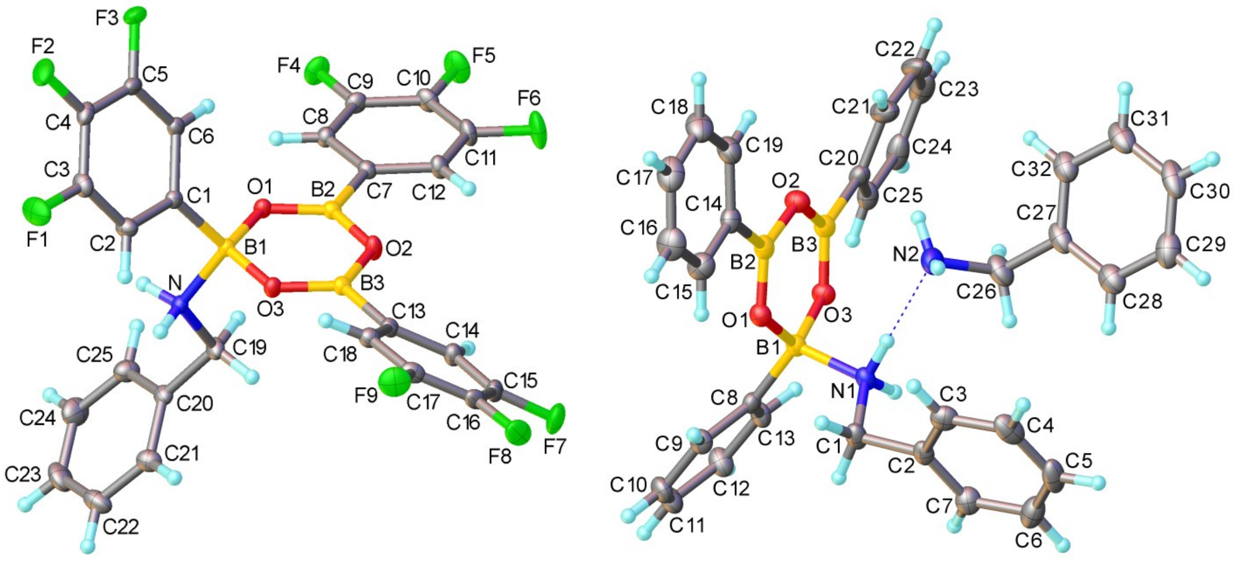

Figure X4. X-ray molecular structure and atomic numbering scheme in **10a** (left) and **10c** (right)

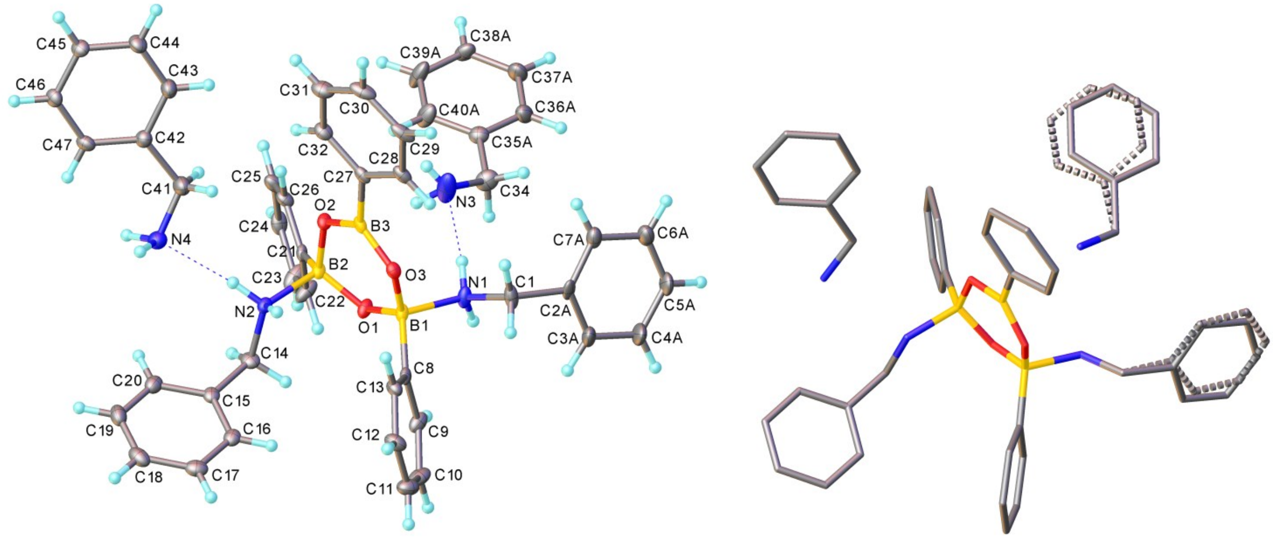

Figure X5. X-ray molecular structure atomic numbering scheme in **10d** (left, major conformation) and disorder in the crystal (right)

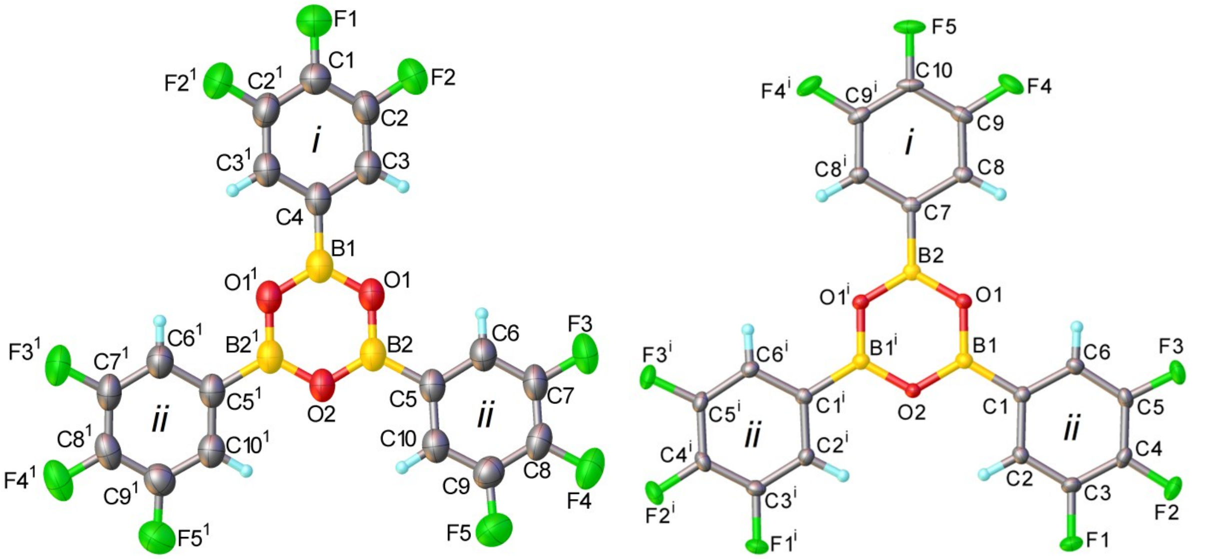

Figure X6. X-ray molecular structure of **7a** in α-polymorph at 220 K (left) and β-polymorph at 120 K (right). Primed atoms are generated by the twofold axis

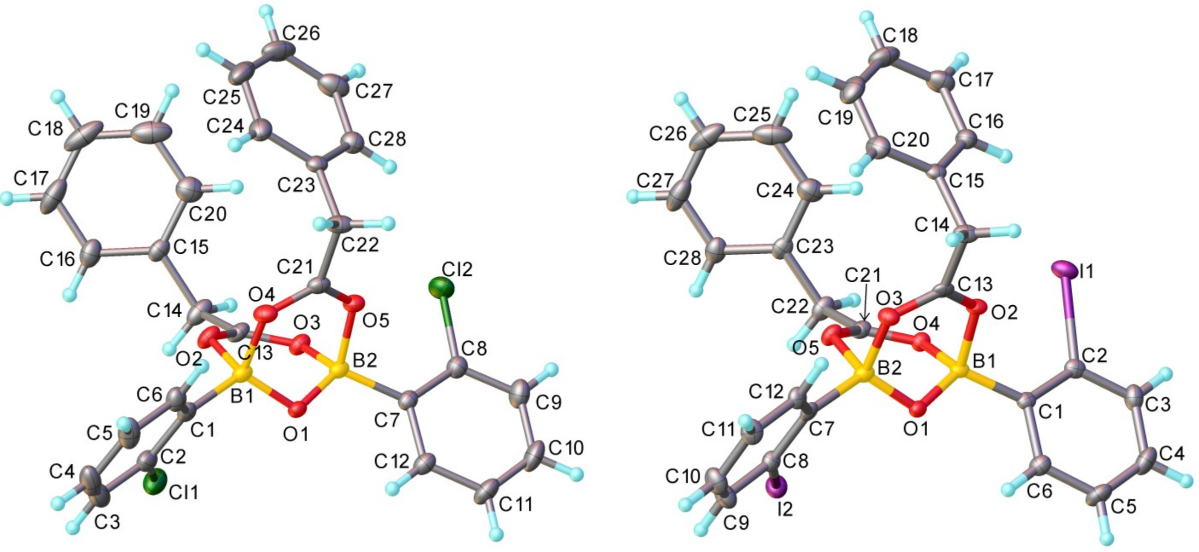

Figure X7. X-ray molecular structure and atomic numbering scheme in **11c** (left) and **11d** (right)

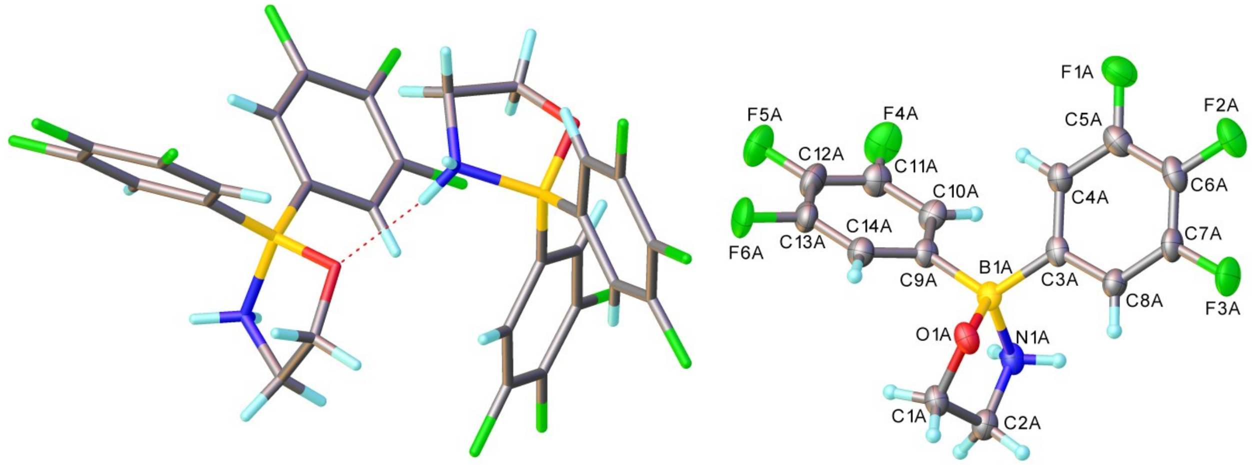

Figure X8. Two independent molecules in the structure of **ESI1** (left) and molecule A (right). Atomic numbering scheme in molecule B is analogous

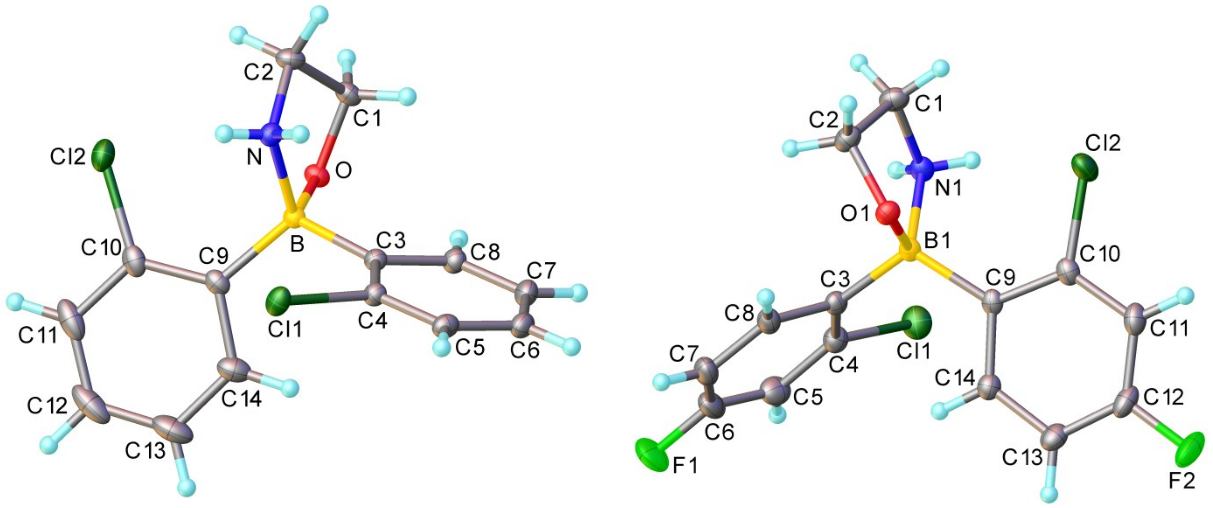

Figure X9. X-ray molecular structure and atomic numbering scheme in **ESI2** (left) and **ESI3** (right)

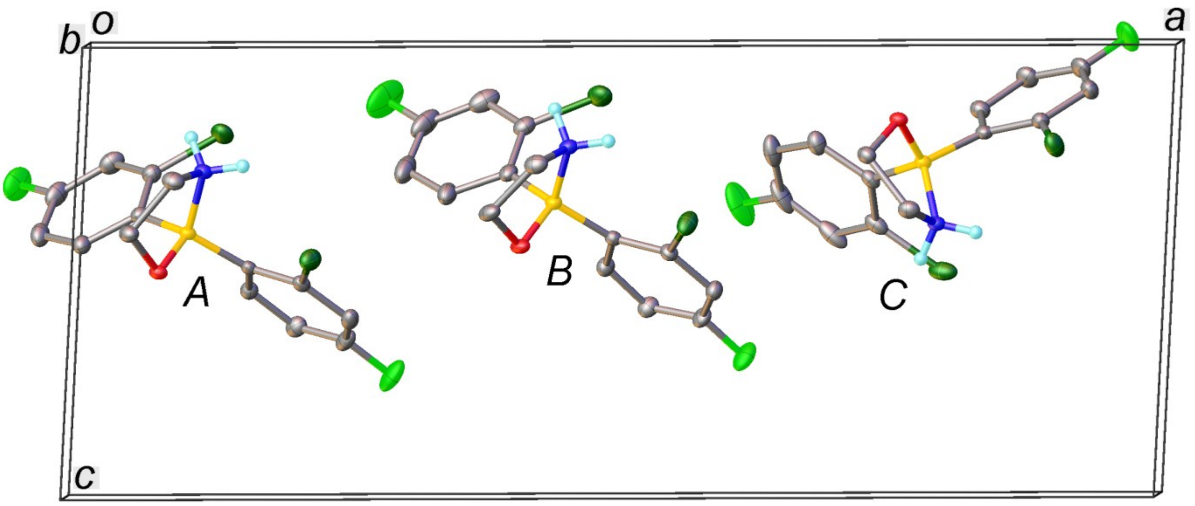

Figure X10. Three independent molecules in the unit cell of **ESI3**

Table S1. Crystal data of compounds **4** and **5**

Compound	**4a**	**4b**	**5a**	**5b**	**5c**	**5d**
Depository code	15srv002	15srv104	14srv256	15srv006	15srv050	16srv084
CCDC deposition no.	1551614	1551615	1551616	1551617	1551618	1551619
Formula	$C_{19}H_{14}BF_6NO$	$C_{14}H_{13}BF_6N_2O$ ·½ $CDCl_3$	$C_{29}H_{24}BF_6NO_2$	$C_{26}H_{18}BF_6NO_2$	$C_{26}H_{24}BNO_2$ ·CH_2Cl_2	$C_{29}H_{28}BCl_2NO_2$
$D_{calc.}$/ g cm^{-3}	1.509	1.607	1.396	1.473	1.330	1.274
μ/mm^{-1}	0.14	0.37	1.00	0.13	0.30	0.27
Formula Weight	397.12	410.26	543.30	501.22	478.20	504.23
T/K	120	120	120	120	120	120
Crystal System	triclinic	monoclinic	triclinic	monoclinic	monoclinic	triclinic
Space Group	*P*-1 (# 2)	*Pc* (# 7)	*P*-1 (# 2)	$P2_1/n$ (# 14)	$P2_1/n$ (# 14)	*P*-1 (# 2)
a/Å	9.03430(10)	11.7500(5)	10.0140(3)	10.1019(4)	14.1158(3)	9.2006(2)
b/Å	10.2063(2)	11.0269(4)	11.7859(4)	11.7656(5)	8.9604(2)	11.4729(4)
c/Å	11.0348(2)	13.1973(5)	23.0748(7)	19.5670(9)	18.8756(4)	13.1121(4)
α/°	66.3381(18)	90	103.8183(8)	90	90	101.570(3)
β/°	70.1856(17)	97.3307(14)	98.0649(10)	103.6902(15)	90.0788(17)	99.773(2)
γ/°	78.1262(19)	90	96.9376(9)	90	90	98.085(3)
V/Å^3	873.89(3)	1695.95(11)	2584.47(14)	2259.56(17)	2387.44(9)	1314.23(7)
Z	2	4	4	4	4	2
λ/Å	0.71073	0.71073	1.54184	0.71073	0.71073	0.71073
θ_{max} /°	29	29.3	66	27.5	29	27.5
measured refl.	18051	35272	21248	29313	35929	18221
unique refl.	4649	9254	8496	5189	6345	6009
refls with I>2σ(I)	3848	8294	7740	4345	4421	4561
R_{int} , %	3.2	2.9	5.7	2.8	6.9	4.6
Parameters/restraints	309, 0	553, 57	719, 0	346, 74	402, 0	316, 0
Δρ max/min, eÅ^{-3}	0.42, -0.28	0.50, -0.63	0.39, -0.26	0.31, -0.22	0.35, -0.39	0.51, -0.37
R_1, wR_2 (all data), %	4.3, 10.7	5.4, 12.7	6.1, 16.3	4.7, 9.8	8.8, 11.0	7.0, 12.6
R_1, wR_2 [I>2σ(I)], %	4.3, 10.0	4.6, 12.1	5.8, 15.8	3.7, 9.1	4.9, 9.7	4.8, 11.2

Table S2. Crystal data of compound **7a**

**Compound**	α-**7a**	α-**7a**	β-**7a**
Depository code	14srv091	14srv233	16srv413
CCDC deposition no	1563948	1563949	1563950
Formula	$C_{18}H_6B_3F_9O_3$	$C_{18}H_6B_3F_9O_3$	$C_{18}H_6B_3F_9O_3$
$D_{calc.}$/ g cm^{-3}	1.719	1.739	1.761
μ/mm^{-1}	0.17	1.58	0.18
Formula Weight	473.66	473.66	473.66
T/K	293	220	120
Crystal System	monoclinic	monoclinic	orthorhombic
Space Group	*C*2/*c* (#15)	*C*2/*c* (#15)	*Pbcn* (# 60)
a/Å	12.8358(9)	12.8086(4)	17.083(4)
b/Å	12.6583(9)	12.6262(4)	13.093(3)
c/Å	12.8851(9)	12.8625(5)	7.990(2)
β/°	119.060(2)	119.598(2)	90
V/Å^3	1830.0(2)	1808.74(12)	1787.0(8)
Z	4	4	4
λ/Å	0.71073	1.54184	0.71073
θ_{max} /°	25	75.3	28
measured refl.	9411	6438	22891
unique refl.	1619	1790	2162
refls with I>2σ(I)	1161	1418	1466
R_{int} , %	2.9	5.4	7.9
Parameters	152	152	164
Δρ max/min, eÅ^{-3}	0.18, -0.13	0.40, -0.27	0.31, -0.30
R_1, wR_2 (all data), %	5.9, 11.6	7.2, 19.4	7.8, 11.7
R_1, wR_2 [I>2σ(I)], %	4.1, 10.4	6.3, 18.1	4.5, 10.3

Table S3. Crystal data of compounds **10** and **11**

**Compound**	**10a**	**10c**	**10d**	**11c**	**11d**
Depository code	15srv091	15srv282	16srv262	16srv268	16srv431
CCDC	1551620	1551621	1551622	1551623	1551624
Formula	$C_{25}H_{15}B_3F_9NO_3$	$C_{32}H_{33}B_3N_2O_3$	$C_{46}H_{51}B_3N_4O_3$	$C_{28}H_{22}B_2Cl_2O_5$	$C_{28}H_{22}B_2I_2O_5$
$D_{calc.}$/ g cm^{-3}	1.565	1.205	1.203	1.397	1.774
μ/mm^{-1}	0.15	0.08	0.07	0.30	2.39
Formula Weight	580.81	526.03	740.33	530.97	713.87
T/K	120	120	120	120	120
Crystal System	triclinic	triclinic	monoclinic	triclinic	triclinic
Space Group	P-1 (# 2)	P-1 (# 2)	$P2_1/c$ (# 14)	P-1 (# 2)	P-1 (# 2)
a/Å	10.3720(5)	11.2396(4)	21.2120(9)	10.4296(5)	10.6350(5)
b/Å	11.1795(5)	15.2054(6)	11.2705(5)	11.1470(6)	11.4814(5)
c/Å	12.3123(6)	17.9361(7)	18.8494(8)	11.7996(6)	12.2457(6)
α/°	106.545(1)	89.733(2)	90	84.748(2)	83.139(2)
β/°	111.492(1)	72.020(2)	114.886(2)	87.958(2)	87.095(2)
γ/°	96.849(2)	84.329(2)	90	67.536(2)	64.182(2)
V/Å^3	1232.74(10)	2900.27(19)	4087.9(3)	1262.39(11)	1336.36(11)
Z	2	4	4	2	2
Wavelength/Å	0.71073	0.71073	0.71073	0.71073	0.71073
θ_{max}/°	30.1	27.6	26.0	33.2	30.0
Measured refl.	27452	55370	68095	28237	21913
unique refl.	7267	13416	8022	9620	7764
Refl. with I>2σ(I)	6223	10461	6020	7311	6143
R_{int}	2.4	4.4	5.6	3.0	2.9
Parameters, restraints	379, 0	753, 0	584, 604	334, 309	334, 0
Δρ max/min, eÅ^{-3}	0.46, -0.22	0.33, -0.29	0.31, -0.28	0.48, -0.45	0.83, -0.60
R_1, wR_2 (all data), %	4.3, 10.0	6.2, 10.9	7.4, 11.7	6.4, 11.1	4.5, 5.8
R_1, wR_2 [I>2σ(I)], %	3.6, 9.5	4.5, 10.1	5.0, 10.5	4.3, 10.2	2.9, 5.4

Table S4. Crystal data of other borinic acid – ethanolamine complexes

**Compound**	**ESI1**	**ESI2**	**ESI3**
Depository code	15srv265	16srv036	16srv264
CCDC	1563951	1563952	1563953
Formula	$C_{14}H_{10}BF_6NO$	$C_{14}H_{14}BCl_2NO$	$C_{14}H_{12}BCl_2F_2NO$
$D_{calc.}$/ g cm^{-3}	1.586	1.453 (1.464 [a])	1.493
μ/mm^{-1}	0.15	0.47 (0.61 [a])	0.46
Formula Weight	333.04	293.97 (296.19 [a])	329.96
T/K	120	120	120
Crystal System	monoclinic	orthorhombic	monoclinic
Space Group	$P2_1/c$	$P2_12_12_1$	$P2_1/c$
a/Å	9.7608(4)	8.9466(7)	23.6856(14)
b/Å	18.4124(10)	10.7958(9)	18.9704(11)
c/Å	15.5249(9)	13.9126(12)	9.8173(6)
β/°	90.202(5)	90	92.977(2)
V/Å^3	2790.1(2)	1343.76(19)	4405.2(5)
Z	8	4	12
λ/Å	0.71073	0.71073	0.71073
θ_{max} /°	25	33.6	32.6
measured refl.	16064	35671	112010
unique refl.	4869	5299	16035
refls with I>2σ(I)	4003	5053	12293
R_{int} , %	6.4	3.0	6.0
Parameters	416	180	592
$\Delta\rho$ max/min, eÅ^{-3}	0.60, -0.60	0.55, -0.42	0.83, -0.64
R_1, wR_2 (all data), %	8.9, 21.1	2.9, 7.4	6.8, 11.1
R_1, wR_2 [I>2σ(I)], %	7.5, 18.9	2.7, 7.3	4.5, 10.2

[a] assuming contamination, as $C_{14}H_{14}BBr_{0.05}Cl_{1.95}NO$

5. References

1. R. L. Letsinger, I. Skoog, *J. Am. Chem. Soc.* 1955, **77**, 2491.
2 T. M. El Dine, J. Rouden, J. Blanchet, Chem. Commun. 2015, 51, 16084–16087.
3 L. Krause, R. Herbst-Irmer, G. M. Sheldrick, D. Stalke, *J. Appl. Crystallogr.*, 2015, **48**, 3-10.
4 L. J. Bourhis, O. V. Dolomanov, R. J. Gildea, J. A. K. Howard, H. Puschmann, *Acta Crystallogr. Sect. A,* 2015, **71**, 59-75.
5 a) L. Palatinus, G. Chapuis, *J. Appl. Crystallogr.*, 2007, **40**, 786-790; b) L. Palatinus, A. van der Lee, *J. Appl. Crystallogr.*, 2008, **41**, 975-984; c) L. Palatinus, S. J. Prathapa, S. van Smaalen, *J. Appl. Crystallogr.*, 2012, **45**, 575-580.
6 G. M. Sheldrick, *Acta Crystallogr. Sect. C,* 2015, **71**, 3-8.
7 G. M. Sheldrick, *Acta Crystallogr. Sect. A,* 2008, **64**, 112-122.
8 O. V. Dolomanov, L. J. Bourhis, R. J. Gildea, J. A. K. Howard, H. Puschmann, *J. Appl. Crystallogr.*, 2009, **42**, 339-341
9 H. D. Flack, *Acta Crystallogr. Sect. A*, 1983, **39**, 876-881.
10 S. Parsons, H. D. Flack, T. Wagner, *Acta Crystallogr. Sect. B*, 2013, **69**, 249-259.
